# Stereochemical Aspects in the Context of the Structure–Activity Relationship of Chlorido[*N*,*N*′‑bis(chloro/bromosalicylidene)-1,2-diphenyl-1,2-diaminoethane]iron(III) Complexes

**DOI:** 10.1021/acs.jmedchem.5c03253

**Published:** 2026-04-17

**Authors:** Astrid Dagmar Bernkop-Schnürch, Stefanie Schwarz, Philipp Rigo, Daniel Leitner, Mostafa Alilou, Martin Hermann, Michael Seidl, Magnus Andre Kiechle, Sofie Hanifle, Stephan Hohloch, Brigitte Kircher, Ronald Gust

**Affiliations:** † Department of Pharmaceutical Chemistry, Institute of Pharmacy, CCB—Center for Chemistry and Biomedicine, 27255 University of Innsbruck, Innrain 80-82, 6020 Innsbruck, Austria; ‡ Department of Internal Medicine V (Hematology and Oncology), Immunobiology and Stem Cell Laboratory, Medical University of Innsbruck, Anichstraße 35, 6020 Innsbruck, Austria; § Department of General, Inorganic and Theoretical Chemistry, University of Innsbruck, Innrain 80-82, 6020 Innsbruck, Austria; ∥ Department of Pharmacognosy, Institute of Pharmacy, University of Innsbruck, Innrain 80-82, 6020 Innsbruck, Austria; ⊥ Department of Anesthesiology and Critical Care Medicine, Medical University of Innsbruck, Anichstraße 35, 6020 Innsbruck, Austria; # Department of Pharmaceutical Technology, Institute of Pharmacy, CCB—Center for Chemistry and Biomedicine, University of Innsbruck, Innrain 80-82, 6020 Innsbruck, Austria; ⊗ Tyrolean Cancer Research Institute, Innrain 66, 6020 Innsbruck, Austria

## Abstract

In this structure–activity relationship study, chlorido­[*N,N′*-bis­(chloro/bromosalicylidene)-1,2-diphenyl-1,2-diaminoethane]­iron­(III) complexes differing in (i) the configuration of the 1,2-diphenylethane backbone ((*RS*), (*RR*/*SS*), or (*SS*)) and (ii) the halogen substituents at the salicylidene residue in positions 5 (Cl (**1a**–**c**) or Br (**2a**–**c**) or 3-Br,5-Cl (**3a**–**c**)) were investigated. All complexes were fully characterized and showed high stability with regard to ligand racemization. The antitumor activity was dependent on the configuration and the substitution pattern of the salicylidene moieties. (*RS*)-Configured complexes were nearly inactive, whereas (*RR/SS*)- or (*SS*)-configured complexes displayed almost identical efficacies. **1a**–**c** induced oxidative stress, but only **1b** and **1c** caused extensive lipid oxidation and ferroptosis as part of the mode of action. Exchange of the 3-Cl substituents for 3-Br only marginally changed the biological effects, whereas the introduction of 3-Br, 5-Cl substituents led to a loss of activity. The differences in effectiveness result from the spatial structure caused by the 1,2-diphenylethane skeleton.

## Graphical abstract

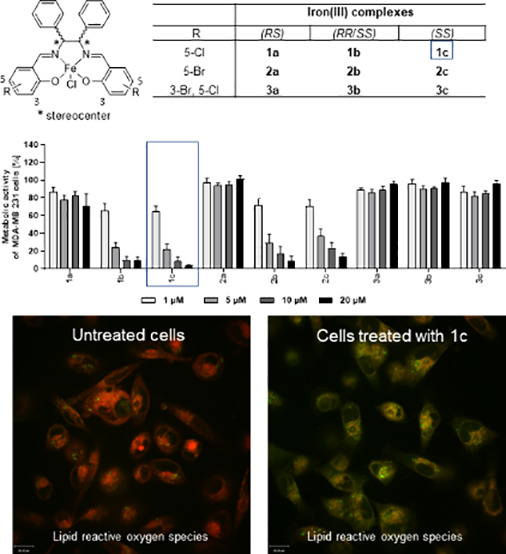

## 1 Introduction

Stereochemistry is a critical aspect in drug development, as diastereomers and enantiomers may exhibit distinct efficacies, pharmacodynamics, pharmacokinetics, toxicities, and the kinetics of plasma disposition and urinary excretion.^1^ This applies not only to organic molecules, but also to metal-based drugs.

A well-known example is the chemotherapeutic agent Oxaliplatin, whose biological effect is controlled by the 1,2-diaminocyclohexane (DACH) ligand and its stereochemistry.^2,3^ The complex with the (*RR/SS*)-configured ligand is far superior to its (*RS*)-configured diastereomer. Further optimization of the activity is achieved by separation of the enantiomers. The approved active drug is [(*RR*)-1,2-diaminocyclohexane]­oxalatoplatinum­(II) (Scheme 1A).^3,4^

**Scheme 1:**
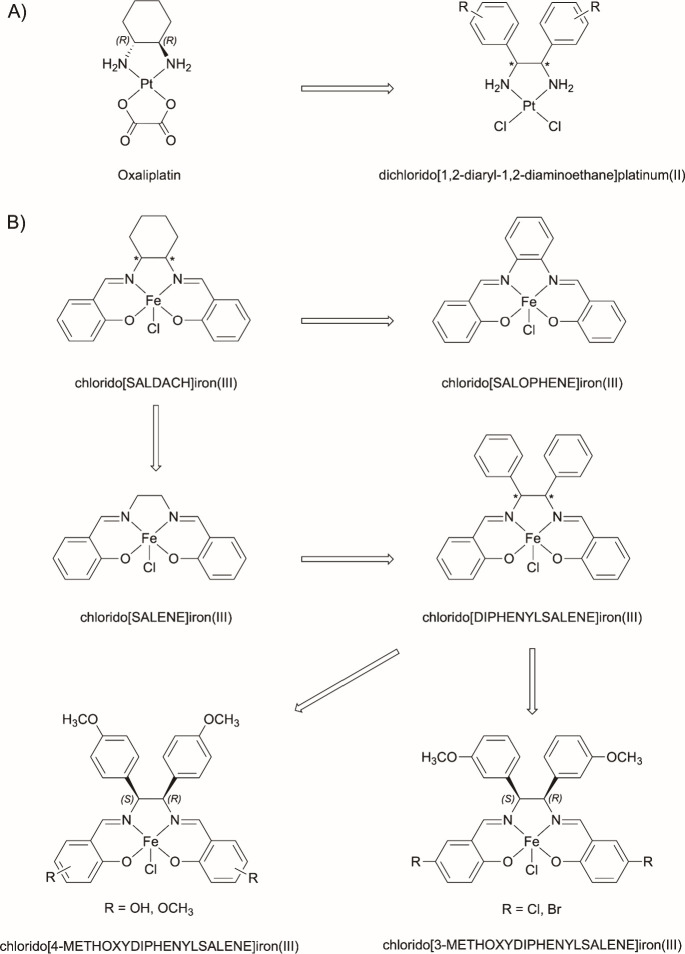
Overview of Lead Structures Used. A: Platinum­(II) Complexes. B: Chlorido iron­(III) Complexes.^a^

a The asterisks indicate the stereocenters.

The varying antitumor effects of the Oxaliplatin stereoisomers can be attributed, on the one hand, to steric influences when approaching the target bases of the deoxyribonucleic acid (DNA) (difference between the diastereomers) and, on the other hand, to the formation of diastereomeric adducts through the complexes with (*RR*)- or (*SS*)-configured ligands on the DNA.^5^

The positive effects with DACH as a carrier ligand for platinum complexes prompted us to derivatize it to *N,N′*-disalicylidene-1,2-diaminocyclohexane (SALDACH), which can coordinate iron­(III). It was assumed that the resulting stereoisomeric chlorido­[SALDACH]­iron­(III) complexes (Scheme 1B)^6,7^ have a different mode of action than platinum complexes and may therefore enable the treatment of tumors after failure of platinum-containing chemotherapy.^7^

It has already been demonstrated that related iron­(III) complexes induce oxidative stress through the formation of reactive oxygen species (ROS) in mitochondria^8–10^ and lipids^9–12^ and thereby cause apoptosis,^8,9,11,13–15^ necroptosis,^8–12,16^ and ferroptosis.^8–12,16^ Ferroptosis, in particular, represents an interesting form of iron-induced cell death that is involved in a number of diseases.^17,18^

Isomeric chlorido­[SALDACH]­iron­(III) complexes reduced the proliferation and induced apoptosis of leukemia and lymphoma cells^6^ and the generation of ROS seems likely. The antiproliferative effects, however, were only slightly influenced by the configuration and conformation of the DACH substructure.^7^ Interestingly, also the chlorido­[*N*,*N*′-disalicylidene-1,2-diaminoethane]­iron­(III) core (chlorido­[SALENE]­iron­(III), Scheme 1B) possessed cytotoxic activity, making it a suitable lead structure for drug design.^15^

By introducing aryl rings at positions 3 and 4, a carrier ligand was formed in analogy to antitumor-active [1,2-diaryl-1,2-diaminoethane]­platinum­(II) complexes (Scheme 1B) and allowed the investigation of the biological effect as a function of the substituents in the aromatic rings and of the configuration at the 1,2-diarylethane bridge.^10,12^

The influence of substituents at the chlorido­[*N,N′*-disalicylidene-1,2-diphenyl-1,2-diaminoethane]­iron­(III) complex (chlorido­[DIPHENYLSALENE]­iron­(III), Scheme 1B) has already been studied on the example of complexes with (*RS*)-configured ligands. We have recently reported on fluorescent chlorido­[(*RS*)-*N,N′*-disalicylidene-1,2-bis­(4-methoxyphenyl)-1,2-diaminoethane]­iron­(III) complexes (Scheme 1B), whose activity depended on the type (methoxy and hydroxyl) and the position of the substituents at the salicylidene moieties.^12^

In the case of the 3-methoxyphenyl analogous (Scheme 1B), halides at positions 5 increased the biological activity. Especially, complexes with 5-Cl or 5-Br substituents showed enhanced antiproliferative and antimetabolic effects. They also induced cell death through both ferroptosis and necroptosis.^10^

The next step in investigating the structure–activity relationship (SAR) of chlorido­[DIPHENYLSALENE]­iron­(III) complexes was therefore to determine the influence of the stereochemistry at the 1,2-diarylethane bridge on the antitumor activity. To allow a better correlation with the structure, and to increase the cytotoxic activity, 5-chloro, 5-bromo or 3-bromo-5-chloro substituents were introduced to the salicylidene residues.

The related (*RS*)-, (*RR/SS*)- and (*SS*)-configured DIPHENYLSALENEs were synthesized and coordinated to iron­(III). All ligands and complexes were carefully characterized. The antitumor activity of the complexes was investigated in breast cancer (MDA-MB 231), Cisplatin-sensitive (A2780) and Cisplatin-resistant (A2780cis) ovarian carcinoma, leukemia (HL-60) as well as nonmalignant stroma (HS-5) cell lines, using a broad range of assays analyzing cell proliferation, cell migration, and cell viability.

## 2 Results and Discussion

### 2.1 Chemistry and Structural Evaluation

The synthesis of the chlorido­[DIPHENYLSALENE]­iron­(III) complexes **1a**–**c** to **3a**–**c** followed a well-established synthetic route (Scheme 2).^10,12^ Diastereomerically pure (*RS*)-, (*RR*/*SS*)-, and (*SS*)-configured 1,2-diphenyl-1,2-diaminoethanes were used as educts. They were reacted with 5-chlorosalicyl-aldehyde, 5-bromosalicylaldehyde, or 3-bromo-5-chlorosalicyl-aldehyde to yield the respectively substituted *N,N′*-disalicylidene-1,2-diphenyl-1,2-diaminoethanes **L1a**–**c** to **L3a**–**c**.

**Scheme 2:**
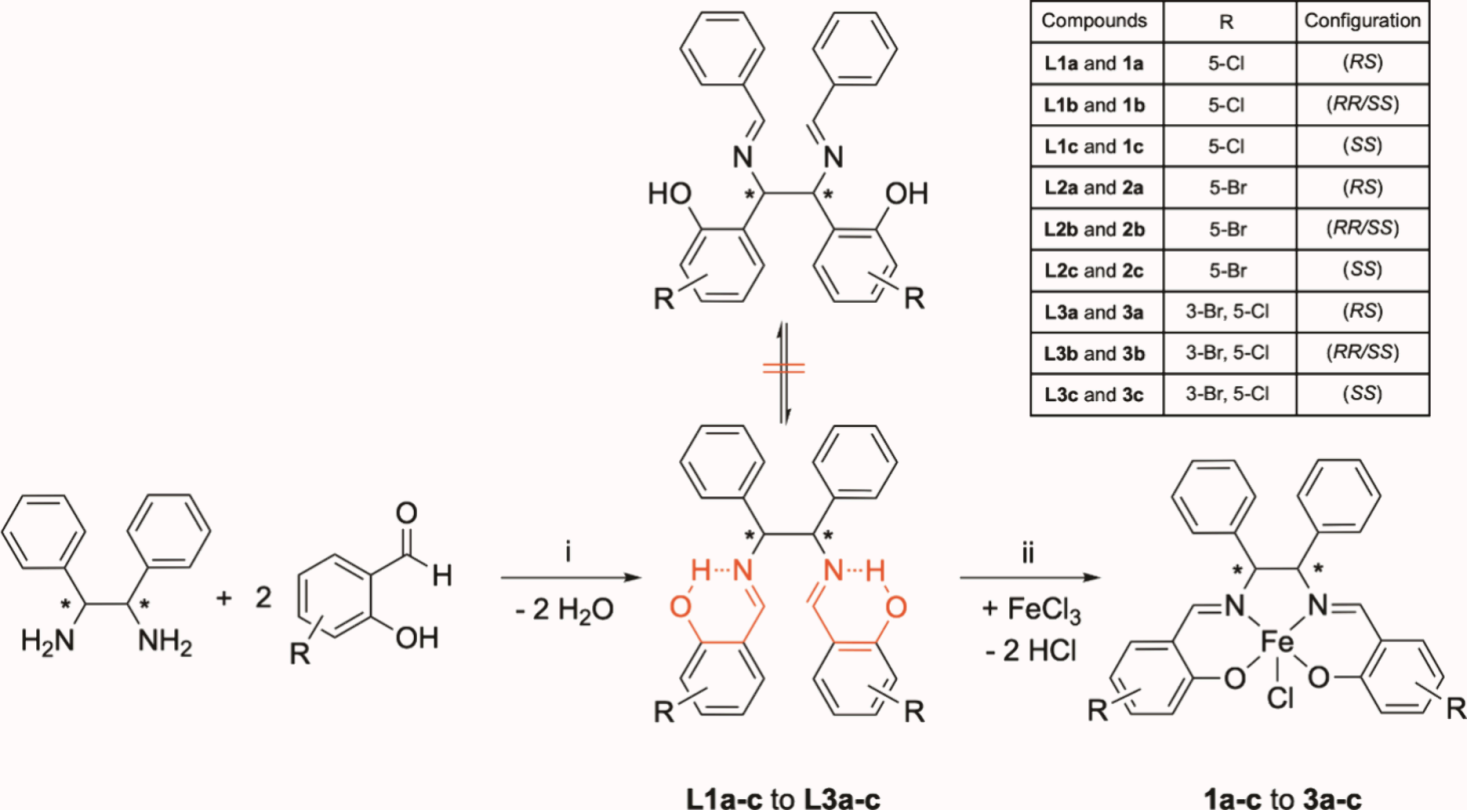
Synthesis Pathway for the Formation of Chlorido­[DIPHENYLSALENE]­iron­(III) Complexes with (*RS*)-, (*RR*/*SS*)-, or (*SS*)-Configured *N,N′*-Disalicylidene-1,2-diphenyl-1,2-diaminoethane Ligands.^a^

a The asterisks indicate the stereocenter. Conditions: (i) acetonitrile anhydrous, reflux, 48 h; (ii) ethanol anhydrous, reflux, 0.5–2 h.

All Schiff bases were characterized by nuclear magnetic resonance (NMR, ^1^H and ^13^C) as well as Fourier transform infrared (FT-IR) spectroscopy. The spectra are submitted as Supporting Information ().

It is worth mentioning that *N,N’*-dibenzylidene-1,2-diphenyl-1,2-diaminoethanes can principally undergo a [3,3′]-sigmatropic (diaza-Cope) rearrangement in the reaction mixture (Scheme 2).^19,20^ In case of the unsubstituted derivative, an equilibrium mixture of the (*RS*)-configured compound and the (*RR/SS*)-enantiomers was formed, regardless of which diastereomer of the 1,2-diphenyl-1,2-diaminoethane was used as educt. Interestingly, 2-OH substituents at the benzylidene moieties (→ salicylidene) stabilized the structure. The ^1^H NMR spectra of **L1a**–**c**, **L2a**–**c**, and **L3a**–**c** () showed only signals of one diastereomer, corresponding to the 1,2-diphenyl-1,2-diaminoethane used.

The reason for the increased stability were the strong intramolecular H-bridges of the 2-OH groups to the imine nitrogen atoms (see Scheme 2, marked in red), which prevented the diaza-Cope rearrangement to *N,N’*-dibenzylidene-1,2-bis­(2-hydroxyphenyl)-1,2-diaminoethane (Scheme 2).

The O–H····N=C interactions were proven by FT-IR spectroscopy. The O–H stretching vibrations were strongly broadened and thus no longer detectable in the range from 2500 to 3800 cm^–1^. The frequency of the characteristic C=N vibrations occurred at 1620–1631 cm^–1^ ().

Crystal structure analyses of the (*RR*)- and (*SS*)-configured *N,N’*-disalicylidene-1,2-diphenyl-1,2-diaminoethanes^21,22^ showed that the six-membered hydrogen-bonded heterocycles were almost perfectly planar and arranged in an energetically favored antiperiplanar orientation.

The compounds were also investigated by electronic circular dichroism (ECD) spectroscopy. As expected, no signals were detectable in the ECD spectra of **L1a**–**L3a** since they were achiral compounds. Also **L1b**–**L3b** only induced a flat baseline confirming the presence of racemic mixtures with a 1:1 distribution of enantiomers (data not shown).

Due to the Cotton effect, the educt (*SS*)-1,2-diphenyl-1,2-diaminoethane exhibited two negative signals at 206 and 220 nm (). The ECD spectra of **L1c**–**L3c** displayed negative signals at about 210, 235, and 250 nm, and positive ones at about 220, 285, and 340 nm (Figure 1A).

**Figure 1:**
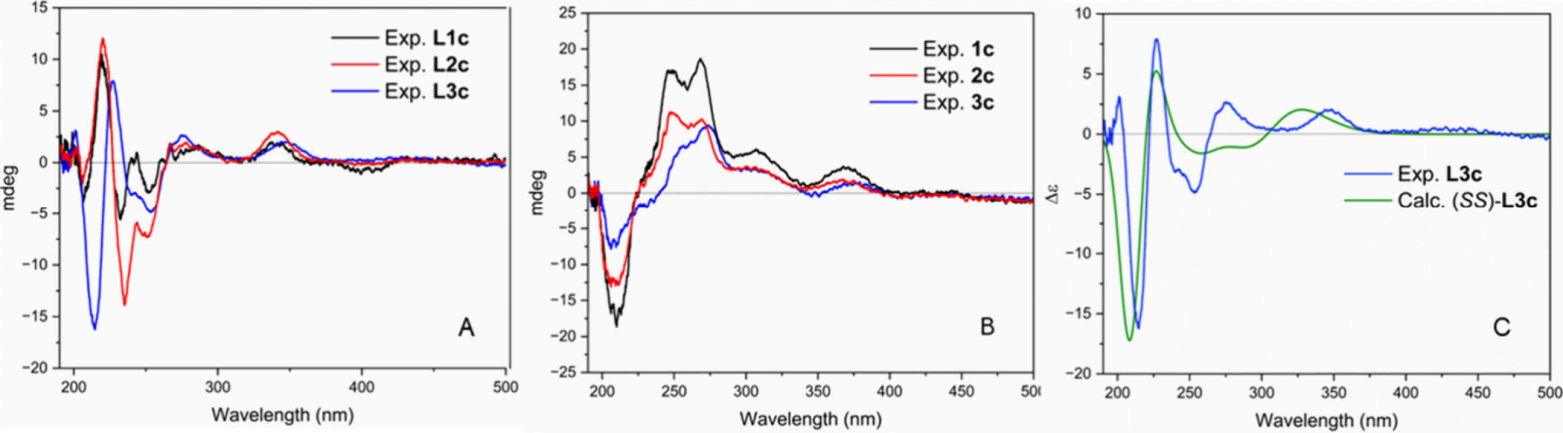
Superimposed experimental ECD spectra (measured in methanol (MeOH)) of three optically active A: ligands (**L1c**–**L3c**) and B: complexes (**1c**–**3c**). C: Experimental versus calculated ECD spectra of **L3c** at TD-DFT/B3LYP/6-31G­(d,p)/CPCM level in MeOH.

For **L1c–L3c** energetically preferred conformations exist, which can be converted into each other by rotation around the ethane bridge (Scheme 3A, the assignment to synclinal and antiperiplanar refers to the salicylidene residues).

**Scheme 3:**
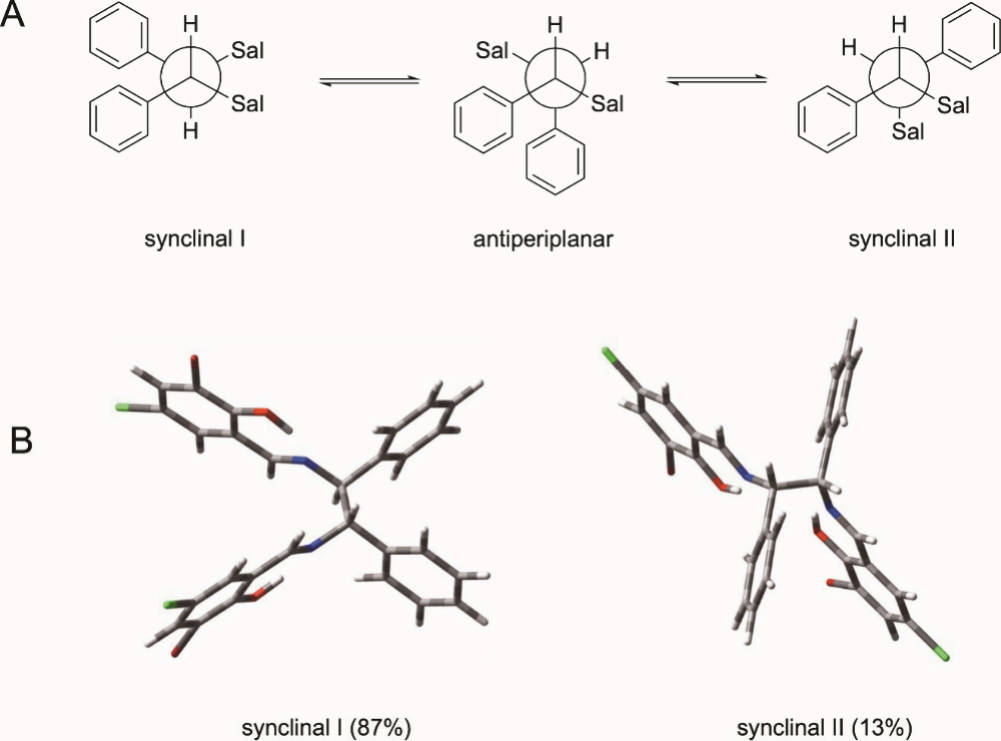
A: Conformations: Synclinal I and II Refer to the Arrangement of the Salicylidene (Sal) Units. B: Geometrical Optimization and Distribution of the Most Stable Conformations of the (*SS*)-Configured *N,N′*-Disalicylidene-1,2-diphenyl-1,2-diaminoethane **L3c**, at DFT/B3LYP/6-31G­(d,p) Level in the Gas Phase.

In the conformation synclinal I, the phenyl and salicylidene (Sal) residues are each synclinally arranged and can sterically evade each other. The antiperiplanar conformation is characterized by the fact that the larger salicylidene units are located in opposite directions. In synclinal II, the residues are similarly oriented, though here the phenyl rings are antiperiplanar and the Sal synclinal.

According to the theoretical calculations performed for **L3c** (see Section 4.1.4), conformations with synclinally arranged salicylidene moieties in the gas phase with a distribution of 87% (synclinal I): 13% (synclinal II) are predominant (Scheme 3B). These spatial structures are preferred over the antiperiplanar conformation, because the π-orbitals of the imine double bonds overlap and form a quasi-folded six-membered ring. The phenyl rings are energetically favorable in bisequatorial (synclinal I) or bisaxial positions (synclinal II). A formal inversion of the “six-membered ring” resulting in an equilibrium of synclinal I and II is possible, with the equatorial standing phenyl rings favoring synclinal I.

Subsequently, the ECD spectrum of **L1c** was calculated at TD-DFT/B3LYP/6-31G­(d,p)/CPCM level in MeOH.^23^ As depicted in Figure 1C, the theoretical ECD spectrum is in a good agreement with the experimental one and therefore confirms the (*SS*)-configuration.

Reaction of **L1a**–**c** to **L3a**–**c** with iron­(III) chloride led to the formation of the chlorido­[DIPHENYLSALENE]­iron­(III) complexes **1a**–**c** to **3a**–**c** (Scheme 2).^10,12^ The iron­(III) was bound via the imine nitrogens and the phenolic oxygens forming one five-membered and two six-membered chelate rings, with chloride at the fifth coordination site. In accordance, the binding order and strength of the imine bond was reduced, resulting in a shift of the N=C stretching vibration to 1523–1530 cm^–1^ (**1a**–**c** and **2a**–**c**) and 1512–1519 cm^–1^ (**3a**–**c**). The v̅ (C–O) appeared at 1177–1180 cm^–1^ (**1a**–**c** and **2a**–**c**) or 1160–1170 cm^–1^ (**3a**–**c**) ().

High-resolution mass spectrometry (HR-MS) indicated in the positive mode in each case the [DIPHENYLSALENE]­iron­(III) cation and elemental analyses (see Experimental Section) confirmed that chloride is present as a fifth ligand. The purity of **1a**–**c** to **3a**–**c** was additionally determined by high performance liquid chromatography (HPLC) and indicated a purity higher than 95% ( and ).

All complexes were paramagnetic high-spin Fe­(III) compounds, as evidenced by electron paramagnetic resonance spectroscopy (EPR; ), which is in accordance with previously reported [SALENE]­iron­(III) complexes.^10–12^ The revealed g-values from the spectra are listed in the Experimental Section.

The ECD spectra of **1a**–**3a** and **1b**–**3b**, like those of their ligands, did not reveal any significant transitions (data not shown). This again confirmed the presence of (*RS*)-configured compounds for **1a**–**3a** or a racemic (*RR/SS*) mixture for **1b**–**3b**. In contrast, **1c**–**3c** were optically active ((*SS*)-configuration) with ECD spectra significantly different from those of the ligands (compare Figure 1A and 1B). Thus, in contrast to the free ligands, the complexes exist in a stable conformation, which seems to be the reason for the significantly altered ECD spectra. The absolute configurations of **1c** and **3c** were further confirmed to be (*SS*)-configured by X-ray crystallographic analysis (Figure 2), and the configuration of **2c** was established by comparing its ECD spectrum with those of **1c** and **3c**.

**Figure 2:**
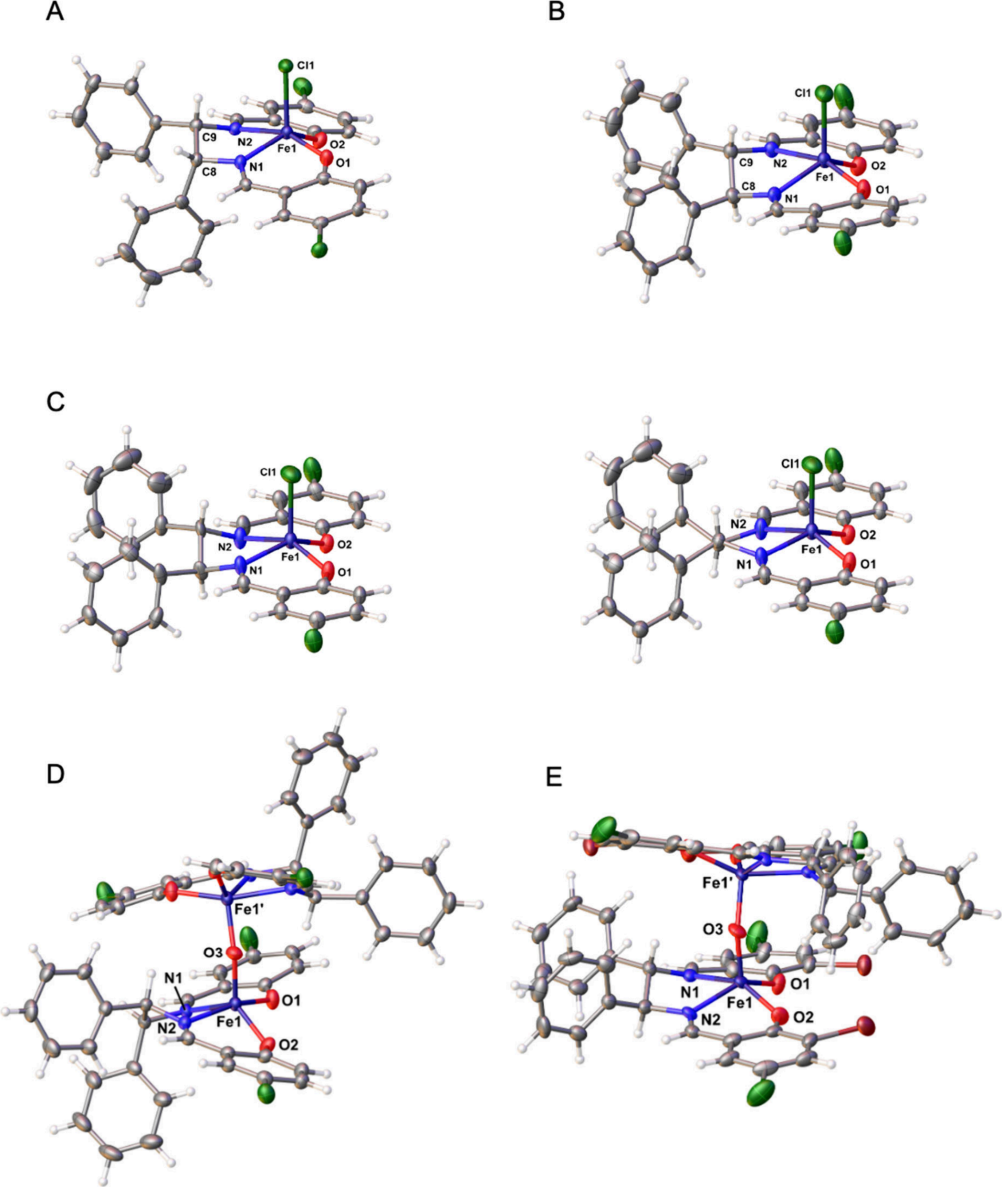
Molecular structures of compounds **1a** (A and D), **1c** (B), **1b** (C; left (*SS*) and right (*RR*)) and **3c** (E). Crystals were grown from DCM (A to C) or DMSO (D and E). Thermal ellipsoids are set at 50% probability level. Solvent molecules are omitted for clarity.

In order to get a more detailed insight into the spatial structure of the ligands bound to iron­(III), it was tried to crystallize the complexes from dichloromethane (DCM). The X-ray structures of **1a**–**c** are depicted in Figure 2 (A, B, and C) as examples. Further details and available X-ray structures are submitted as Supporting Information (, ).

As can be seen from Figure 2A–C, the salicylidene units formed a slightly distorted planar N,O coordination sphere around the metal ion. The ethane bridge between the two building blocks is part of a flexible 5-membered chelate ring, which can adapt a δ- or λ-conformation as found in [1,2-diaryl-1,2-diaminoethane]­platinum­(II) complexes.^24–27^

Complexes **1a** and **1c** crystallized with C8 below and C9 above the N2O2 plane formed upon coordination to iron­(III). Chloride, as the fifth ligand, occupies the axial position above the iron­(III) (Figure 2A–C), if crystallized from DCM.

In complex **1a** with the (*RS*)-configured ligand, the phenyl rings are synclinally arranged with the phenyl ring at C8 positioned axially in opposite to the Cl ligand coordinated to Fe­(III). The phenyl ring at C9 is equatorial standing. Other conformers (λ-conformation) or possible isomers (Fe–Cl and C8-phenyl ring at one side) were not observed.

However, it must be pointed out that these are structures in the solid state. In solution, exchange of the chloride ligand at the Fe­(III) atom^9^ and conformational changes are possible. It has already been shown that 1,2-diphenyl-1,2-diaminoethane bearing 5-membered chelate rings at various metal ions undergo a fast interconversion between the δ- and λ-conformation, if it is (*RS*)-configured.^24–27^

The 3-dimentional structures of **1a** and **1c** are very similar, with the exception that the phenyl ring at C8 of **1c** is in an equatorial position due to the (*S*)-configuration.

Generally, in complexes with (*SS*)- and (*RR*)-configured ligands, the phenyl rings can be bisequatorially or bisaxially arranged. However, the X-ray structures of **1c** (Figure 2B) and also **1b** (Figure 2C) document only the presence of the conformation with equatorially positioned phenyl rings. This spatial structure can also be assumed in solution. Investigations on (*SS/RR*)-1,2-diphenyl-1,2-diaminoethane chelates confirm that conformations with bisequatorially positioned rings predominate. Dynamic effects (δ/λ-transformation) of the chelate ring could not be observed.

Crystals of **1a** and **3c** could also be obtained from dimethyl sulfoxide (DMSO). This was of interest, because DMSO was used as solvent to prepare stock solutions of the complexes for biological testing and an exchange of the axial ligand by a DMSO molecule, as observed in [SALOPHENE]­iron­(III) complexes (Scheme 1),^9^ could not be excluded. Interestingly, this adduct was not observed in this study. Rather crystals of μ-oxo dimers (Figure 2D and 2E) were isolated. Such derivatives are formed under basic conditions or in the presence of traces of oxygen.

In the dimers of **1a**, the bridging oxo ligand is located at distances of 1.7708(6) Å to the iron­(III) centers, resulting in an Fe–O–Fe bond angle of 153.95(15)° (Figure 2D). These metric parameters are typical for μ-oxo dimers.^28,29^ The N2O2 environments at the iron­(III) centers are slightly disturbed and the 5-membered chelate rings adopt the λ-conformation.

Complex **3c** with (*SS*)-configured ligand crystallized in a comparable μ-oxo form with Fe–O distances of 1.767(4) Å and an Fe–O–Fe angle of 150.0(8)°, but with δ-conformation of the 1,2-diphenyl-1,2-diaminoethane chelates (Figure 2E). It should be mentioned that in case of **3c** a second molecule of the complex was present in the asymmetric unit with Fe–O distances of 1.764(2) Å and an Fe–O–Fe angle of 170.9(8)°. This documents a high flexibility of the μ-oxo bridge.

The μ-oxo iron­(III) dimers are fairly stable species, but they can be split by high chloride ion concentrations into chlorido iron­(III) or by low pH values^30^ into aqua/hydroxo iron­(III) monomers. Therefore, the stability of **1a**–**c** in phosphate-buffered saline (PBS, 100 mM) and Roswell Park Memorial Institute (RPMI) 1640 medium supplemented with 10% fetal bovine serum (FBS; represents the cell-culture medium) was studied using HPLC.

The chromatograms of the complexes dissolved in methanol, which were used to determine the purity (), showed well-defined peaks with retention times (Rt) of 8.7–8.8 min. As with [SALOPHENE]­iron­(III) complexes stored in the same solvent, the corresponding chlorido iron­(III) species is very likely to be present.

Of more interest are investigations with stock solutions in DMSO (10 mM), in which the μ-oxo dimers are formed. These solutions were diluted to 100 μM in PBS or cell-culture medium and incubated at 37 °C.

Chromatograms () recorded after incubation for 2 and 24 h in PBS contain, in addition to the DMSO peak, peaks of **1a**–**c** at Rt = 8.8–9.1 min, which were very similar to that detected in MeOH. Only for **1c** a small fraction (10.4%) of another more polar compound was present at Rt = 6.33 min. Extended incubation over 72 h () increased the area under the curve of this peak to 25.4%, suggesting that it is a degradation product of **1c**. Also, the chromatograms of **1a** and **1b** exhibited after 72 h comparable peaks with 30.9% and 45.1%, respectively.

After 2 h of incubation in cell-culture medium at 37 °C, the chromatograms showed besides ingredients of the medium (Rt < 6 min ()) exclusively peaks of the initial complexes with Rt = 8.75–8.80 min (). Incubation for 24 h led to degradation of 39.0% (**1a**), 13.1% (**1b**), and 22.5% (**1c**), respectively (). Only in case of **1c**, a further degradation to 41.6% took place during an incubation time of 72 h ().

These data suggest that, if μ-oxo dimers were formed in the DMSO stock solution, they will be rapidly (<2 h) cleaved back into monomers by the high chloride ion concentration in PBS or cell-culture medium. These monomers would then degrade to a small extent over the following 24 h to a more polar species that could not be further characterized. However, demetalation can be ruled out, since no characteristic coloration appeared in either PBS or cell-culture medium after the addition of 5 mg potassium hexacyanoferrate­(II).^31^ However, this occurred together with a green to blue precipitate after addition of dilute hydrochloric acid (2 M) and incubation for several days.

### 2.2 Biology

#### 2.2.1 Antiproliferative and Antimetabolic Effects of Chlorido­[DIPHENYLSALENE]­iron­(III) Complexes

The potency of the chlorido­[DIPHENYLSALENE]­iron­(III) complexes to induce antiproliferative and antimetabolic activity was evaluated in two different assays, the [^3^H]-thymidine incorporation test and a modified 3-(4,5-dimethylthiazol-2-yl)-2,5-diphenyltetrazolium bromide (MTT) assay.

##### 2.2.1.1 [^3^H]-Thymidine Incorporation Test

The effect of the chlorido­[DIPHENYLSALENE]­iron­(III) complexes on cell activity during the S-phase of the cell cycle, especially during mitotic cell division, was evaluated by the [^3^H]-thymidine incorporation assay.^32^ Cells were treated with **1a**–**c** to **3a**–**c** at concentrations of 1 μM, 5 μM, 10 μM, and 20 μM for 72 h. [^3^H]-thymidine was added 16–18 h before the end of the experiment. After harvesting the cells, the [^3^H]-thymidine incorporation into DNA, expressed as counts per minute (cpm), was measured and compared with that of the untreated control.

Using MDA-MB 231 cells as an example, the effects of the complexes and the SAR are discussed. The results on the other cell lines are attached as Supporting Information ().

As obvious from Figure 3A, the complexes with (*RS*)-configured ligands (**1a**-**3a**) were nearly inactive. Only at a concentration of 20 μM, **1a** and **2a** slightly reduced cell proliferation to 75.4 ± 10.2% and 69.8 ± 16.5%, respectively.

**Figure 3:**
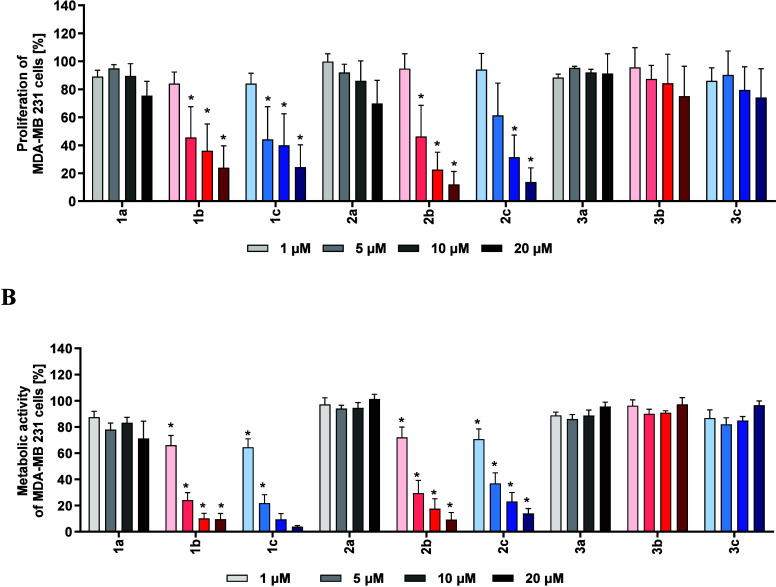
Proliferation and metabolic activity of MDA-MB 231 cells after treatment with **1a**–**c** to **3a**–**c**, determined with the [^3^H]-thymidine incorporation test (**A**) and the modified 3-(4,5-dimethylthiazol-2-yl)-2,5-diphenyltetrazolium bromide (MTT) assay (**B**). The cells were incubated for 72 h with the compounds at concentrations ranging from 1 μM to 20 μM. Data are presented as the mean of 5 independent experiments + SE. Proliferation in the presence of the solvent DMSO was 89.0 ± 7.3%. The metabolic activity in the presence of DMSO was 93.5 ± 2.3% (data not shown). Asterisks indicate statistical significance reduction (* *p* < 0.05) compared to untreated cells.

Replacing the (*RS*)-configured ligands in **1a** and **2a** for their racemic analogs (→ **1b** and **2b**) significantly increased the inhibitory effect of the complexes. At a concentration of 20 μM, **1b** reduced cell proliferation to 23.9 ± 15.7% and **2b** to 12.0 ± 9.3%. The configurational change was less effective for **3a**. Complex **3b** reduced the proliferation only to 75.1 ± 21.5%.

Remarkably, the use of the pure (*SS*)-configured ligands did not lead to an optimization of the activity compared to the racemic complexes. At a concentration of 20 μM, treatment with **1c**, **2c**, or **3c** resulted in cell proliferation rates of 24.3 ± 16.0%, 13.7 ± 10.1%, and 74.2 ± 20.5%, respectively.

As shown in Figure 3A, replacing 5-Cl for 5-Br at the salicylidene residues had only a negligible impact on antiproliferative activity of the complexes, whereas introducing an additional Br at position 3 (**2a**–**c** → **3a**–**c**) almost completely abolished the effect, independent of the configuration of the stereocenter.

Similar trends were observed in A2780cis () and HL-60 cells (). Interestingly, the complexes were marginally active in the A2780 cell line. Even at the highest concentration of 20 μM, the maximum observed antiproliferative effect was only 50% ().

The activity of the complexes on the nonmalignant stromal cell line HS-5 was also minimal. At a concentration of 20 μM, only compounds **1b**, **1c**, **2a**, and **2c** reduced cell proliferation by approximately 50–60% ().

##### 2.2.1.2 Metabolic Activity Assay

A colorimetric assay based on the reduction of a light-yellow tetrazolium salt to an orange formazan product was used to measure proliferation and, as the reduction relies on metabolically active mitochondria, it further provides a measure of the number of viable cells.^33^

Complexes **1b**, **1c**, **2b**, and **2c** reduced the metabolic activity of MDA-MB 231 cells slightly more effectively than the proliferation (Figure 3A/B). The derivatives with (*RS*)-configured ligands and with the 3-Br, 5-Cl substitution pattern were again completely inactive, even at the highest concentration (20 μM) tested.

For a better comparison of their effects, the IC_50_ values were determined for the active compounds. For this purpose, MDA-MB-231 cells were incubated in a further experiment with the complexes at concentrations ranging from 0.5 μM to 10 μM for 72 h and their metabolic activity was assessed. The chlorine-substituted complexes exhibited slightly higher antimetabolic activity than the bromine derivatives, with IC_50_ values of 2.6 ± 1.2 μM (**1b**) and 3.3 ± 1.1 μM (**1c**) versus 5.4 ± 1.2 μM (**2b**) and 5.7 ± 1.2 μM (**2c**), respectively.

These data confirmed the results of the [^3^H]-thymidine incorporation assay with respect to the ligand configuration and the substituents at the salicylidene moieties. The complexes were active in MDA-MB 231 (Figure 3B) and A2780cis cells (), with **1b** and **1c** again being more effective than **2b** and **2c**. The complexes showed markedly lower efficacy against the A2780 cell line. At the highest concentrations tested, the metabolic activity of the cells was reduced only by 20–40% ().

Interestingly, the complexes did not reduce the metabolic activity of HL-60 () and HS-5 cells (), despite causing cytostatic effects ().

#### 2.2.2 Scratch-Assay

The scratch assay is a simple *in vitro* method for assessing cell migration by observing and recording the movement of cells into the cell-free zone using transmitted light microscopy.^34,35^ It also provides insights into the onset of the effect of the complexes and the resulting morphological changes of the cells.

MDA-MB 231 cells were cultured to confluence of at least 90% and a physical scratch was made in the monolayer using a pipet tip. The cells were then treated with the complexes **1a**–**c** at concentrations of 1 μM and 10 μM and incubated at 37 °C in the Evident ScanR live-cell imaging system for 72 h. Pictures were taken initially 5 min after addition of the complexes (0 h) and subsequently every 2 h.

In any case, the initial scratch was visible (Figure 4, 0 h). Control cells strongly proliferated and migrated into a cell-free zone during 72 h (Figure 4, first row). Cells treated with **1a**–**c** at 1 μM () and **1a** at 10 μM (Figure 4, second row) showed continuous migration and proliferation (visible by an increased number of rounded cells over the monolayer), confirming the results of the [^3^H]-thymidine incorporation assay and the modified MTT assay.

**Figure 4:**
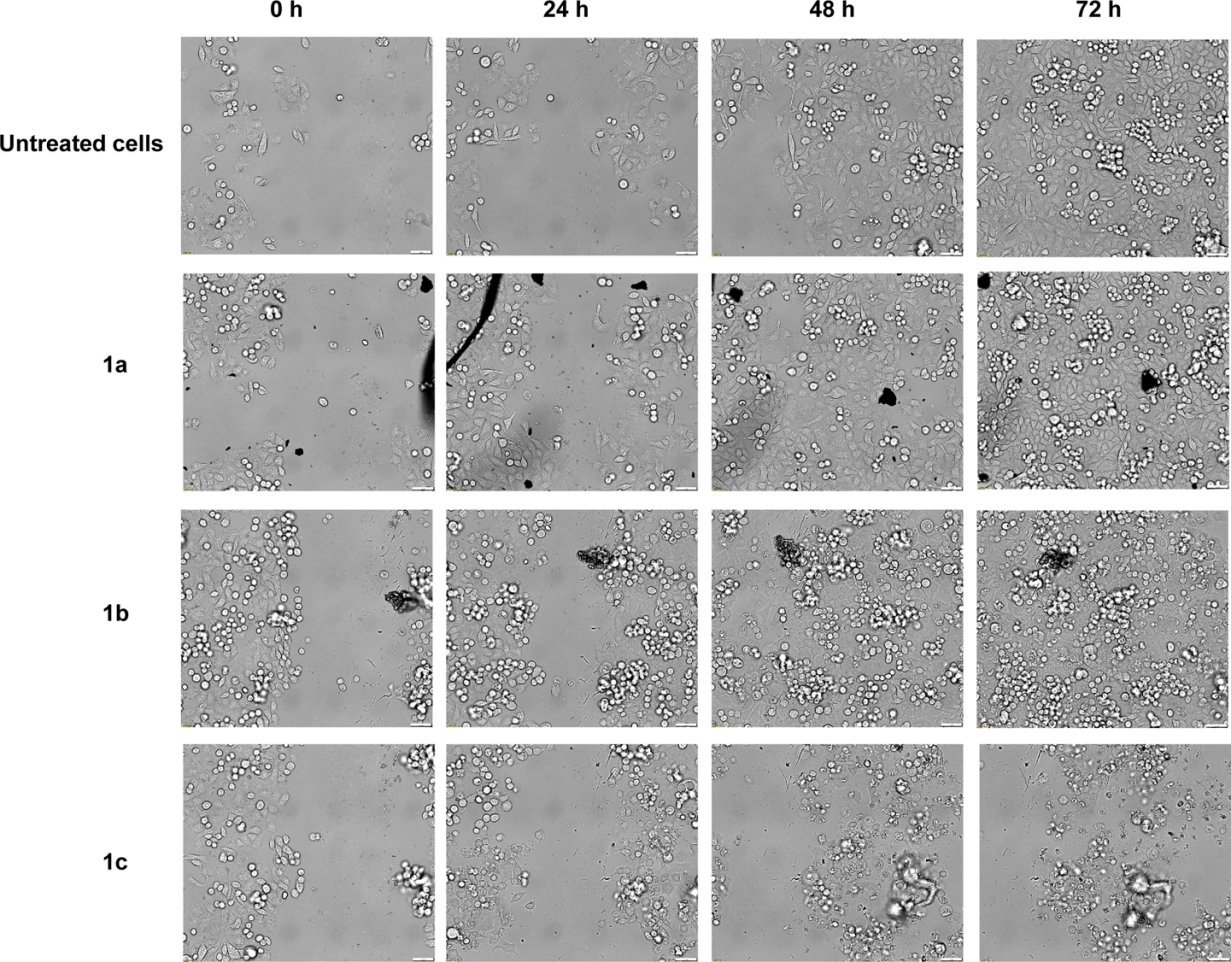
Cell migration investigated in a scratch assay. MDA-MB 231 were untreated (1st row) or incubated for 72 h with **1a** (2nd row), **1b** (3rd row) and **1c** (4th row), at a concentration of 10 μM, respectively. Pictures after 0 h, 24, 48, and 72 h (columns) are shown. Scale bar = 50 μM.

Incubation with **1b** at 10 μM did not completely prevent the migration during the first 24 h. After 48 h, most of the cells lost their spindle-shaped morphology and began to round up, showing signs of cell death (Figure 4, third row). This appearance remained almost unchanged for the following 24 h (Figure 4, third row).

With complex **1c** at 10 μM, no migration was visible or measurable (Figure 4, fourth row, ). Just after 24 h cell death started, resulting in no viable cells being detected after 48 and 72 h.

#### 2.2.3 Induction of Oxidative Stress in MDA-MB 231 Cells by Chlorido­[DIPHENYLSALENE]­iron­(III) Complexes

Mitochondria are a major source of ROS in cells, which contribute to a physiological cell signaling and regulation; however, when produced in excess, ROS can contribute to various pathological conditions.^36^ An imbalance between excessive ROS production and limited antioxidant defense mechanisms in the cells leads to oxidative stress, a harmful condition in the cells.

MitoTracker Red CM-H_2_XRos is commonly used to visualize the oxidative stress of cells by live confocal microscopy. The dye is nonfluorescent in its reduced state. After passing through the cell membrane, it is oxidized in actively respiring cells by ROS.^37^ This step makes the molecule red fluorescent and gives it a positive charge allowing accumulation in the negatively charged matrix of active mitochondria. There, the chloromethyl group of the dye reacts with free thiol groups of mitochondrial proteins and peptides, leading to the staining of the mitochondria.

MDA-MB 231 cells were treated with **1a**–**c** for 24 h, followed by incubation with MitoTracker Red CM-H_2_XRos for 15 min at 37 °C. The live confocal microscopy images of the untreated control group showed a basal ROS value (Figure 5, first column), which is due to normal cell metabolism and exposure of the cells to the test-induced stress situation.

**Figure 5:**
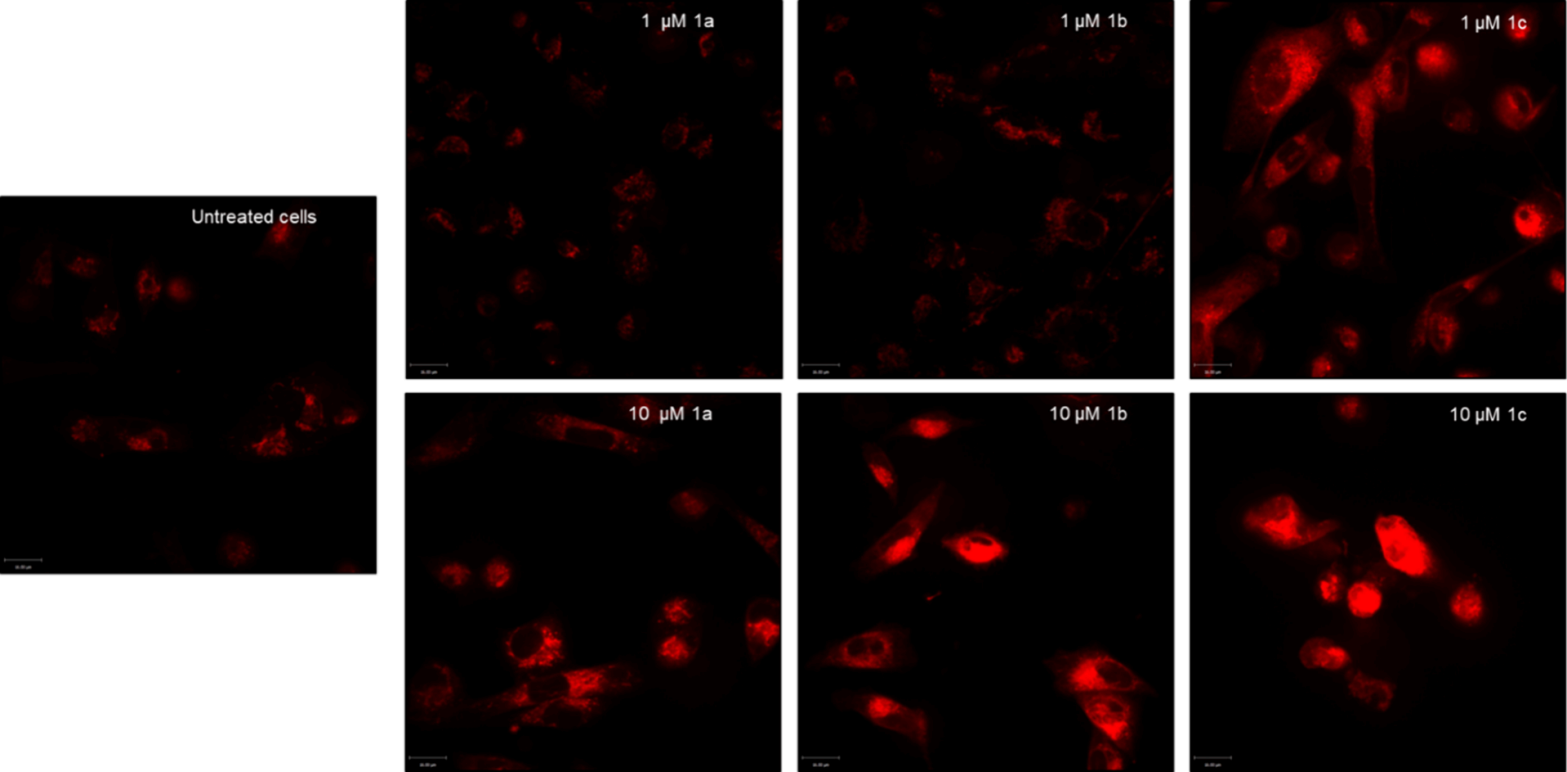
Detection of oxidative stress in MDA-MB 231 cells. The cells were incubated with **1a**–**c** at concentrations of 1 μM (upper row) and 10 μM (lower row) for 24 h, stained with MitoTracker Red CM-H_2_XRos (red), and analyzed by live confocal microscopy. 1st column: untreated MDA-MB 231 cells. 2nd column: MDA-MB 231 cells incubated with **1a**. 3rd column: MDA-MB 231 cells treated with **1b**. 4th column: MDA-MB 231 cells incubated with **1c**. Three areas were analyzed per sample. One representative area is depicted. Scale bar = 16 μm.

The fluorescence induced by the complexes depended on their concentration. At 1 μM, only **1c** caused oxidative stress (Figure 5, top row). Cells treated with **1a** and **1b** showed ROS levels comparable to the untreated control. However, at 10 μM (Figure 5, lower row) also **1a** and **1b** enhanced the amounts of free radicals and transformation of the used dye.

#### 2.2.4 Induction of Lipid Peroxidation by Chlorido­[DIPHENYLSALENE]­iron­(III) Complexes

Intracellular ROS can trigger lipid peroxidation, a process, in which free radicals attack lipids - such as polyunsaturated fatty acids (PUFAs) - in various organelles.^38^ Of particular interest is the oxidation of PUFAs in cell membranes, which leads to their destruction. This process called ferroptosis was identified to be part of the mode of action of iron­(III) complexes with Schiff base ligands ([SALENE]­iron­(III) and [SALOPHENE]­iron­(III)).^8–12^

The oxidative damage to lipids can be detected using BODIPY 581/591 C11. When the butadienyl moiety of the polyunsaturated fluorophore in this fat-soluble dye is oxidized by lipid ROS, the emission peak shifts from red (590 nm) to green (510 nm), allowing visualization of lipid oxidation by live confocal microscopy.^39^

MDA-MB 231 cells were treated with **1a**–**c** at concentrations of 1 μM and 10 μM for 24 h. The medium was then replaced with the BODIPY 581/591 C11 working solution (2.5 μM) and incubated for another 20 min.

The image from live confocal microscopy of **1a** at a concentration of 1 μM showed only a slight green coloration, indicating the absence of lipid ROS (Figure 6). At 10 μM, **1a** caused a weak color change from red to green, which is significantly less than would have been expected due to the oxidative cell stress caused by this complex. Images of cells treated with **1b** and **1c** exhibited markedly increased green fluorescence, even at 1 μM. By increasing the concentration to 10 μM, most of the cells were damaged by lipid oxidation.

**Figure 6:**
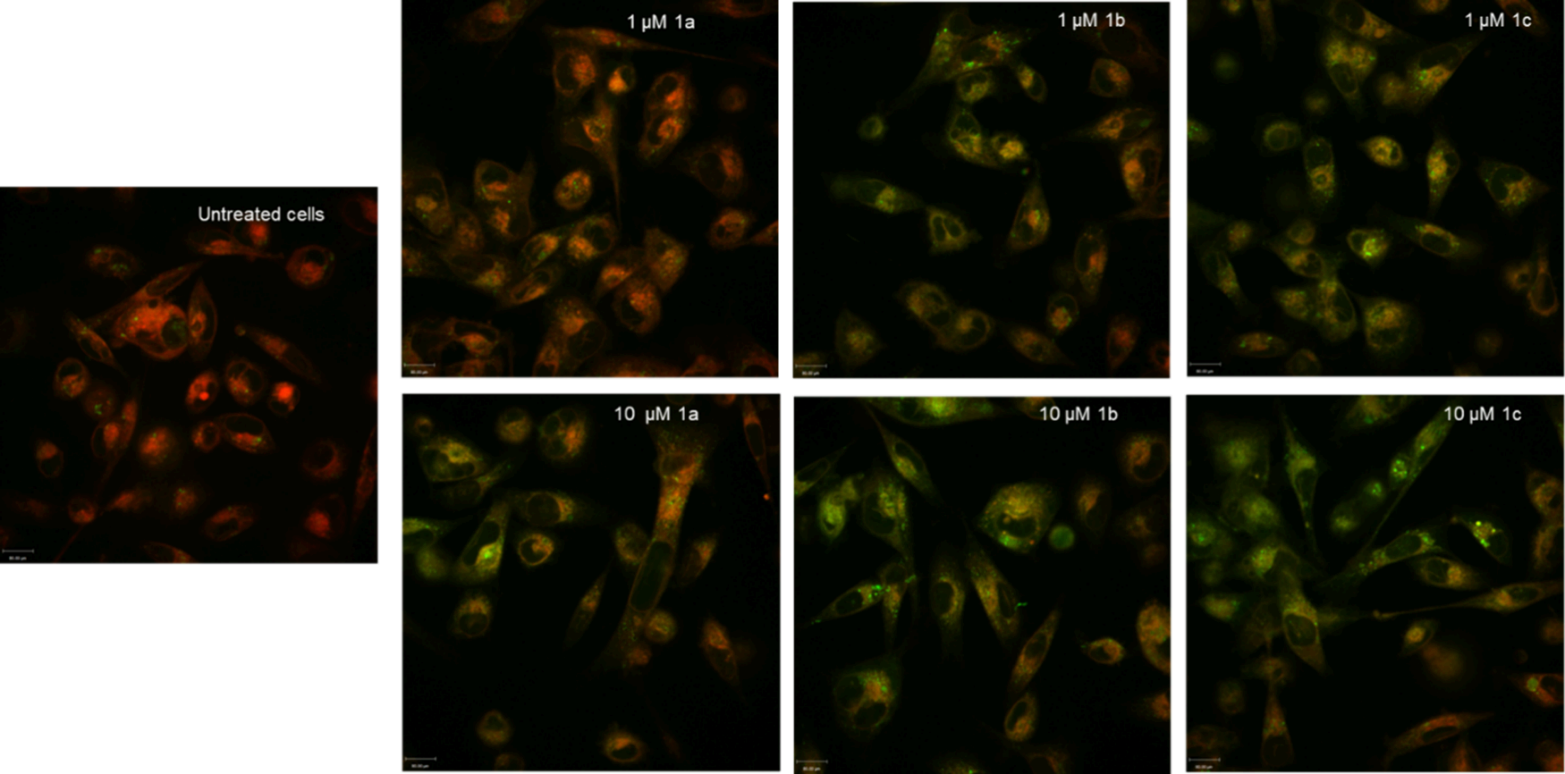
Microscopic observation of lipid peroxidation. MDA-MB 231 cells were treated for 24 h with **1a**–**c** at concentrations of 1 μM and 10 μM, respectively, stained with BODIPY 581/591 C11 and investigated by live confocal microscopy. Three areas were analyzed per sample. One representative example is depicted. Scale bar = 80 μm.

In order to better assess lipid oxidation, the cells were further examined by flow cytometry. MDA-MB 231 cells were incubated for 2, 4, 6, and 24 h with **1a**–**c** at concentrations of 5 μM and 10 μM. Thirty minutes before the end of the incubation period, BODIPY 581/591 C11 was added (concentration: 2.5 μM). Cells were centrifuged, resuspended in PBS and analyzed.

In contrast to other (*RS*)-configured chlorido­[DIARYLSALENE]­iron­(III) complexes (see Scheme 1),^10,12^ no intracellular oxidation of BODIPY 581/591 C11 was observed after incubation with **1a**–**c** for 2, 4, or 6 h (data not shown). Extending the incubation period to 24 h led to the detection of lipid ROS, showing a 2.8 ± 0.2-fold increase at 5 μM and a 3.5 ± 0.2-fold increase at 10 μM by **1a** in comparison to the untreated control. Complexes **1b** and **1c** with ligands in the (*RR/SS*)- or (*SS*)-configuration also induced a delayed effect, but lipid oxidation in the cells was about 4-times higher than with **1a** after 24 h. At a concentration of 5 μM, **1b** induced a slightly higher degree of oxidation than **1c** (11.2 ± 3.1-fold and 8.9 ± 4.6-fold, respectively), but both were comparably active at 10 μM (12.8 ± 5.1-fold and 12.3 ± 6.3-fold, respectively).

#### 2.2.5 Evaluation of Cell Death Mechanisms Induced by Chlorido­[DIPHENYLSALENE]­iron­(III) Complexes

Lipid oxidation, especially the peroxidation of PUFAs, is an important trigger for ferroptosis, a form of iron-dependent cell death. In the Fenton reaction, interaction of iron­(II/III) ions and hydrogen peroxide (H_2_O_2_) produce highly reactive hydroxyl radicals (•OH) that can attack and damage PUFAs in cell membranes, leading to lipid peroxidation and cell death.^40^

Iron­(III)-L complexes with organic ligands (L = any ligand) can also generate ROS through various mechanisms. In a first step, the complexes can be reduced to iron­(II)-L by H_2_O_2_, which, in turn, produces •OH in a Fenton-like reaction.^41^

Furthermore, iron­(III)-L can bind oxygen and, for example, form iron­(III)-L-peroxo complexes, which participate as catalyst precursors in oxidation reactions. Even traces of oxygen induce the formation of μ-oxo-(iron­(III)-L)_2_ complexes, as was demonstrated for **1a** and **2c** in the DMSO stock solution used for cell-culture experiments. Unfortunately, the μ-oxo dimer often represents a catalytically inactive form that is no longer able to react effectively with H_2_O_2_. However, high chloride concentrations, as present in cell-culture medium, led to the formation of monomeric iron species, as demonstrated by HPLC for **1a** (see chapter 2.1), which can participate in the catalytic cycle.^41^

Therefore, a •OH detection test was carried out to investigate whether the iron­(III) complexes described here can be reactivated in the aqueous environment of biological (cellular) systems and then form •OH themselves. The dye used, OH580, is a living cell-permeable probe and can detect •OH quickly and selectively in living cells. In the presence of •OH, a strong red fluorescence is produced in the cells, which can be analyzed qualitatively and quantitatively at λ_ex/em_ = 540/590 nm.

To investigate whether intracellular •OH formation increased after the addition of **1a**–**c**, MDA-MB 231 cells were seeded and incubated for 24 h to allow adhesion. The cells were then stained with OH580 for 1 h and, after a change of medium, treated with **1a**–**c** at concentrations of 1 μM and 10 μM for 4 h. The medium was aspirated, the cells washed with PBS, and assay buffer was added. Additionally, nuclei were stained with Hoechst 33342 and the cell membrane with wheat germ agglutinin (WGA). The cellular staining was immediately analyzed by fluorescence microscopy. In a parallel experiment, the •OH-caused staining was quantified using a microplate reader.

Untreated MDA-MB 231 cells showed no staining, indicating a lack of •OH (Figure 7). In contrast, all complexes induced strong •OH formation in the mitochondria at both tested concentrations. Cell morphology remained unchanged after incubation for 4 h with **1a** and **1b** at 10 μM. However, treatment with **1c** led to loss of cell–cell contacts and rounding of the cells, but not cell death.

**Figure 7:**
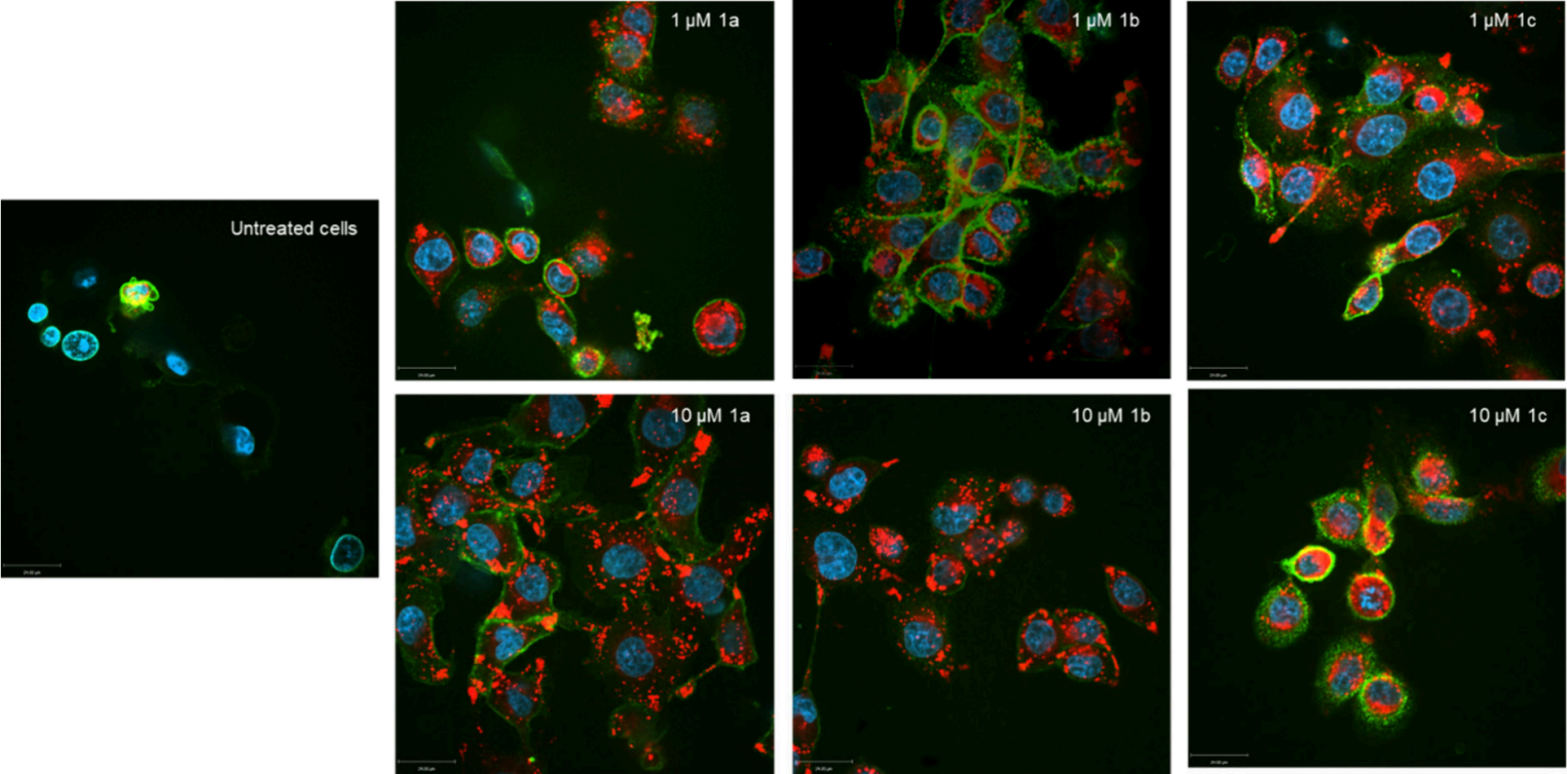
Hydroxyl radical formation in MDA-MB 231 cells analyzed by live-cell microscopy after treatment with **1a**–**c**. The cells were preincubated for 1 h with the OH580 probe followed by an incubation for 4 h with the complexes **1a**–**c** (at 1 μM and 10 μM, respectively). The OH580 probe generated a red fluorescence signal. The nuclei were stained with Hoechst 33342 (blue) and the plasma membranes with wheat germ agglutinin (WGA) (green). At least three areas were analyzed per sample. One representative area is shown. Scale bar = 24 μm.

Quantitative fluorescence analysis revealed a concentration-dependent induction of •OH formation by all complexes. The weakest effects were observed for **1a**. At 1 μM, 1.14 ± 0.05-fold more •OH were formed than in the untreated control (set to 1). Increasing the concentration to 10 μM raised this value to 1.48 ± 0.07. Complexes **1b** and **1c** yielded identical results, with a 1.33 ± 0.12- and 1.32 ± 0.09-fold higher •OH concentration at 1 μM and a 1.60 ± 0.09- and 1.65 ± 0.07-fold higher concentration at 10 μM.

For [SALOPHENE]­iron­(III) complexes, intracellular ROS induction, the formation of lipid ROS, and subsequent ferroptosis have already been described as part of the mode of action.^8,9,11,16,42^ In addition, apoptosis and necroptosis were also identified to cause cell death.

Whether this also applies to the complexes described here was determined by flow cytometry analysis.

Different cell types can be distinguished by staining with annexin V (AnV) and propidium iodide (PI). Annexin V specifically labels apoptotic cells by binding to phosphatidylserine on the outer membrane, while PI is excluded from living cells with an intact membrane, but can enter dead or dying cells and bind to double-stranded DNA there.^43^

Interestingly, AnV can also be used to identify ferroptotic cells, although the exact mechanisms are not yet fully understood. It appears that lipid peroxidation causes membrane changes that allow AnV to bind.^44^ Cells with necroptosis show positive AnV staining, too, as phosphatidylserine externalization also occurs in this programmed cell death.^45^ Therefore, the distinction has to be made indirectly through the use of cell death inhibitors.

In this experiment, MDA-MB 231 cells were incubated with **1a**–**c** alone and in combination with the specific ferroptosis inhibitor ferrostatin-1 (Fer-1)^46^ or the necroptosis inhibitor necrostatin-1 (Nec-1).^47^ Analysis of the stained cells using flow cytometry allows differentiation between living (AnV-/PI-), apoptotic (AnV+/PI-) and nonapoptotic (AnV+/PI+, PI+) cells. A reduction in staining by Fer-1 or Nec-1 indicates the involvement of ferroptosis or necroptosis in cell death.

As only minimal differences between the complexes were detected at 1 μM, concentrations of 5 μM and 10 μM were used and the incubation time was limited to 24 and 48 h to ensure a sufficient number of cells for examination. The inhibitors Fer-1 and Nec-1 were administered at 1 μM and 20 μM, respectively.

Complex **1a** showed in the previous experiments only minor effects also at the highest concentration tested and an incubation period of 48 h. Therefore, **1a** was not investigated further. The complexes **1b** and **1c**, too, only slightly increased apoptotic and nonapoptotic cells after 24 h regardless of the concentration used (). The effects were much more pronounced when the incubation period was extended to 48 h (Figure 8) and are discussed here.

**Figure 8:**
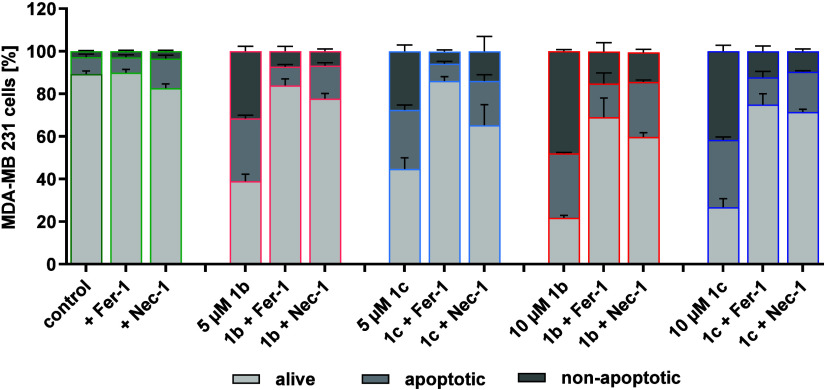
Cell viability and cell death after treatment with **1b** and **1c**. MDA-MB 231 cells were incubated for 48 h with the complexes **1b** or **1c** (at 5 μM and 10 μM, respectively) alone or in combination with the inhibitors Fer-1 (1 μM) or Nec-1 (20 μM). Subsequent annexin V (AnV) and propidium iodide (PI) staining as well as flow cytometry analysis were performed to select alive (AnV-/PI-), apoptotic (AnV+/PI-), and nonapoptotic dead (AnV+/PI+, PI+) cells. Data are presented as mean of 4 experiments + SE.

In the untreated control, the proportion of living cells was 89.3 ± 1.4%, with a small proportion of apoptotic (8.0 ± 1.4%) and nonapoptotic (2.8 ± 0.3%) cells. Addition of Fer-1 alone did not alter these ratios. In contrast, Nec-1 increased the proportion of apoptotic cells (14.0 ± 1.6%) to the detriment of living cells (82.6 ± 2.0%).

Complex **1b** reduced at 5 μM the percentage of viable cells to 39.0 ± 3.3%. This reduction was accompanied by an increase in apoptotic cells (29.5 ± 1.5%) as well as nonapoptotic cells (31.6 ± 2.4%). The simultaneous administration of **1b** and Fer-1 effectively prevented the cell death induced by the complex and reduced the proportion of nonapoptotic (7.3 ± 2.2%) and apoptotic cells (8.9 ± 1.0%), comparable to that found in the Fer-1-treated control. Similarly, Nec-1 reduced the action of **1b**.

Comparable values were found for **1c**, which, however, could not be diminished as effectively by Nec-1 as in the case of **1b**. At a complex concentration of 10 μM, the proportion of nonapoptotic cells increased to 13.9 ± 1.3% (**1b**) and 9.6 ± 1.1% (**1c**). The amount of living cells was reduced in both cases to the same extent. Fer-1 and Nec-1 again counteracted this effect, but not as effective as at 5 μM.

Although the complexes induce mitochondrial •OH formation and lipid ROS, and given the growing evidence of the off-target effect of Nec-1 on ferroptosis^48^ a dual mode of cell death as the mechanism of action of these complexes cannot be completely ruled out. Nevertheless, both necroptosis and ferroptosis play crucial roles in various conditions and diseases,^49–51^ and compounds that target both pathways therefore have significant potential for improving treatment outcomes. However, the effects of this series of complexes, started at a much later time point.

## 3 Conclusions

In this SAR study, antitumor effects of chlorido­[DIPHENYLSALENE]­iron­(III) complexes were evaluated regarding the dependence on the configuration at the 1,2-diphenylethane bridge and the substituents at the salicylidene moieties. For this purpose, complexes with the (*RS*)-, (*RR/SS*)-, and the (*SS*)-configured *N,N′*-disalicylidene-1,2-diphenyl-1,2-diaminoethane ligands were synthesized. Chlorine and bromine substituents were introduced to the salicylidene part at position 5. The effect of disubstitution was studied on the example of the 3-Br, 5-Cl substitution pattern.

The activity of the complexes against MDA-MB 231, A2780 and A2780cis as well as HL-60 cells clearly depended on the configuration. The complexes with the (*RR/SS*)-configured ligand were more potent than with the respective (*RS*)-configured diastereomer. Interestingly, separation of the racemic ligand did not optimize the antiproliferative effects. The complex with (*SS*)-configured ligand showed the same effects as the racemic mixture. Therefore, investigating the complexes with the (*RR*)-configured ligand was not useful and was not carried out.

The 5-Cl and 5-Br substituents at the salicylidene moieties caused comparable effects, while the 3-Br, 5-Cl substitution pattern led to a complete loss of activity. Why the additional 3-Br substituent in **2a**–**c** (→ **3a**–**c**) terminated the cytotoxic activity could not be clarified. The relationship between the biological effects and the stereochemistry of the DIPHENYLSALENE ligands, however, could be demonstrated. This was done on the example of chlorido­[*N,N′*-bis­(5-chlorosalicylidene)-1,2-diphenyl-1,2-diaminoethane]­iron­(III) complexes **1a** (*RS*), **1b** (*RR/SS*), and **1c** (*SS*).

The complexes induced oxidative stress in MDA-MB 231 cells, with this being significantly more pronounced in the case of **1b** and **1c** than in **1a**. The complexes induced in a Fenton-like reaction •OH in the mitochondria as main part of the mitochondrial ROS. Since the complexes are stable in the cell-culture medium, it can be ruled out that they increase the labile iron pool as a trigger for ferroptosis. Nevertheless, lipid oxidation was observed, which was more pronounced in case of complexes with (*RR/SS*)- or (*SS*)-configured ligands. After a 24 h treatment at a concentration of 10 μM, **1b** and **1c** caused a 3.5-fold higher lipid oxidation rate than **1a**, as determined by flow cytometry. A visual examination using confocal microscopy suggests that the differences are even greater. Therefore, it is suggested that the formation of ROS, especially lipid ROS, is the essential part of the mode of action, which explains the lower cytotoxicity of **1a** compared to **1b** and **1c**. In fact, the induction of ferroptosis was demonstrated in MDA-MB 231 for **1b** and **1c**, for which enhancement of lipid ROS was an essential feature.

From this and other test results, it can be concluded that only diastereomers exhibit different effects, but not enantiomers. The lack of difference between enantiomers argues against binding to optically active macromolecules. The variation in the activity between the diastereomers can be explained by their different spatial structures and the binding molecules (e.g., O_2_ or H_2_O_2_) to be activated by the iron­(III) center.

In the less active complexes with (*RS*)-configured ligands, the phenyl rings are oriented at one side of the iron­(III) square planar coordination plane and the fifth coordination site is located on the opposite side. In contrast, the phenyl rings of the iron­(III)-bound (*RR*)- or (*SS*)-configured ligand are exclusively equatorially arranged.

Important binding partner of [DIPHENYLSALENE]­iron­(III) complexes in biological systems are H_2_O_2_ and O_2_. The binding of H_2_O_2_ can lead to the formation of hydroperoxo (FeOOH) intermediates, which can then be involved in further oxidation reactions. The coordination of O_2_ to iron­(III) complexes is also of particular importance. The interaction often leads to the formation of iron­(III)-superoxo (Fe­(O_2_)) or iron­(III)-peroxo (Fe­(O_2_^2–^)) species,^52^ which are precursors for the formation of ROS and subsequent lipid oxidation. Such intermediates are easier to form with complexes bearing DIPHENYLSALENE ligands in (*RR*)- or (*SS*)-configuration, as the metal center is freely accessible due to the equatorially arranged phenyl rings. This spatial structure is thus comparable to [SALOPHENE]­iron­(III) complexes, which have already been identified as effective metal-based antitumor agents. However, these complexes exhibit cellular effects within 1–2 h, whereas [DIPHENYLSALENE]­iron­(III) complexes require 24–48 h to do so.

This clearly shows that DIPHENYLSALENEs give the iron­(III) complexes another pharmacokinetic profile than SALOPHENEs, which probably also affects possible side effects. A further advantage of DIPHENYLSALENE is the possibility of creating a carrier ligand analogous to [1,2-diphenyl-1,2-diaminoethane]­platinum­(II) complexes.^5,53,54^ Substituents at the 1,2-diphenylethane moiety can give the iron­(III) complexes selectivity for different tumors. This will be investigated in detail in the next SAR study.

Finally, it should be mentioned that if DMSO is used to prepare stock solutions for biological testing, the formation of the respective μ-oxo dimer must be considered. However, high chloride concentrations in PBS or cell-culture medium led to cleavage into monomers, which then caused the biological effects.

## 4 Experimental Section

### 4.1 Chemistry

#### 4.1.1 General, Reagents, Devices

Chemical reagents and solvents were supplied from Sigma-Aldrich (St. Louis, MO, USA), Fisher Scientific (Schwerte, Germany), BLD-Pharma (Reinbek, Germany) and were used without further purification, unless otherwise mentioned. All solvents and reagents for HPLC were of LC-grade purity.

Analytical thin-layer chromatography was performed on Polygram SIL G/UV254 (Macherey-Nagel, Düren, Germany) plates (0.25 mm layer thickness) with a fluorescent indicator. The spots were visualized with ultraviolet light at 254/365 nm.

NMR spectra were recorded on a Bruker Ultrashield 400 Plus spectrometer (^1^H NMR, 400 MHz; ^13^C NMR, 100 MHz; Bruker, Billerica, MA, USA). The solvent signal center and the tetramethylsilane signal were used as internal standards. Deuterated solvents were purchased from Eurisotop (Saarbrücken, Germany). Chemical shifts are given in parts per million (ppm) and the coupling constants are displayed in Hertz (Hz).

High-resolution mass spectrometry (HR-MS) was performed on an Orbitrap Elite mass spectrometer (Thermo FisherScientific, Waltham, MA, USA) using direct infusion and heated electrospray ionization. The spectra were analyzed with Xcalibur software.

Elemental analyses were performed with UNICUBE-Elementar (Langensbold, Germany) at the Department of General, Inorganic and Theoretical Chemistry, University of Innsbruck, Austria.

FT-IR spectroscopy was done on a Bruker Alpha spectrometer with an attenuated total reflection unit (Bruker). FT-IR spectra were measured with 32 scans in the wavenumber range covering 4000–400 cm^–1^ and exerting a resolution of 1 cm^–1^.

Optical rotation of the samples was determined using a Jasco P-2000 polarimeter (Jasco, Tokyo, Japan) and ECD spectra of the compounds were measured using a Jasco 1500 circular dichroism spectrophotometer.

EPR spectra were recorded on a Magnettech 5000 X-band spectrometer (Bruker) in a frozen solution of DMSO in 3 mm (outside diameter) fused silica tubes at 98 K.

Crystal structure analysis was performed on a Bruker D8 Quest diffractometer (Bruker) equipped with a Photon III detector using Mo–K_α_ radiation (λ = 71.073 pm).

HPLC measurements were run on a Merck Hitachi Elite LaChrom HPLC system (Hitachi High-Tech Europe, Krefeld, Germany).

[^3^H]-thymidine uptake was determined in a Microbeta Trilux scintillation counter (PerkinElmer, Waltham, USA).

The optical density was measured on an Infinite F50 absorbance plate reader (Tecan, Grödig, Austria).

The scratch assay was performed in the Evident ScanR live-cell imaging system (Evident Europe, Hamburg, Germany).

Live confocal microscopy was done on a Zeiss Axio Observer Z1 instrument (Zeiss, Oberkochen, Germany) in arrangement with a spinning disc confocal system (UltraVIEW VoX, PerkinElmer, Waltham, MA, USA).

Fluorescence was quantitatively determined in a Synergy H1 microplate reader (BioTek, Winooski, VT, USA).

Flow cytometry analysis was performed on FACSCanto II (Becton Dickinson, San Jose, CA, USA).

#### 4.1.2 General Procedure for the Synthesis of the Ligands

A mixture of 1 equivalent (equiv) of 1,2-diphenyl-1,2-diaminoethane and 2 equiv of the appropriately substituted salicylaldehyde was dissolved in anhydrous acetonitrile (MeCN, 10 mL). After stirring for 48 h, the mixture was cooled overnight. The resulting precipitate was collected by filtration and washed twice with ice-cold MeCN. After recrystallization from MeCN, the precipitate was dried in vacuo, and the ligands were obtained as a yellow powder. All ligands were characterized by ^1^H NMR, ^13^C NMR, and FT-IR spectroscopy. The spectra are provided as Supporting Information (). HR-MS confirmed the atomic mass, and CHN analysis approved the elemental composition and purity.

##### 4.1.2.1 (*RS*)-*N*,*N*′-Bis­(5-chlorosalicylidene)-1,2-diphenyl-1,2-diaminoethane (**L1a**)

100 mg (0.471 mmol) of (*RS*)-1,2-diphenyl-1,2-diaminoethane, 149 mg (0.951 mmol) of 5-chlorosalicylaldehyde; yield 186 mg (0.371 mmol, 81%), colorless powder.

^1^H NMR 400 MHz (CDCl_3_): δ 13.04 (s, 2H, OH), 8.00 (s, 2H, N=CH), 7.36–7.18 (m, 12H), 7.03 (d, J = 2.6 Hz, 2H), 6.87 (d, J = 8.8 Hz, 2H), 4.75 (s, 2H, CH).

^13^C NMR 101 MHz (CDCl_3_): δ 164.80 (2C), 159.40 (2C), 139.04 (1C), 132.51 (3C), 130.76 (2C), 128.76 (4C), 128.19 (3C), 127.94 (5C), 123.37 (1C), 119.34 (1C), 118.51 (2C), 79.83 (2C).

FT-IR: v̅ = 3081 w, 1630 s (C=N), 1571 w, 1477 s, 1449 m, 1368 m, 1271 s, 1203 m, 1043 s, 1027 m, 880 w, 697 ss, 457 m.

HR-MS: *m*/*z* [M + H]^+^: calculated: 489.1058, found: 489.1082.

CHN: calculated: C 68.72 H 4.53 N 4.84, found: C 68.69 H 4.61 N 4.73.

##### 4.1.2.2 (*RR*/*SS*)-*N*,*N*′-Bis­(5-chlorosalicylidene)-1,2-diphenyl-1,2-diaminoethane (**L1b**)

99 mg (0.466 mmol) of (*RR*/*SS)*-1,2-diphenyl-1,2-diaminoethane, 148 mg (0.945 mmol) of 5-chlorosalicylaldehyde; yield 179 mg (0.371 mmol, 78%), yellow powder.

^1^H NMR 400 MHz (CDCl_3_): δ 13.24 (s, 2H, OH), 8.19 (s, 2H, N=CH), 7.27–7.13 (m, 12H), 7.10 (d, J = 2.6 Hz, 2H), 6.92 (d, J = 8.8 Hz, 2H), 4.75 (s, 2H, CH).

^13^C NMR 101 MHz (CDCl_3_): δ 165.13 (2C), 159.55 (2C), 138.90 (1C), 132.60 (3C), 130.79 (2C), 128.58 (5C), 127.94 (2C), 127.75 (5C), 123.45 (1C), 119.26 (1C), 118.64 (2C), 80.12 (2C).

FT-IR: v̅ = 3059 w, 1623 s (C=N), 1599 w, 1443 s, 1291 m, 1214 m, 1170 m, 1038 w, 1025 m, 864 m, 700 ss, 511 m.

HR-MS: *m*/*z* [M + H]^+^: calculated: 489.1058, found: 489.1092.

CHN: calculated: C 68.72 H 4.53 N 4.84, found: C 68.71 H 4.61 N 4.68.

##### 4.1.2.3 (*SS*)-*N*,*N*′-Bis­(5-chlorosalicylidene)-1,2-diphenyl-1,2-diaminoethane (**L1c**)

100 mg (0.471 mmol) of (*SS*)-1,2-diphenyl-1,2-diaminoethane, 146 mg (0.932 mmol) of 5-chlorosalicylaldehyde; yield 158 mg (0.322 mmol, 68%), yellow powder.

^1^H NMR 400 MHz (CDCl_3_): δ 13.23 (s, 2H, OH), 8.19 (s, 2H, N=CH), 7.25–7.16 (m, 12H), 7.10 (d, J = 2.6 Hz, 2H), 6.91 (d, J = 8.8 Hz, 2H), 4.75 (s, 2H, CH).

^13^C NMR 101 MHz (CDCl_3_): δ 165.13 (2C), 159.55 (2C), 138.89 (1C), 132.60 (3C), 130.79 (2C), 128.58 (5C), 127.94 (2C), 127.75 (5C), 123.45 (1C), 119.26 (1C), 118.64 (2C), 80.11 (2C).

FT-IR: v̅ = 3060 w, 1626 s (C=N), 1475 s, 1452 m, 1370 m, 1271 s, 1201 w, 1178 m, 811 m, 770m, 695 ss, 459 m.

HR-MS: *m*/*z* [M + H]^+^: calculated: 489.1058, found: 489.1095.

CHN: calculated: C 68.72 H 4.53 N 4.84, found: C 68.50 H 4.60 N 5.12.

[α]_*D*_^25^ = −82.2 (*c* 0.069, MeOH), ECD (c = 0.74 mM, MeOH): λ (mdeg) 207 (−3.24), 219.5 (10.49), 233 (−5.51), 253 (−2.87), 287 (+1.6), 340 (+1.57).

##### 4.1.2.4 (*RS*)-*N*,*N*′-Bis­(5-bromosalicylidene)-1,2-diphenyl-1,2-diaminoethane (**L2a**)

102 mg (0.480 mmol) of (*RS*)-1,2-diphenyl-1,2-diaminoethane, 195 mg (0.970 mmol) of 5-bromosalicylaldehyde; yield 66 mg (0.113 mmol, 56%), yellow powder.

^1^H NMR 400 MHz (CDCl_3_): δ 13.07 (s, 2H, OH), 7.99 (s, 2H, N=CH), 7.38–7.21 (m, 12H) 7.17 (d, J = 2.5 Hz, 2H), 6.83 (d, J = 8.8 Hz, 2H), 4.75 (s, 2H, CH).

^13^C NMR 101 MHz (CDCl_3_): δ 164.70 (3C), 159.88 (1C), 139.03 (1C), 135.31 (2C), 133.74 (3C), 128.78 (5C), 128.21 (2C), 127.93 (4C), 119.97 (1C), 118.96 (3C), 110.22 (1C), 79.80 (2C).

FT-IR: v̅ = 3080 w, 1631 s (C=N), 1571 w, 1475 s, 1270 s, 1203 w, 1178 m, 1042 s, 1027 m, 821m, 756 s, 696 s, 492 w, 456 m.

HR-MS: *m*/*z* [M + H]^+^: calculated: 577.0048, found: 577.0035.

CHN: calculated: C 58.15 H 3.83 N 4.84, found: C 58.02 H 3.94 N 4.80.

##### 4.1.2.5 (*RR*/*SS*)-*N*,*N*′-Bis­(5-bromosalicylidene)-1,2-diphenyl-1,2-diaminoethane (**L2b**)

103 mg (0.485 mmol) of (*RR*/*SS*)-1,2-diphenyl-1,2-diaminoethane, 195 mg (0.970 mmol) of 5-bromosalicylaldehyde; yield 248 mg (0.428 mmol, 88%), yellow powder.

^1^H NMR 400 MHz (CDCl_3_): δ 13.27 (s, 2H, OH), 8.18 (s, 2H, N=CH), 7.36 (dd, J = 8.8, 2.4 Hz, 2H), 7.27–7.13 (m, 12H), 6.8 (d, J = 8.8 Hz, 2H), 4.75 (s, 2H, CH).

^13^C NMR 101 MHz (CDCl_3_): δ 165.05 (3C), 160.03 (1C), 138.88 (1C), 135.41 (2C), 133.78 (2C), 128.58 (5C), 127.95 (2C), 127.74 (4C), 119.89 (2C), 119.08 (3C), 110.32 (1C), 80.10 (2C).

FT-IR: v̅ = 3059 w, 1625 s (C=N), 1569 w, 1472 s, 1451 m, 1270 s, 1199 w, 1177 m, 1073 w, 816 m, 768 m, 694 s, 457 m.

HR-MS: *m*/*z* [M + H]^+^: calculated: 577.0048, found: 577.0037.

CHN: calculated: C 58.15 H 3.83 N 4.84, found: C 57.91 H 3.91 N 4.74.

##### 4.1.2.6 (*SS*)-*N,N′*-Bis­(5-bromosalicylidene)-1,2-diphenyl-1,2-diaminoethane (**L2c**)

100 mg (0.471 mmol) of (*SS*)-1,2-diphenyl-1,2-diaminoethane, 191 mg (0.950 mmol) of 5-bromosalicylaldehyde; yield 206 mg (0.418 mmol, 73%), yellow powder.

^1^H NMR 400 MHz (DMSO): δ 13.27 (s, 2H, OH), 8.50 (s, 2H, N=CH), 7.46 (dd, J = 8.8, 2.5 Hz, 2H), 7.32–7.16 (m, 12H), 6.87 (d, J = 8.8 Hz, 2H), 5.09 (s, 2H, CH).

^13^C NMR 101 MHz (CDCl_3_): δ 165.05 (3C), 160.03 (1C), 138.87 (1C), 135.41 (2C), 133.78 (2C), 128.58 (5C), 127.95 (2C), 127.74 (4C), 119.89 (2C), 119.09 (3C), 110.32 (1C), 80.09 (2C).

FT-IR: v̅ = 3060 w, 1625 s (C=N), 1569 m, 1472 s, 1452 w, 1368 m, 1271 s, 1199 w, 1177 m, 1026 m, 816 s, 696 s, 457 m.

HR-MS: *m*/*z* [M + H]^+^: calculated: 577.0048, found: 577.0052.

CHN: calculated: C 58.15 H 3.83 N 4.84, found: C 57.98 H 3.92 N 4.54.

[α]_*D*_^25^ = −61.6 (*c* 0.095, MeOH), ECD (c = 0.64 mM, MeOH): λ (mdeg) 206.2 (−3.04), 220 (17.85), 235.3 (−20.65), 250 (−10.83), 280 (+2.33), 341 (+4.42).

##### 4.1.2.7 (*RS*)-*N*,*N*′-Bis­(3-bromo-5-chlorosalicylidene)-1,2-diphenyl-1,2-diaminoethane (**L3a**)

98 mg (0.461 mmol) of (*RS*)-1,2-diphenyl-1,2-diaminoethane, 217 mg (0.921 mmol) of 3-bromo-5-chlorosalicylaldehyde; yield 262 mg (0.404 mmol, 88%), yellow powder.

^1^H NMR 400 MHz (CDCl_3_): δ 14.04 (s, 2H, OH), 7.88 (s, 2H, N=CH), 7.53 (d, J = 2.5 Hz, 2H), 7.37–7.28 (m, 12H), 4.73 (s, 2H, CH).

^13^C NMR 101 MHz (CDCl_3_): δ 164.37 (3C), 156.56 (1C), 135.34 (3C), 130.08 (2C), 129.02 (6C), 128.47 (3C), 127.74 (4C), 123.63 (2C), 119.37 (2C), 79.85 (2C).

FT-IR: v̅ = 3072 w, 1626 s (C=N), 1437 s, 1291 m, 1270 m, 1168 s, 1044 m, 804 m, 766 s, 736 w, 701 ss, 576 m, 510 m.

HR-MS: *m*/*z* [M + H]^+^: calculated: 644.9268, found: 644.9310.

CHN: calculated: C 51.96 H 3.12 N 4.33, found: C 51.88 H 3.18 N 4.37.

##### 4.1.2.8 (*RR/SS*)-*N*,*N*′-Bis­(3-bromo-5-chlorosalicylidene)-1,2-diphenyl-1,2-diaminoethane (**L3b**)

101 mg (0.475 mmol) of (*RR*/*SS*)-1,2-diphenyl-1,2-diaminoethane, 226 mg (0.959 mmol) of 3-bromo-5-chlorosalicylaldehyde; yield 256 mg (0.395 mmol, 83%), yellow powder.

^1^H NMR 400 MHz (CDCl_3_): δ 14.14 (s, 2H, OH), 8.25 (s, 2H, N=CH), 7.54 (d, J = 2.4 Hz, 2H), 7.26–7.17 (m, 12H), 4.75 (s, 2H, CH).

^13^C NMR 101 MHz (CDCl_3_): δ 164.61 (3C), 156.61 (1C), 138.04 (1C), 135.47 (2C), 130.25 (2C), 128.71 (5C), 128.19 (3C), 127.75 (5C), 123.84 (1C), 119.31 (1C), 111.47 (2C), 79.98 (2C).

FT-IR: v̅ = 3059 w, 1623 s (C=N), 1599 w, 1443 s, 1368 m, 1291 m, 1214 m, 1170 s, 1039 m, 864 s, 775 m, 700 ss, 511 m.

HR-MS: *m*/*z* [M + H]^+^: calculated: 644.9268, found: 644.9317.

CHN: calculated: C 51.96 H 3.12 N 4.33, found: C 52.07 H 3.19 N 4.34.

##### 4.1.2.9 (*SS*)-*N,N′*-Bis­(3-bromo-5-chlorosalicylidene)-1,2-diphenyl-1,2-diaminoethane (**L3c**)

101 mg (0.475 mmol) of (*SS*)-1,2-diphenyl-1,2-diaminoethane, 228 mg (0.968 mmol) of 3-bromo-5-chlorosalicylaldehyde; yield 221 mg (0.341 mmol, 71%), yellow powder.

^1^H NMR 400 MHz (CDCl_3_): δ 14.14 (s, 2H, OH), 8.25 (s, 2H, N=CH), 7.54 (d, J = 2.5 Hz, 2H), 7.27–7.10 (m, 12H), 4.76 (s, 2H, CH).

^13^C NMR 101 MHz (CDCl_3_): δ 164.60 (3C), 156.61 (2C), 138.04 (1C), 135.47 (2C), 130.25 (2C), 128.71 (5C), 128.19 (3C), 127.75 (4C), 123.84 (1C), 119.31 (1C), 111.47 (2C), 79.98 (2C).

FT-IR v̅ = 3067 w, 1620 s (C=N), 1443 s, 1370 m, 1292 m, 1169 s, 1059 m, 1026 m, 772 m, 695 ss, 722 m, 506 m.

HR-MS: *m*/*z* [M + H]^+^: calculated: 644.9268, found: 644.9282.

CHN: calculated: C 51.96 H 3.12 N 4.33, found: C 52.06 H 3.20 N 4.39.

[α]_*D*_^25^ = −68.3 (*c* 0.069, MeOH), ECD (c = 0.61 mM, MeOH): λ (mdeg) 215 (−9.22), 230 (+6.78), 295 (+2.05), 406 (−2.86).

#### 4.1.3 General Procedure for the Synthesis of the Chlorido­[DIPHENYLSALENE]­iron­(III) Complexes

Briefly, a mixture of 1 equiv of the ligand and 1 equiv of the anhydrous iron­(III) chloride was dissolved in anhydrous ethanol (7 mL) to form the respective iron­(III) complexes. After stirring under reflux for 2 h, the precipitate was isolated and recrystallized from anhydrous ethanol and dried in vacuo. All synthesized complexes were characterized by FT-IR and EPR spectroscopy. The spectra are provided as Supporting Information (). The atomic mass was confirmed by HR-MS. Elemental analysis confirmed the elemental composition. All compounds showed a purity >95%, as determined by HPLC analysis.

##### Chlorido­[(*RS*)-*N,N′*-bis­(5-chlorosalicylidene)-1,2-diphenyl-1,2-diaminoethane]­iron­(III) (**1a**)

100 mg (0.204 mmol) of **L1a**, 33 mg (0.204 mmol) of iron­(III) chloride; yield 53 mg (0.088 mmol, 43%), dark powder.

FT-IR: v̅ = 1735 w, 1630 s, 1608 s, 1525 s (N=C), 1373 m, 1245 m, 1201 m, 1177 m (C–O), 828 s, 798 s, 659 s, 545 m, 484 m.

HR-MS: *m*/*z* (M-Cl): calculated: 542.0251, found: 542.0253.

CHN: calculated: C 58.12 H 3.48 N 4.84, found: C 57.71 H 3.53 N 4.74.

EPR: (9.5 GHz, 98 K) g_⊥_ = 4.16, g_II_ = 8.00.

[α]_*D*_^25^ = −616.8 (*c* 0.026, MeOH), ECD (c = 0.46 mM, MeOH): λ (mdeg) 209 (−12.94), 247 (+11.09), 269 (+10.26), 305 (+3.6), 367 (+1.75), 341 (+4.42).

##### Chlorido­[(*RR/SS*)-*N,N′*-bis­(5-chlorosalicylidene)-1,2-diphenyl-1,2-diaminoethane]­iron­(III) (**1b**)

102 mg (0.208 mmol) of **L1b**, 33.7 mg (0.208 mmol) of iron­(III) chloride; yield 82 mg (0.136 mmol, 65%), dark powder.

FT-IR: v̅ = 1739 w, 1612 s, 1530 m (N=C), 1373 m, 1293 m, 1200 w, 1180 s (C–O), 827 s, 779 s, 665 ss, 523 s, 454 s.

HR-MS: *m*/*z* (M-Cl): calculated: 542.0251, found: 542.0258.

CHN: calculated: C 58.12 H 3.48 N 4.84, found: C 57.96 H 3.51 N 4.66.

EPR: (9.5 GHz, 98 K) g_⊥_ = 4.01, g_II_ = 7.69.

##### Chlorido­[(*SS*)-*N,N′*-bis­(5-chlorosalicylidene)-1,2-diphenyl-1,2-diaminoethane]­iron­(III) (**1c**)

100 mg (0.204 mmol) of **L1c**, 33 mg (0.204 mmol) of iron­(III) chloride; yield 96 mg (0.159 mmol, 78%), dark powder.

FT-IR: v̅ = 2173 w, 1612 s, 1530 m (N=C), 1372 m, 1292 m, 1276 m, 1178 s (C–O), 826 s, 778 s, 664 ss, 526 s, 454 s.

HR-MS: *m*/*z* (M-Cl): calculated: 542.0251, found: 542.0258.

CHN: calculated: C 58.12 H 3.48 N 4.84, found: C 57.90 H 3.60 N 4.72.

EPR: (9.5 GHz, 98 K) g_⊥_ = 4.10, g_II_ = 7.71.

[α]_*D*_^25^= −584.4 (*c* 0.024, MeOH), ECD (c = 0.68 mM, MeOH): λ (mdeg) 210 (−18.54), 247 (+16.75), 269 (+18.35), 308 (+5.95), 371 (+3.52).

##### Chlorido­[(*RS*)-*N,N′*-bis­(5-bromosalicylidene)-1,2-diphenyl-1,2-diaminoethane]­iron­(III) (**2a**)

100 mg (0.172 mmol) of **L2a**, 27.9 mg (0. 172 mmol) of iron­(III) chloride; yield 62 mg (0.090 mmol, 52%), dark powder.

FT-IR: v̅ = 3855 w, 1606 s, 1523 m (N=C), 1371 m, 1351 w, 1289 s, 1199 w, 1178 m (C–O), 827 s, 781m, 649 ss, 521 s, 441 s.

HR-MS: *m*/*z* (M-Cl): calculated: 629.9241, found: 629.9261.

CHN: calculated: C 50.38 H 3.02 N 4.20, found: C 50.62 H 3.13 N 4.17.

EPR: (9.5 GHz, 98 K) g_⊥_ = 4.18, g_II_ = 8.15.

##### Chlorido­[(*RR/SS*)-*N,N′*-bis­(5-bromosalicylidene)-1,2-diphenyl-1,2-diaminoethane]­iron­(III) (**2b**)

101 mg (0.174 mmol) of **L2b**, 28.2 mg (0.174 mmol) of iron­(III) chloride; yield 85 mg (0.123 mmol, 71%), dark powder.

FT-IR: v̅ = 3855 w, 1610 s, 1525 m (N=C), 1370 m, 1292 s, 1200 w, 1179 m (C–O), 827 s, 775 m, 694 s, 652 ss, 523 s, 448 s.

HR-MS: *m*/*z* (M-Cl): calculated: 629.9241, found: 629.9233.

CHN: calculated: C 50.38 H 3.02 N 4.20, found: C 50.41 H 3.14 N 4.13.

EPR: (9.5 GHz, 98 K) g_⊥_ = 4.14, g_II_ = 7.75.

##### Chlorido­[(*SS*)-*N,N′*-bis­(5-bromosalicylidene)-1,2-diphenyl-1,2-diaminoethane]­iron­(III) (**2c**)

100 mg (0.172 mmol) of **L2c**, 27.9 mg (0.172 mmol) of iron­(III) chloride; yield 76 mg (0.110 mmol, 64%), dark powder.

FT-IR: v̅ = 3032 w, 1611 s, 1526 m (N=C), 1370 m, 1290 m, 1199 w, 1177 m (C–O), 825 s, 773 m, 651 ss, 522 s, 448 s.

HR-MS: *m*/*z* (M-Cl): calculated: 629.9241, found: 629.9241.

CHN: calculated: C 50.38 H 3.02 N 4.20, found: C 49.98 H 2.96 N 3.82.

EPR: (9.5 GHz, 98 K) g_⊥_ = 4.10, g_II_ = 7.56.

##### Chlorido­[(*RS*)-*N,N′*-bis­(3-bromo-5-chlorosalicylidene)-1,2-diphenyl-1,2-diaminoethane]­iron­(III) (**3a**)

100 mg (0.154 mmol) of **L3a**, 33 mg (0.154 mmol) of iron­(III) chloride; yield 98 mg (0.126 mmol, 82%), dark powder.

FT-IR: v̅ = 3064 w, 1598 s, 1512 m (N=C), 1421 s, 1377 w, 1293 m, 1211 m, 1167 s (C–O), 855 s, 751 s, 711 s, 479 s.

HR-MS: *m*/*z* (M-Cl): calculated: 697.8461, found: 697.8486.

CHN: calculated: C 45.67 H 2.46 N 3.80, found: C 45.39 H 2.82 N 3.92.

EPR: (9.5 GHz, 98 K) g_⊥_ = 4.15, g_II_ = 7.93.

##### Chlorido­[(*RR/SS*)-*N,N′*-bis­(3-bromo-5-chlorosalicylidene)-1,2-diphenyl-1,2-diaminoethane]­iron­(III) (**3b**)

100 mg (0.154 mmol) of **L3b**, 25 mg (0.154 mmol) of iron­(III) chloride; yield 91 mg (0.117 mmol, 76%), dark powder.

FT-IR: v̅ = 3059 w, 1609 s, 1516 m (N=C), 1423 s, 1375 w, 1297 m, 1209 m, 1162 s (C–O), 857 m, 745 ss, 693 s, 477 s.

HR-MS: *m*/*z* (M-Cl): calculated: 697.8461, found: 697.8494.

CHN: calculated: C 45.67 H 2.46 N 3.80, found: C 45.90 H 2.52 N 3.47.

EPR: (9.5 GHz, 98 K) g_⊥_ = 4.14, g_II_ = 7.60.

##### Chlorido­[(*SS*)-*N,N′*-bis­(3-bromo-5-chlorosalicylidene)-1,2-diphenyl-1,2-diaminoethane]­iron­(III) (**3c**)

101 mg (0.156 mmol) of **L3c**, 25.3 mg (0.156 mmol) of iron­(III) chloride; yield 101 mg (0.130 mmol, 83%), dark powder.

FT-IR: v̅ = 3062 w, 1596 s, 1519 m (N=C), 1422 s, 1372 w, 1210 m, 1170 s (C–O), 861 m, 749 ss, 692 m, 541 s, 484 s.

HR-MS: *m*/*z* (M-Cl): calculated: 697.8461, found: 697.8502.

CHN: calculated: C 45.67 H 2.46 N 3.80, found: C 45.27 H 2.52 N 3.72.

EPR: (9.5 GHz, 98 K) g_⊥_ = 4.08, g_II_ = 7.51.

[α]_*D*_^25^ = −516.1 (*c* 0.032, MeOH), ECD (c = 0.44 mM, MeOH): λ (mdeg) 206 (−7.86), 255 (+6.5), 274 (+9.38), 309 (+3.16), 376 (+1.52).

#### 4.1.4 Calculation of ECD Spectrum of **L3c**

Conformational analysis of **L3c** was performed with MacroModel 9.1 (Schrödinger. LLC, New York, NY, USA), using OPLS-3 as the force field, and in the gas phase. Geometrical optimization and energy calculations of conformers occurring in the energy window of 5 kcal/mol were carried out at DFT/6-31G­(d,p) level in the gas phase using Gaussian 16.^55^ No imaginary frequencies were observed. Subsequently, the conformers with population over 3% (Scheme 3B) were subjected to ECD calculation at TD-DFT/B3LYP/6-31G­(d,p)/CPCM in MeOH. ECD spectra obtained (with a half-band of 0.4 eV) were Boltzmann-averaged and compared with the experimental spectrum of **L3c** obtained in MeOH using SpecDis v. 1.71.^23^ No UV shift was applied.

#### 4.1.5 HPLC Analyses

HPLC analyses were conducted as previously described,^9^ with some modifications. In brief, measurements were performed using a Merck Hitachi Elite LaChrom HPLC system (Hitachi High-Tech Europe) equipped with an L-2130 pump, L-2200 autosampler, L-2450 diode array detector (DAD), and L-2480 fluorescence detector. Chromatographic separation was achieved on a LiCrospher 100 RP-18 (5 μm) column, preceded by a Phenomenex SecurityGuard C18 column protection cartridge, maintained at 40 °C. Complexes **1a**–**3c** were solved in methanol and a sample volume of 20 μL was injected for each run. Elution was carried out at a constant flow rate of 1.0 mL min^–1^. The mobile phase consisted of an isocratic mixture of 40% (v/v) methanol and 60% (v/v) phosphate buffer (25 mM potassium dihydrogen phosphate), adjusted to pH 3.0 with phosphoric acid. Detection was performed at 220 nm. Data acquisition and processing were conducted using EZChrom Elite software (Agilent, Santa Clara, CA, USA).

The HPLC retention times and corresponding peak area percentages for complexes **1a**–**3c** are summarized in , .

#### 4.1.6 Crystallographic Analysis

The crystals of the complexes **1a**–**c**, **2a**, and **3c** were measured at a steady temperature of 153 K on a Bruker D8 Quest diffractometer equipped with a Photon III detector using Mo–K_α_ radiation (λ = 71.073 pm). A multiscan absorption correction was performed for all compounds. Using Olex2 ^56^ as a graphical interface, the structures were solved with ShelXT^57^ using dual methods. The structure models were refined with ShelXL^58^ using full matrix least-squares minimization on F^2^. All non-hydrogen atoms were refined anisotropically. The hydrogen atom positions were calculated geometrically and refined using the riding model. The Figures depicting the molecular structures were generated using Olex2.^56^

### 4.2 Biological Assays

#### 4.2.1 Cell-Culture

The ovarian carcinoma cell lines A2780 and A2780cis were kindly provided by the Department of Gynecology, Medical University of Innsbruck, Innsbruck, Austria. The mammary carcinoma cell line MDA-MB 231 and the acute myeloid leukemia cell line HL-60 were purchased from DSMZ-German Collection of Microorganisms and Cell Cultures, (Braunschweig, Germany). The nonmalignant stroma cell line HS-5 was obtained from American Type Culture Collection (ATCC; Manassas, VA, USA). Cell-culture medium of all cell lines consisted of RPMI 1640 medium without phenol red (Pan-Biotech, Aidenbach, Germany), supplemented with l-glutamine (2 mM), penicillin (100 U/mL), and streptomycin (100 μg/mL) (all from Sigma-Aldrich, St. Louis, MO, USA), and 10% fetal bovine serum (FBS; Pan-Biotech). Cells were cultured at 37 °C under a humidified atmosphere of 5% CO_2_ and 95% air and passaged and fed twice weekly. To maintain Cisplatin resistance in A2780cis cells, the cells were incubated biweekly with Cisplatin at a concentration of 1 μM. Cisplatin was dissolved in dimethylformamide at a concentration of 10 mM and stored at −20 °C. The iron­(III) complexes were dissolved in DMSO at a concentration of 10 mM and stored at −20 °C. Upon dilution with cell-culture medium without FBS, the final test concentrations were achieved. Fer-1 and Nec-1 were purchased from Sigma-Aldrich. Both compounds were dissolved in DMSO and further diluted with cell-culture medium to reach a final concentration of 1 μM and 20 μM shortly before addition to the cells.

#### 4.2.2 Analysis of Proliferation and Metabolic Activity

MDA-MB 231, A2780, A2780cis, and HS-5 cells were seeded in triplicate in flat-bottomed 96-well plates (Falcon; Corning Science, Tamaulipas, Mexico) at a density of 1 × 10^4^ cells in 100 μL of cell-culture medium per well and incubated at 37 °C in a humidified atmosphere of 5% CO_2_ and 95% air for 24 h. Exponentially growing HL-60 cells were also seeded in triplicate into U-bottomed 96-well plates (Falcon) at a density of 2 × 10^4^ cells in 100 μL of cell-culture medium per well and incubated for 2 h. Subsequently, complexes were added to achieve the final concentrations in a total volume of 150 μL, and the cells were incubated for 72 h. During the last 16–18 h of the incubation period, 2 μCi [^3^H]-thymidine (2 Ci/mmol, Hartmann Analytic, Braunschweig, Germany) in 50 μL of cell-culture medium were added to each well. Cells were harvested using a semiautomated device, and [^3^H]-thymidine uptake, expressed in cpm, was measured using a scintillation counter. Proliferation in the absence of complexes was set to 100%, and the efficacy of the complexes to inhibit proliferation was calculated as the percentage relative to the cell control without complex treatment.

For the determination of metabolic activity, cells were cultured and treated with the complexes as described above. After 72 h, metabolic activity was assessed using the modified 3-(4,5-dimethylthiazol-2-yl)-2,5-diphenyltetrazolium bromide (MTT) assay (EZ4U kit; Biomedica, Vienna, Austria), according to the manufacturer’s instructions. After subtracting the absorbance of medium supplemented with 10% FBS from all values to account for nonspecific staining, the results were normalized to the untreated control, which was set to 100%.

#### 4.2.3 Scratch Assay

MDA-MB 231 cells (2 × 10^5^ cells/well) were seeded in 200 μL cell-culture medium into a μ-slide 8-well slide (Ibidi, Munich, Germany) and incubated for 24 h at 37 °C in a humidified atmosphere of 5% CO_2_ and 95% air. After reaching at least 90% confluence, a scratch was made with a 200 μL pipet tip into the cell layer. Subsequently, the complexes were added at a concentration of 1 μM and 10 μM and incubated for further 72 h at 37 °C in the Evident ScanR live-cell imaging system (Evident Europe Hamburg, Germany). Every 2 h pictures were taken to document the closing of the scratch and the morphology of the cells. The images were analyzed using Olympus CellSENS Dimension Desktop 4.4.1. software (Evident).

#### 4.2.4 Detection of Oxidative Stress

MDA-MB 231 cells (4 × 10^4^ cells/well) were seeded in 200 μL of cell-culture medium into a μ-slide 8-well slide and incubated for 24 h in a humidified atmosphere of 5% CO_2_ and 95% air. Subsequently, cells were treated with the complexes at concentrations of 1 μM and 10 μM and incubated at 37 °C. After 24 h of incubation, 2-(4-(2-hydroxyethyl)­piperazinyl)­ethanesulfonic acid (HEPES, Biochrom, Berlin, Germany) at a final concentration of 20 μM and MitoTracker Red CM-H_2_XRos (Invitrogen; Life Technologies Corporation, Eugene, Oregon, USA) at a final concentration of 200 nM were added to each well and incubated for further 15 min at 37 °C. Immediately thereafter, samples were analyzed by live confocal microscopy.

#### 4.2.5 BODIPY 581/591 C11 Staining for Examination of Lipid Peroxidation

For flow cytometry, 1 × 10^5^ MDA-MB 231 cells/well were seeded in 2 mL of cell-culture medium into 24-well plates (Greiner bio-one, Frickenhausen, Germany) and incubated for 24 h in a humidified atmosphere of 5% CO_2_ and 95% air. Complexes were added at concentrations of 5 μM and 10 μM, and cells were further treated for 2, 4, 6, and 24 h. Subsequently, the cells were detached with accutase (Sigma-Aldrich) and the cells were transferred into 5 mL round-bottom tubes (Falcon), washed with PBS, and resuspended in 100 μL of BODIPY 581/591 C11 (final concentration: 2.5 μM; Invitrogen). Cells were incubated in the dark for 30 min at 37 °C. Finally, cells were centrifuged (200g, 5 min, 4 °C), resuspended in PBS, and kept on ice. Flow cytometric analysis was performed immediately. Lipid peroxidation in the absence of the complexes was set to 1, and the efficacy of the complexes to induce lipid peroxidation was calculated as a fold increase to the cell control without complex treatment.

For live confocal microscopy, MDA-MB 231 cells were seeded in 200 μL cell-culture medium into μ-slide 8-well slides (4 × 10^4^ cells/well) and incubated for 24 h in a humidified atmosphere of 5% CO_2_ and 95% air. Thereafter, cells were treated with complexes **1a**–**c** at concentrations of 5 μM and 10 μM, respectively, for further 24 h. At the end of the incubation, the cell-culture medium was replaced with 200 μL of BODIPY 581/591 C11 (final concentration: 2.5 μM; Invitrogen) and 5 μL of HEPES (final concentration: 20 μM). Live confocal microscopy was performed after 20 min of incubation in the dark.

#### 4.2.6 Evaluation of the Formation of Hydroxyl Radicals

The mitochondrial •OH radical detection kit (Abcam, Cambridge, UK) was used for the fluorescence detection by fluorescence microscopy as well as by a microplate reader according to the manufacturer’s instructions. In brief, MDA-MB 231 cells were seeded for fluorescence microscopy in 200 μL of cell-culture medium into μ-slide 8-well slides (2 × 10^4^ cells/well) and incubated for 24 h in a humidified atmosphere of 5% CO_2_ and 95% air. Thereafter, cells were stained with OH580 for 1 h and treated with **1a**–**c** at concentrations of 1 μM and 10 μM for 4 h. Thereafter, the cell-culture medium was aspirated, the cells were washed with PBS, and assay buffer was added. The nuclei were stained with Hoechst 33342 (Sigma-Aldrich) and the cell membrane with WGA (Alexa Fluor 647 conjugate, Molecular Probes, Invitrogen, Paisley, UK).

For quantifying the formation of the mitochondrial •OH radicals MDA-MB 231 cells were seeded in 100 μL cell-culture medium into black 96-well plates with transparent bottom (Greiner bio-one; 4 × 10^4^ cells/well) and incubated for 24 h in a humidified atmosphere of 5% CO_2_ and 95% air. Staining and treatment with **1a**–**c** was performed as described above. The cell-culture medium was replaced with 100 μL of assay buffer solution and •OH radical formation was immediately analyzed at λ_ex/em_ = 540/590 nm on the Synergy H1 microplate reader (BioTek) with bottom read mode.

#### 4.2.7 Flow Cytometric Analysis of Cell Viability and Cell Death

MDA-MB 231 cells were seeded in 2 mL cell-culture medium into a 24-well plate (0.1 × 10^6^ cells/well) and were incubated for 24 h in a humidified atmosphere of 5% CO_2_ and 95% air. Thereafter, either Fer-1 (final concentration: 1 μM) or Nec-1 (final concentration: 20 μM) were added to the respective wells shortly before the addition of the complexes **1b** or **1c** at the concentrations of 5 μM or 10 μM. Appropriate negative controls, consisting of cells without complex addition or with the addition of only the cell death inhibitors, were also prepared. Cells were incubated for 24 and 48 h in a humidified atmosphere of 5% CO_2_ and 95% air. After incubation, the cells were detached with accutase and the supernatant and cells were transferred into 5 mL round-bottom tubes, washed with 1× AnV buffer (3 min, 200g, rt), and double-stained in 50 μL of 1× AnV buffer with 1 μL of AnV (MabTag GmbH, Friesoythe, Germany) conjugated to fluorescein isothiocyanate (FITC) dye (green fluorescence) and 1 μL of PI (Sigma-Aldrich), which allows discrimination between viable (AnV–/PI−), apoptotic (AnV+/PI−), and nonapoptotic (AnV+/PI+, PI+) dead cells. Following incubation for 20 min at 4 °C in the dark, flow cytometric analysis was performed.

#### 4.2.8 Statistical Analysis

The Wilcoxon test for paired groups was applied to evaluate differences in proliferation and metabolic activity between incubation without and with varying concentrations of the complexes (NCSS software 7.1.2, Kaysville, UT, USA). A p-value <0.05 was considered statistically significant. IC_50_ values were calculated and Figures were generated using GraphPad Prism 10.1.2.

## Supplementary Material





## Notes

^1^H, ^13^C NMR and FT-IR spectra of **L1a**-**L3c**. FT-IR spectra of **1a**–**3c**. EPR spectra of **1a**–**3c**. HPLC spectra of **1a**–**3c**. ECD spectra of (*SS)*- and (*RR*/*SS)*-1,2-diphenyl-1,2-diaminoethane and optical rotation of (*SS*)-1,2-diphenyl-1,2-diaminoethane. Crystallographic details of the complexes **1a**–**c**, **2a**, and **3c**. (PDF)
Molecular formula strings of **1a**–**c to 3a**–**3c** with IC_50_ values of the MDA-MB 231 cell line (CSV)

## Notes

Crystallographic data have been deposited with the Cambridge Crystallographic Data Centre as supplementary publication no. CCDC - 2532650 (**1a_DCM**), CCDC - 2532651 (**1a_DMSO**), CCDC - 2532652 (**1b_DCM**), CCDC - 2532653 (**1c_DCM**), CCDC - 2532654 (**2a_DCM**) and CCDC - 2532655 (**3c_DMSO**). These data can be obtained free of charge via https://www.ccdc.cam.ac.uk/structures (or from the CCDC, 12 Union Road, Cambridge CB2 1EZ, UK; fax: (+44) 1223–336–033; or deposit@ccdc.cam.ac.uk).

## Abbreviations

AnV: annexin V
cpm: counts per minute
DACH: 1,2-diaminocyclohexane
DCM: dichloromethane
DIPHENYLSALENE: *N,N′*-disalicylidene-1,2-diphenyl-1,2-diaminoethane
DMSO: dimethyl sulfoxide
DNA: deoxyribonucleic acid
ECD: electronic circular dichroism
EPR: electron paramagnetic resonance
equiv: equivalent
FBS: fetal bovine serum
Fer-1: ferrostatin-1
FITC: fluorescein isothiocyanate
FT-IR: Fourier transform infrared
HEPES: 2-(4-(2-hydroxyethyl)­piperazinyl)­ethanesulfonic acid
HPLC: high performance liquid chromatography
HR-MS: high-resolution mass spectrometry
Hz: Hertz
MeCN: acetonitrile
MeOH: methanol
min: minute
MTT: 3-(4,5-dimethylthiazol-2-yl)-2,5-diphenyltetrazolium bromide
Nec-1: necrostatin-1
NMR: nuclear magnetic resonance
PBS: phosphate buffered saline
PI: propidium iodide
PUFA: polyunsaturated fatty acid
ROS: reactive oxygen species
RPMI: Rosewell Park Memorial Institute
Rt: retention time
rt: room temperature
Sal: salicylidene
SALDACH: *N,N*’-disalicylidene-1,2-diaminocyclohexane
SAR: structure–activity relationship
SE: standard error

## References

[1] Wainer I. W. (1993). Stereoisomers
in clinical oncology: Why it is important to know what the right and
left hands are doing. Ann. Oncol..

[2] Pendyala L., Kidani Y., Perez R., Wilkes J., Bernacki R. J., Creaven P. J. (1995). Cytotoxicity, cellular
accumulation and DNA binding
of oxaliplatin isomers. Cancer Lett..

[3] Liu F., Gou S., Chen F., Fang L., Zhao J. (2015). Study on antitumor
platinum­(II) complexes of chiral diamines with dicyclic species as
steric hindrance. J. Med. Chem..

[4] Arnesano F., Pannunzio A., Coluccia M., Natile G. (2015). Effect of chirality
in platinum drugs. Coord. Chem. Rev..

[5] Valentova, J. ; Lintnerová, L. Chirality in Anticancer Agents. In Current Topics in Chirality - From Chemistry to Biology; Akitsu, T. , Ed.; IntechOpen, 2021.

[6] Hille A., Wolf T., Schumacher P., Ott I., Gust R., Kircher B. (2011). Effects of metal salophene and saldach
complexes on
lymphoma and leukemia cells. Arch. Pharm. (Weinheim).

[7] Hille A., Gust R. (2009). Relationship between anticancer activity and stereochemistry of saldach
ligands and their iron­(III) complexes. Arch.
Pharm. (Weinheim).

[8] Sagasser J., Ma B. N., Baecker D., Salcher S., Hermann M., Lamprecht J., Angerer S., Obexer P., Kircher B., Gust R. (2019). A new approach
in cancer treatment: Discovery of chlorido­[N,N’-disalicylidene-1,2-phenylenediamine]­iron­(III)
complexes as ferroptosis inducers. J. Med. Chem..

[9] Descher H., Strich S. L., Hermann M., Enoh P., Kircher B., Gust R. (2023). Investigations on the influence of the axial ligand in [salophene]­iron­(III)
complexes on biological activity and redox behavior. Int. J. Mol. Sci..

[10] Bernkop-Schnürch A. D., Huber K., Clauser A., Cziferszky M., Leitner D., Talasz H., Hermann M., Hohloch S., Gust R., Kircher B. (2024). Design, synthesis, and biological
evaluation of novel halogenated chlorido­[*N,N′*-bis­(salicylidene)-1,2-bis­(3-methoxyphenyl)­ethylenediamine]­iron­(III)
complexes as anticancer agents. J. Biol. Inorg.
Chem..

[11] Bernkop-Schnürch A. D., Hermann M., Leitner D., Talasz H., Descher H. A., Hohloch S., Gust R., Kircher B. (2024). Transferrin receptor-mediated
cellular uptake of fluorinated chlorido­[*N,N′*-bis­(salicylidene)-1,2-phenylenediamine]­iron­(III) complexes. ACS Omega.

[12] Bernkop-Schnürch A. D., Chavooshi D., Descher H. A., Leitner D., Talasz H., Hermann M., Wurst K., Hohloch S., Gust R., Kircher B. (2023). Design, synthesis,
electrochemical, and biological
evaluation of fluorescent chlorido­[*N,N′*-bis­(methoxy/hydroxy)­salicylidene-1,2-bis­(4-methoxyphenyl)­ethylenediamine]­iron­(III)
complexes as anticancer agents. J. Med. Chem..

[13] Woldemariam G. A., Mandal S. S. (2008). Iron­(III)-salen damages DNA and induces apoptosis in
human cell via mitochondrial pathway. J. Inorg.
Biochem..

[14] Ansari K. I., Grant J. D., Woldemariam G. A., Kasiri S., Mandal S. S. (2009). Iron­(III)-salen
complexes with less DNA cleavage activity exhibit more efficient apoptosis
in MCF7 cells. Org. Biomol. Chem..

[15] Ansari K. I., Kasiri S., Grant J. D., Mandal S. S. (2011). Fe­(III)-salen and
salphen complexes induce caspase activation and apoptosis in human
cells. J. Biomol. Screen..

[16] Baecker D., Ma B. N., Sagasser J., Schultz L., Horschlager C., Weinreich M., Steiner L., Kircher B., Gust R. (2020). Amide and
ester derivatives of chlorido­[4-carboxy-1,2-disalicylideneaminobenzene]­iron­(III)
as necroptosis and ferroptosis inducers. Dalton
Trans..

[17] Dixon S. J., Lemberg K. M., Lamprecht M. R., Skouta R., Zaitsev E. M., Gleason C. E., Patel D. N., Bauer A. J., Cantley A. M., Yang W. S. (2012). Ferroptosis: an iron-dependent form of nonapoptotic
cell death. Cell.

[18] Jiang X., Stockwell B. R., Conrad M. (2021). Ferroptosis: Mechanisms, biology
and role in disease. Nat. Rev. Mol. Cell Biol..

[19] Vogtle F., Goldschmitt E. (1976). Die Diaza-*Cope*-Umlagerung. Chem. Ber..

[20] Vögtle F., Goldschmitt E. (1974). Dynamic stereochemistry of the degenerate
DiazaCope
rearrangement. Angew. Chem. Int. Ed. Engl..

[21] Shiryaev A. A., Goncharenko A. N., Burkhanova T. M., Alkhimova L. E., Babashkina M. G., Chandrasekaran R., Safin D. A. (2021). A chiral (1R,2R)-N,N′-bis-(salicylidene)-1,2-diphenyl-1,2-ethanediamine
Schiff base dye: Synthesis, crystal structure, Hirshfeld surface analysis,
computational study, photophysical properties and in silico antifungal
activity. J. Iran. Chem. Soc..

[22] Korendovych I. V., Rybak-Akimova E. V. (2003). (−)-(1S,2S)-N,N’-Bis­(salicylidene)-1,2-diphenyl-1,2-ethanediamine. Acta Cryst. E.

[23] Bruhn T., Schaumloffel A., Hemberger Y., Bringmann G. (2013). SpecDis: Quantifying
the comparison of calculated and experimental electronic circular
dichroism spectra. Chirality.

[24] Gust R., Heinrich H., Krauser R., Schönenberger H. (1999). [Meso- and
rac-1,2-bis­(4-fluorophenyl)­ethylenediamine]­chloro­[sulfinylbis­(methane)-S]­platinum­(II)
chloride new water soluble platinum complexes with high anti-breast
cancer activities. Inorg. Chim. Acta.

[25] Gust R., Niebler K., Schonenberger H. (1995). Investigation of the configurational
and conformational influences on the hormonal activity of 1,2-bis­(2,6-dichloro-4-hydroxyphenyl)­ethylenediamines
and of their platinum­(II) complexes. 1. Synthesis, estradiol receptor
affinity, and estrogenic activity of diastereomeric [*N*-alkyl- and *N,N*’-dialkyl-1,2- bis­(2,6-dichloro-4-hydroxyphenyl)­ethylenediamine]­dichloroplatinum­(II)
complexes. J. Med. Chem..

[26] Gust R., Schonenberger H. (1993). Mammary tumor
inhibiting [1,2-bis­(2,6-dihalo-3-hydroxyphenyl)­ethylenediamine]­platinum­(II)
complexes, III: Relationship between structure and estrogenic activity
of the diamine ligands, their sulfatoplatinum­(II) and diiodoplatinum­(II)
complexes. Arch. Pharm. (Weinheim).

[27] Gust R., Schonenberger H., Klement U., Range K. J. (1993). Aqua­[1-(2,6-dichloro-4-hydroxyphenyl)-2-
phenylethylenediamine]­sulfatoplatinum­(II) complexes with variable
substituents in the 2-phenyl ring, II: Correlation of molecular structure
and estrogenic activity of breast and prostate cancer inhibiting.
[erythro-1-(2,6-dichloro-4-hydroxyphenyl)-2-(2-halo-4- hydroxyphenyl)­ethylenediamine]­platinum­(II)
complexes. Arch. Pharm. (Weinheim).

[28] Wang J., Jian F., Zhuang R., Qiao Y. (2012). A new μ-oxo dimer
iron­(III) complex: Synthesis, characterization, and electrocatalysis. Synth. React. Inorg., Met.-Org., Nano-Met. Chem..

[29] Oyaizu K., Listiani Dewi E., Tsuchida E. (2001). A μ-oxo diiron­(III) complex
with a short Fe-Fe distance: Crystal structure of (μ-oxo)­bis­[*N,N′*-o-phenylenebis­(salicylideneiminato)­iron­(III)]. Inorg. Chim. Acta.

[30] Kerber W. D., Perez K. A., Ren C., Siegler M. A. (2014). Speciation of ferric
phenoxide Intermediates during the reduction of Iron­(III)-μ-oxo
dimers by hydroquinone. Inorg. Chem..

[31] Schmidt M.
R., Bernkop-Schnürch A. D., Stengel D., Kiechle M. A., Afzal K. B., Seybold A., Hermann M., Kircher B., Bernkop-Schnürch A. (2025). Assessing
the potential of chlorido­[N,N’-bis­(salicylidene)-1,2-phenylenediamine]­iron­(III):
Exploring delivery through lipid-based nanocarriers. Eur. J. Pharm. Sci..

[32] Schultze B., Oehlert W. (1960). Autoradiographic investigations of incorporation of
H3-thymidine into cells of the rat and mouse. Science.

[33] Altman F. P. (1976). Tetrazolium
salts and formazans. Prog. Histochem. Cytochem..

[34] Liang C. C., Park A. Y., Guan J. L. (2007). In vitro scratch assay: a convenient
and inexpensive method for analysis of cell migration in vitro. Nat. Protoc..

[35] Martinotti S., Ranzato E. (2019). Scratch wound healing assay. Methods Mol. Biol..

[36] Checa J., Aran J. M. (2020). Reactive oxygen species: Drivers of physiological and
pathological processes. J. Inflamm. Res..

[37] Minamikawa T., Sriratana A., Williams D. A., Bowser D. N., Hill J. S., Nagley P. (1999). Chloromethyl-X-rosamine
(MitoTracker Red) photosensitises
mitochondria and induces apoptosis in intact human cells. J. Cell Sci..

[38] Endale H. T., Tesfaye W., Mengstie T. A. (2023). ROS induced
lipid peroxidation and
their role in ferroptosis. Front. Cell. Dev.
Biol..

[39] Pap E. H., Drummen G. P., Post J. A., Rijken P. J., Wirtz K. W. (2000). Fluorescent
fatty acid to monitor reactive oxygen in single cells. Methods Enzymol..

[40] Stockwell B. R. (2022). Ferroptosis
turns 10: Emerging mechanisms, physiological functions, and therapeutic
applications. Cell.

[41] Dunford H. B. (2002). Oxidations
of iron­(II)/(III) by hydrogen peroxide: From aquo to enzyme. Coord. Chem. Rev..

[42] Su F., Descher H., Bui-Hoang M., Stuppner H., Skvortsova I., Rad E. B., Ascher C., Weiss A., Rao Z., Hohloch S. (2024). Iron­(III)-salophene catalyzes redox cycles that induce
phospholipid peroxidation and deplete cancer cells of ferroptosis-protecting
cofactors. Redox Biol..

[43] Vermes I., Haanen C., Steffens-Nakken H., Reutellingsperger C. (1995). A novel assay
for apoptosis. Flow cytometric detection of phosphatidylserine expression
on early apoptotic cells using fluorescein labelled Annexin V. J. Immunol. Methods.

[44] Balasubramanian K., Bevers E. M., Willems G. M., Schroit A. J. (2001). Binding of annexin
V to membrane products of lipid peroxidation. Biochemistry.

[45] Pietkiewicz S., Schmidt J. H., Lavrik I. N. (2015). Quantification of apoptosis and necroptosis
at the single cell level by a combination of Imaging Flow Cytometry
with classical Annexin V/propidium iodide staining. J. Immunol. Methods.

[46] Miotto G., Rossetto M., Di Paolo M. L., Orian L., Venerando R., Roveri A., Vuckovic A. M., Bosello
Travain V., Zaccarin M., Zennaro L. (2020). Insight
into the mechanism
of ferroptosis inhibition by ferrostatin-1. Redox Biol..

[47] Cao L., Mu W. (2021). Necrostatin-1 and necroptosis
inhibition: Pathophysiology and therapeutic
implications. Pharmacol. Res..

[48] Yuk H., Abdullah M., Kim D. H., Lee H., Lee S. J. (2021). Necrostatin-1
prevents ferroptosis in a RIPK1- and IDO-independent manner in hepatocellular
carcinoma. Antioxidants (Basel).

[49] Belavgeni A., Meyer C., Stumpf J., Hugo C., Linkermann A. (2020). Ferroptosis
and necroptosis in the kidney. Cell. Chem. Biol..

[50] Tang L., Liu S., Li S., Chen Y., Xie B., Zhou J. (2023). Induction
mechanism of ferroptosis, necroptosis, and pyroptosis: A novel therapeutic
target in nervous system diseases. Int. J. Mol.
Sci..

[51] Fu B., Lou Y., Wu P., Lu X., Xu C. (2024). Emerging role of necroptosis,
pyroptosis, and ferroptosis in breast cancer: New dawn for overcoming
therapy resistance. Neoplasia.

[52] Zhu W., Jang S., Xiong J., Ezhov R., Li X.-X., Kim T., Seo M. S., Lee Y.-M., Pushkar Y., Sarangi R. (2021). A mononuclear
non-heme iron­(III)-peroxo complex with an unprecedented
high O-O stretch and electrophilic reactivity. J. Am. Chem. Soc..

[53] Jennerwein M., Gust R., Müller R., Schönenberger H., Engel J., Berger M. R., Schmähl D., Seeber S., Osieka R., Atassi G. (1989). Tumor
inhibiting properties of stereoisomeric [1,2-bis­(3-hydroxyphenyl)
ethylenediamine]­dichloroplatinum­(II)-complexes, Part II: Biological
properties. Arch. Pharm. (Weinheim).

[54] Jennerwein M., Wappes B., Gust R., Schonenberger H., Engel J., Seeber S., Osieka R. (1988). Influence
of ring substituents
on the antitumor effect of dichloro­(1,2-diphenylethylenediamine)­platinum­(II)
complexes. J. Cancer Res. Clin. Oncol..

[55] Gaussian 16 Rev. A.03; Wallingford, CT, 2016 (accessed. October 2025).

[56] Dolomanov O. V., Bourhis L. J., Gildea R. J., Howard J. A. K., Puschmann H. (2009). OLEX2: a complete
structure solution, refinement and analysis program. J. Appl. Crystallogr..

[57] Sheldrick G. (2015). SHELXT - Integrated
space-group and crystal-structure determination. Acta Cryst. A.

[58] Sheldrick G. (2015). Crystal structure
refinement with SHELXL. Acta Cryst. C.

