# Supplementary material for: Stereochemical Aspects in the Context of the Structure–Activity Relationship of Chlorido[N,N′‑bis(chloro/bromosalicylidene)-1,2-diphenyl-1,2-diaminoethane]iron(III) Complexes
Source: J Med Chem. 2026 Apr 17;69(9):10183–200. doi: 10.1021/acs.jmedchem.5c03253 (PMC13181772; doi:10.1021/acs.jmedchem.5c03253)
Supplement: Supplementary file 1 [file jm5c03253_si_001.pdf]

Supporting Information

Stereochemical aspects in the context of the structure-activity relationship of chlorido[N,N'-bis(chloro/bromosalicylidene)-1,2-diphenyl-1,2-diaminoethane]iron(III) complexes

*Astrid Dagmar Bernkop-Schnürch[1], Stefanie Schwarz[2], Philipp Rigo[1], Daniel Leitner[3], Mostafa Alilou[4], Martin Hermann[5], Michael Seidl[3], Magnus Andre Kiechle[6], Sofie Hanifle[2,7], Stephan Hohloch[3], Brigitte Kircher[*2,7], Ronald Gust[*1]*

[1]Department of Pharmaceutical Chemistry, Institute of Pharmacy, CCB—Center for Chemistry and Biomedicine, University of Innsbruck, Innrain 80-82, 6020 Innsbruck, Austria

[2]Department of Internal Medicine V (Hematology and Oncology), Immunobiology and Stem Cell Laboratory, Medical University of Innsbruck, Anichstraße 35, 6020 Innsbruck, Austria

[3]Department of General, Inorganic and Theoretical Chemistry, University of Innsbruck, Innrain 80-82, 6020 Innsbruck, Austria

[4]Department of Pharmacognosy, Institute of Pharmacy, University of Innsbruck, Innrain 80-82, 6020 Innsbruck, Austria

[5]Department of Anesthesiology and Critical Care Medicine, Medical University of Innsbruck, Anichstraße 35, 6020 Innsbruck, Austria

[6]Department of Pharmaceutical Technology, Institute of Pharmacy, CCB—Center for Chemistry and Biomedicine, University of Innsbruck, Innrain 80-82, 6020 Innsbruck, Austria

[7]Tyrolean Cancer Research Institute, Innrain 66, 6020 Innsbruck, Austria

*Correspondence:
brigitte.kircher@i-med.ac.at; rgust@zedat.fu-berlin.de

Table of Contents

Chemistry.........S3
^{1}H NMR spectra of the ligands.........S3
^{13}C NMR spectra of the ligands.........S8
FT-IR spectra of the ligands.........S13
FT-IR spectra of the complexes.........S18
HPLC data of the complexes.........S23
EPR spectra of the complexes.........S27
Crystallographic details.........S28
Determination of stability.........S40
Biology.........S48
Proliferation.........S48
Metabolic activity.........S50
Scratch-Assay.........S52
Cell viability and cell death study.........S53

Chemistry

^{1}H NMR spectra of the ligands

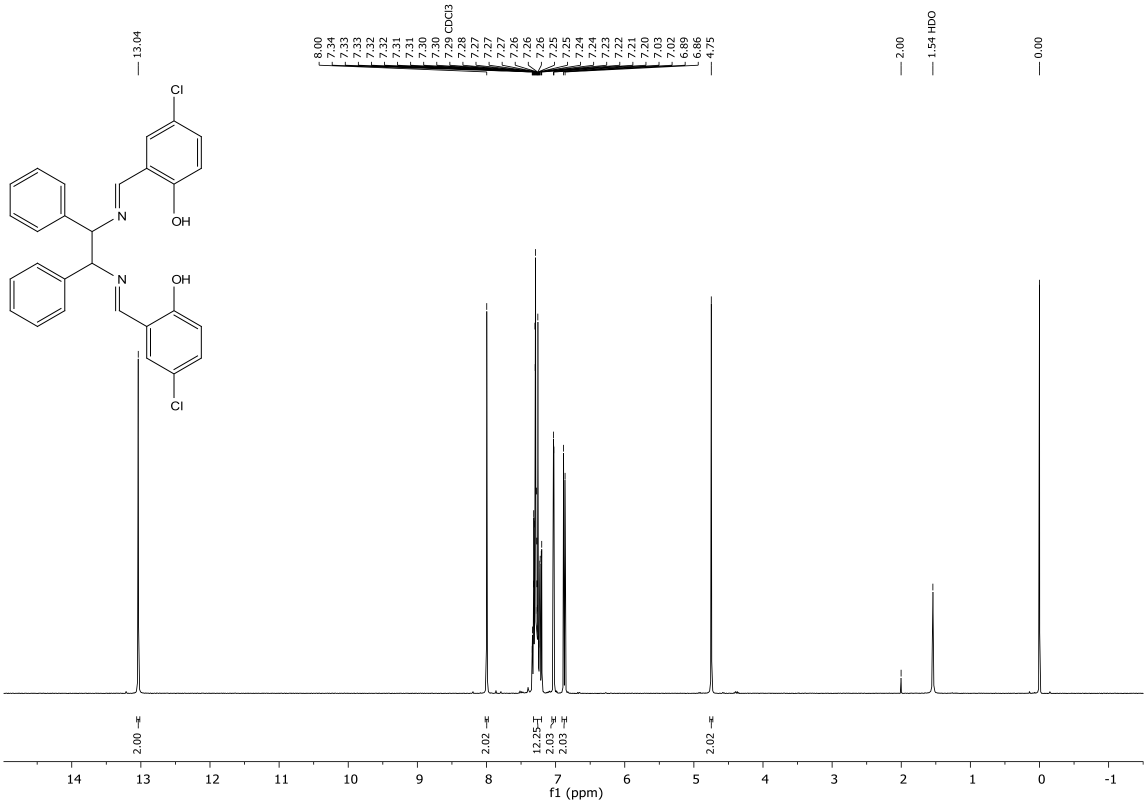

Figure S1: ^{1}H NMR spectrum (400 MHz, $CDCl_3$) of (*RS*)-*N*,*N′*-bis(5-chlorosalicylidene)-1,2-diphenyl-1,2-diaminoethane **(L1a)**: δ 13.04 (s, 2H, OH), 8.00 (s, 2H, N=CH), 7.36-7.18 (m, 12H), 7.03 (d, J = 2.6 Hz, 2H), 6.87 (d, J = 8.8 Hz, 2H), 4.75 (s, 2H, CH).

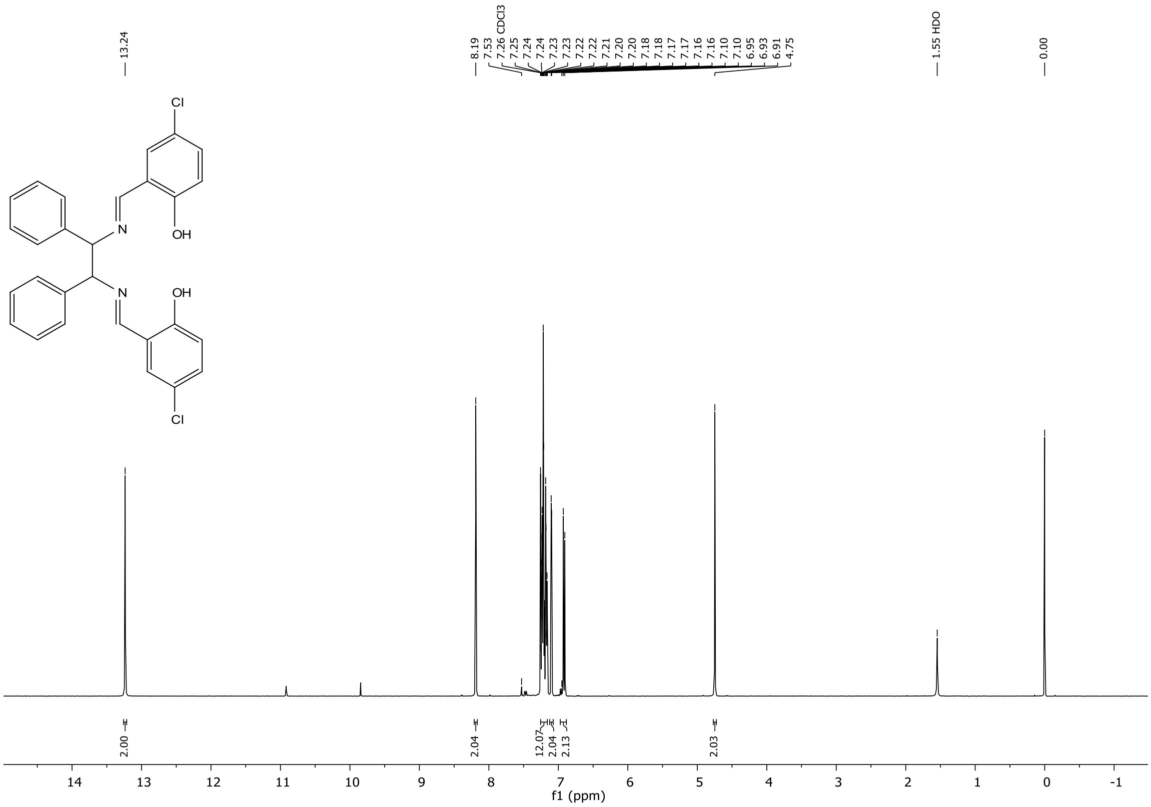

Figure S2: ^{1}H NMR spectrum (400 MHz, $CDCl_3$) of *(RR/SS)*-*N*,*N′*-bis(5-chlorosalicylidene)-1,2-diphenyl-1,2-diaminoethane **(L1b)**: δ 13.24 (s, 2H, OH), 8.19 (s, 2H, N=CH), 7.27-7.13 (m, 12H), 7.10 (d, J = 2.6 Hz, 2H), 6.92 (d, J = 8.8 Hz, 2H), 4.75 (s, 2H, CH).

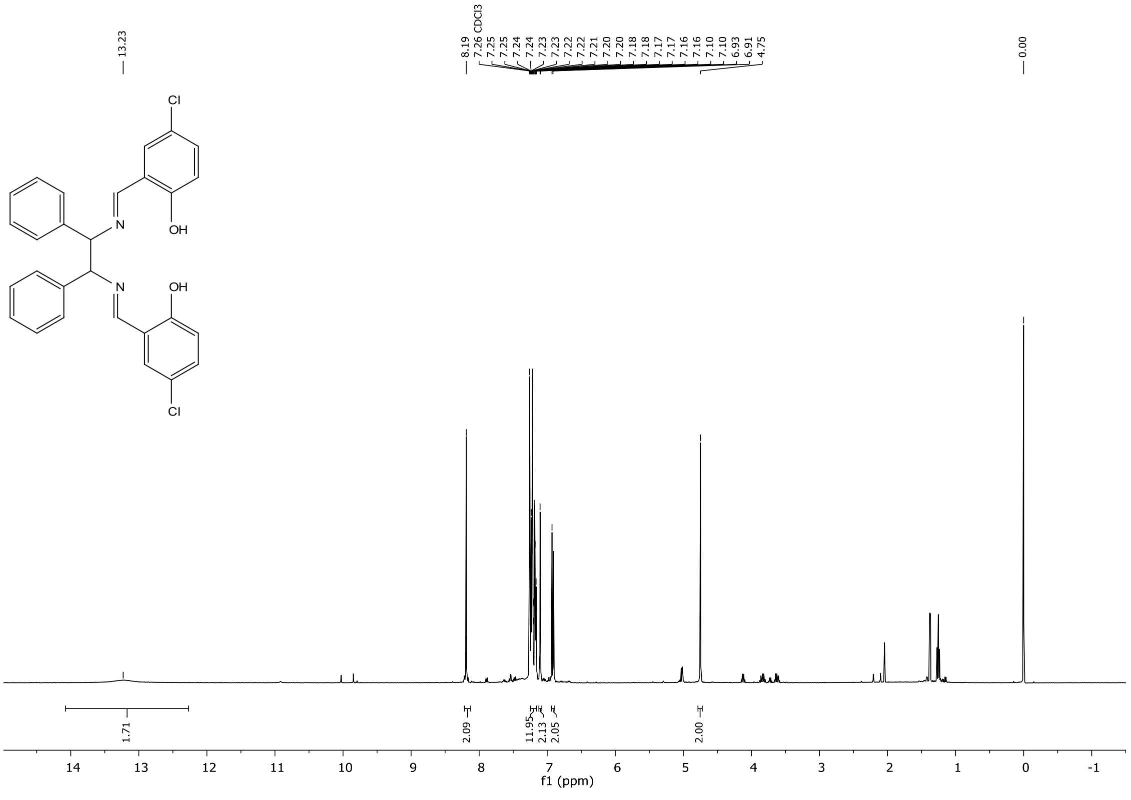

Figure S3: ^{1}H NMR spectrum (400 MHz, $CDCl_3$) of *(SS)*-*N*,*N′*-bis(5-chlorosalicylidene)-1,2-diphenyl-1,2-diaminoethane **(L1c)**: δ 13.23 (s, 2H, OH), 8.19 (s, 2H, N=CH), 7.25-7.16 (m, 12H), 7.10 (d, J = 2.6 Hz, 2H), 6.91 (d, J = 8.8 Hz, 2H), 4.75 (s, 2H, CH).

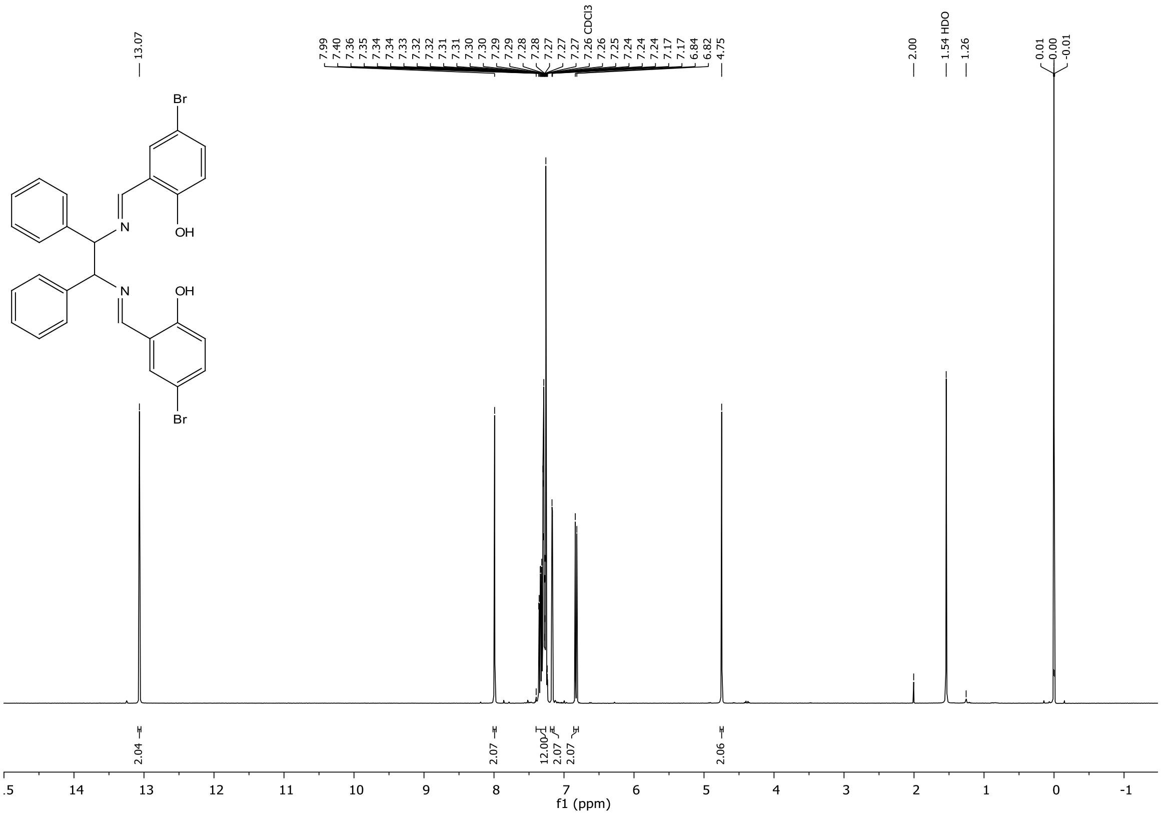

Figure S4: ^{1}H NMR spectrum (400 MHz, $CDCl_3$) of (*RS*)-*N*,*N′*-bis(5-bromosalicylidene)-1,2-diphenyl-1,2-diaminoethane **(L2a)**: δ 13.07 (s, 2H, OH), 7.99 (s, 2H, N=CH), 7.38-7.21 (m, 12H) 7.17 (d, J = 2.5 Hz, 2H), 6.83 (d, J = 8.8 Hz, 2H), 4.75 (s, 2H, CH).

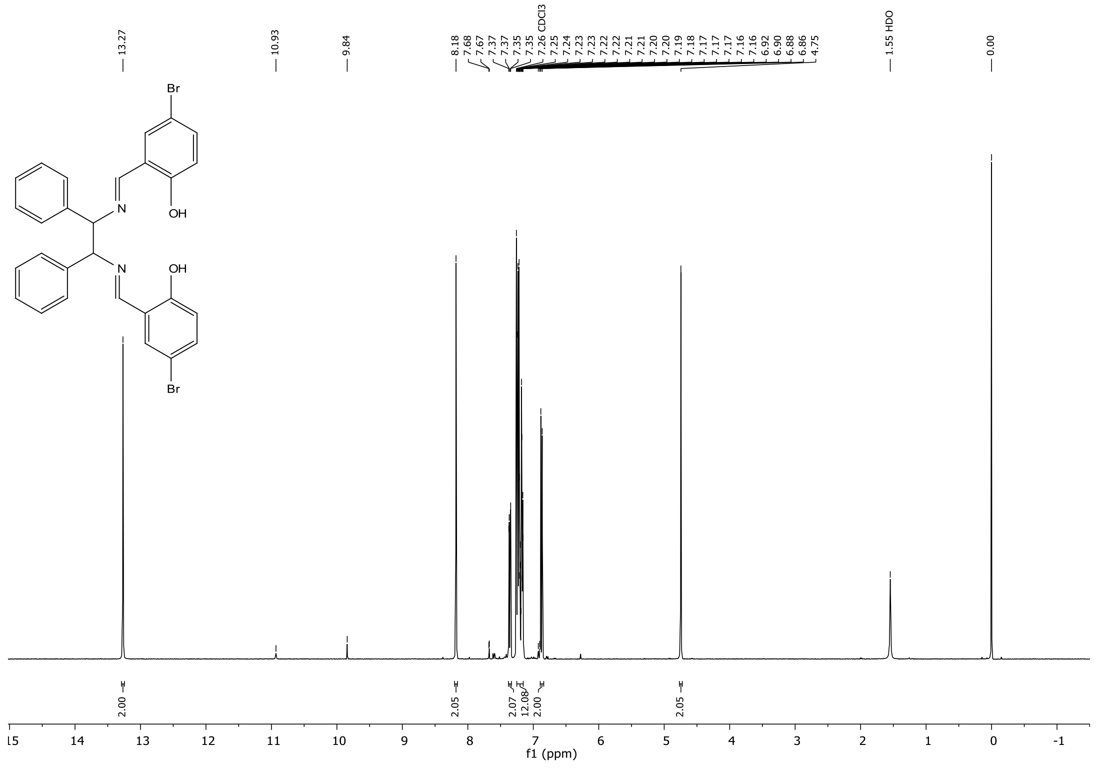

Figure S5: ^{1}H NMR spectrum (400 MHz, $CDCl_3$) of *(RR/SS)*-*N*,*N′*-bis(5-bromosalicylidene)-1,2-diphenyl-1,2-diaminoethane **(L2b)**: δ 13.27 (s, 2H, OH), 8.18 (s, 2H, N=CH), 7.36 (dd, J = 8.8, 2.4 Hz, 2H), 7.27-7.13 (m, 12H), 6.8 (d, J = 8.8 Hz, 2H), 4.75 (s, 2H, CH).

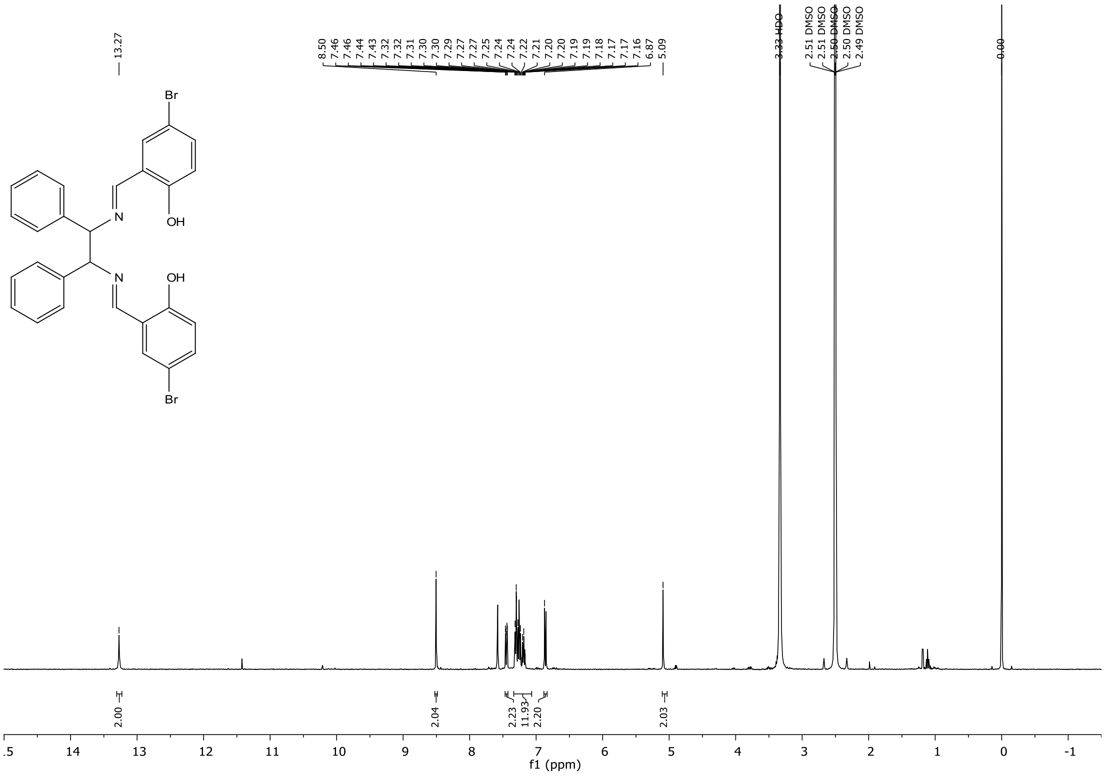

Figure S6: ^{1}H NMR spectrum (400 MHz, DMSO d_6) of (*SS*)-*N,N'*-bis(5-bromosalicylidene)-1,2-diphenyl-1,2-diaminoethane **(L2c)**: δ 13.27 (s, 2H, OH), 8.50 (s, 2H, N=CH), 7.46 (dd, J = 8.8, 2.5 Hz, 2H), 7.32-7.16 (m, 12H), 6.87 (d, J = 8.8 Hz, 2H), 5.09 (s, 2H, CH).

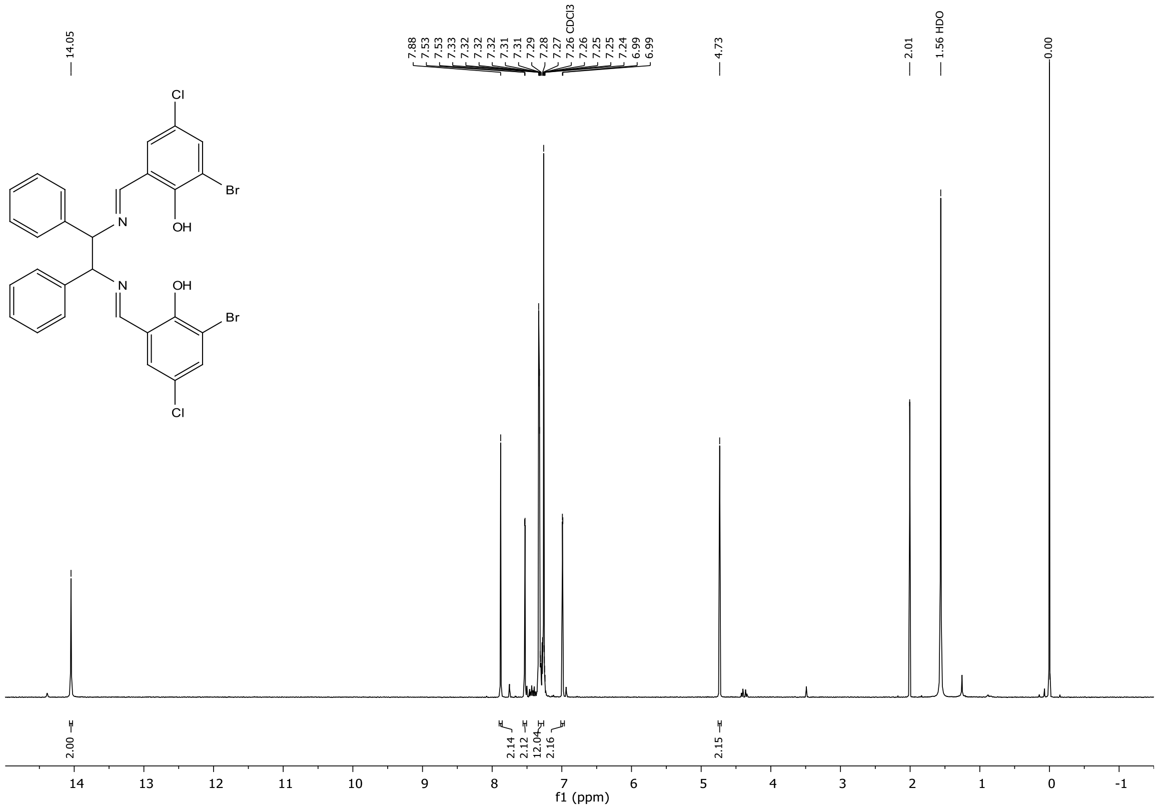

Figure S7: ^{1}H NMR spectrum (400 MHz, $CDCl_3$) of (*RS*)-*N,N'*-bis(3-bromo5-chlorosalicylidene)-1,2-diphenyl-1,2-diaminoethane (**L3a**): δ 14.05 (s, 2H, OH), 7.88 (s, 2H, N=CH), 7.53 (d, J = 2.5 Hz, 2H), 7.33-6.99 (m, 12H), 4.73 (s, 2H, CH).

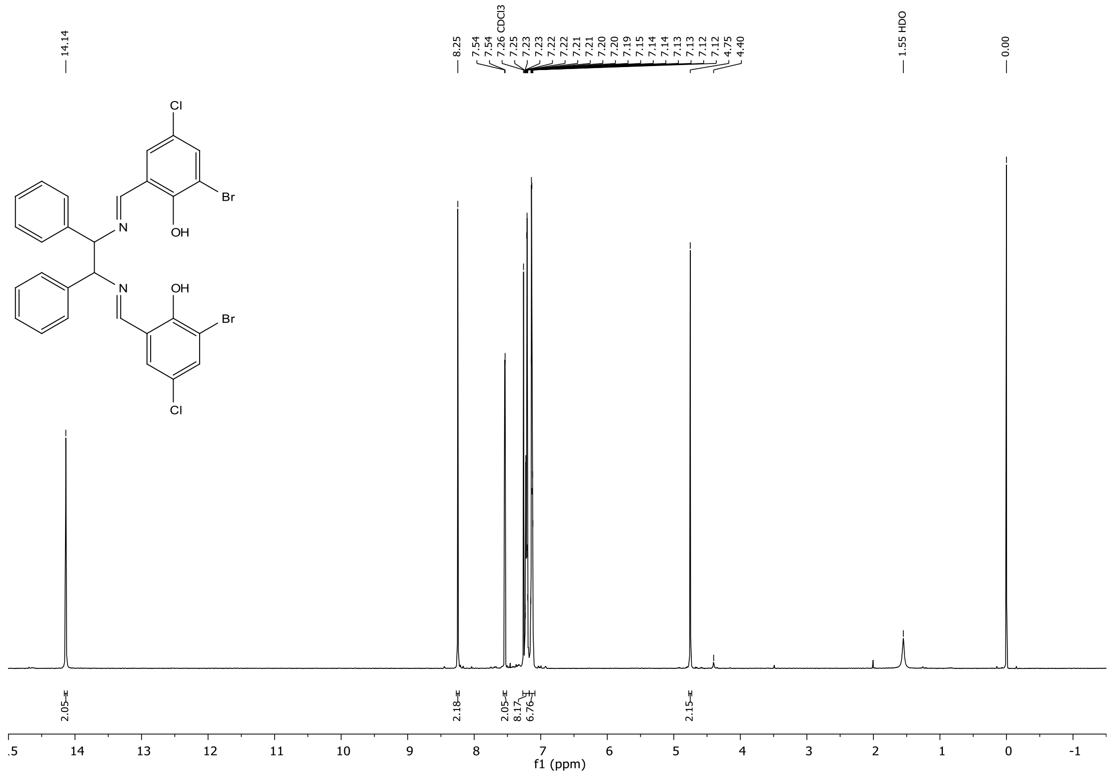

Figure S8: ^{1}H NMR spectrum (400 MHz, $CDCl_3$) of (*RR/SS*)-*N,N'*-bis(3-bromo5-chlorosalicylidene)-1,2-diphenyl-1,2-diaminoethane **(L3b)**: δ 14.14 (s, 2H, OH), 8.25 (s, 2H, N=CH), 7.54 (d, J = 2.4 Hz, 2H), 7.26-7.17 (m, 12H), 4.75 (s, 2H, CH).

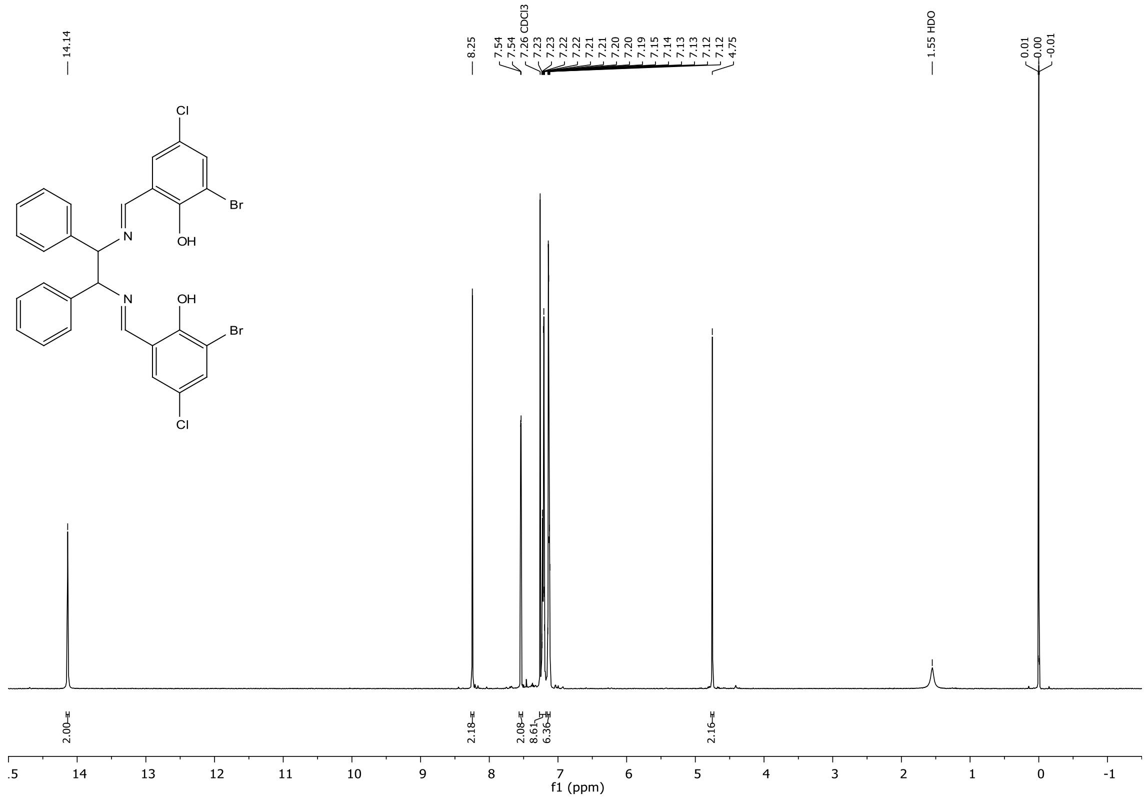

Figure S9: ^{1}H NMR spectrum (400 MHz, $CDCl_3$) of *(SS)-N,N'*-bis(3-bromo5-chlorosalicylidene)-1,2-diphenyl-1,2-diaminoethane (**L3c**): δ 14.14 (s, 2H, OH), 8.25 (s, 2H, N=CH), 7.54 (d, J = 2.5 Hz, 2H), 7.27-7.10 (m, 12H), 4.76 (s, 2H, CH).

^{13}C NMR spectra of the ligands

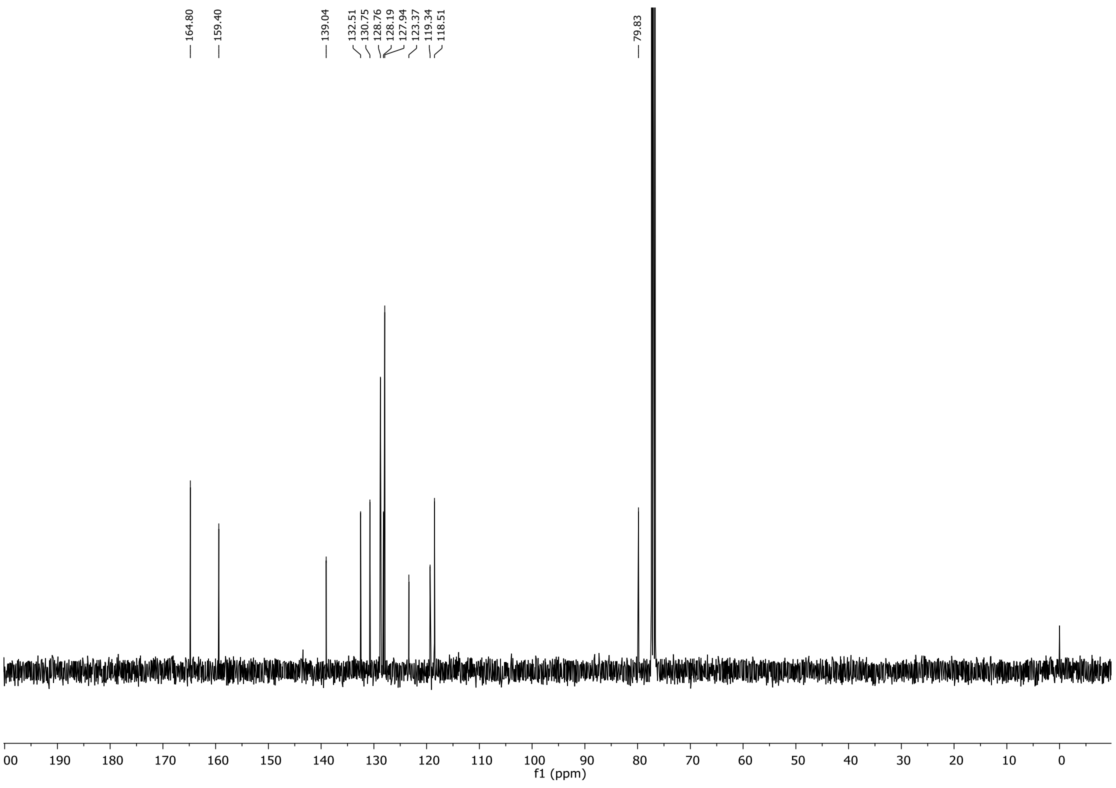

Figure S10: ^{13}C NMR spectrum (101 MHz, $CDCl_3$) of (*RS*)-*N*,*N'*-bis(5-chlorosalicylidene)-1,2-diphenyl-1,2-diaminoethane (**L1a**): δ 164.80 (2C), 159.40 (2C), 139.04 (1C), 132.51 (3C), 130.76 (2C), 128.76 (4C), 128.19 (3C), 127.94 (5C), 123.37 (1C), 119.34 (1C), 118.51 (2C), 79.83 (2C).

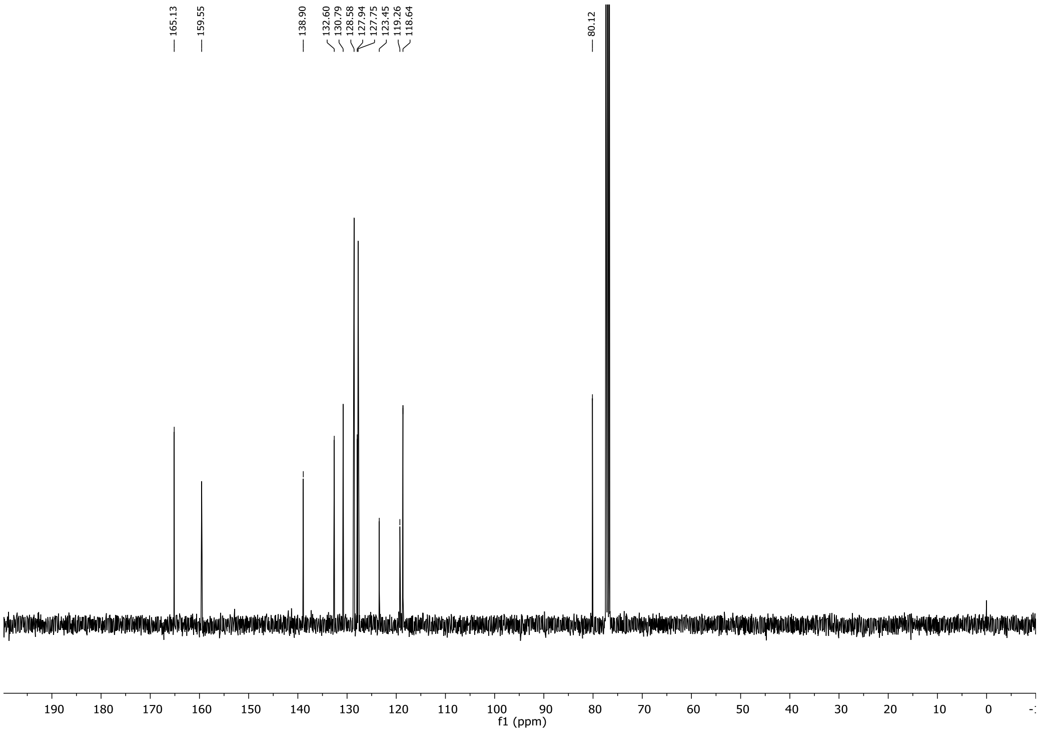

Figure S11: ^{13}C NMR spectrum (101 MHz, $CDCl_3$) of (*RR*/*SS*)-*N*,*N'*-bis(5-chlorosalicylidene)-1,2-diphenyl-1,2-diaminoethane (**L1b**): δ 165.13 (2C), 159.55 (2C), 138.90 (1C), 132.60 (3C), 130.79 (2C), 128.58 (5C), 127.94 (2C), 127.75 (5C), 123.45 (1C), 119.26 (1C), 118.64 (2C), 80.12 (2C).

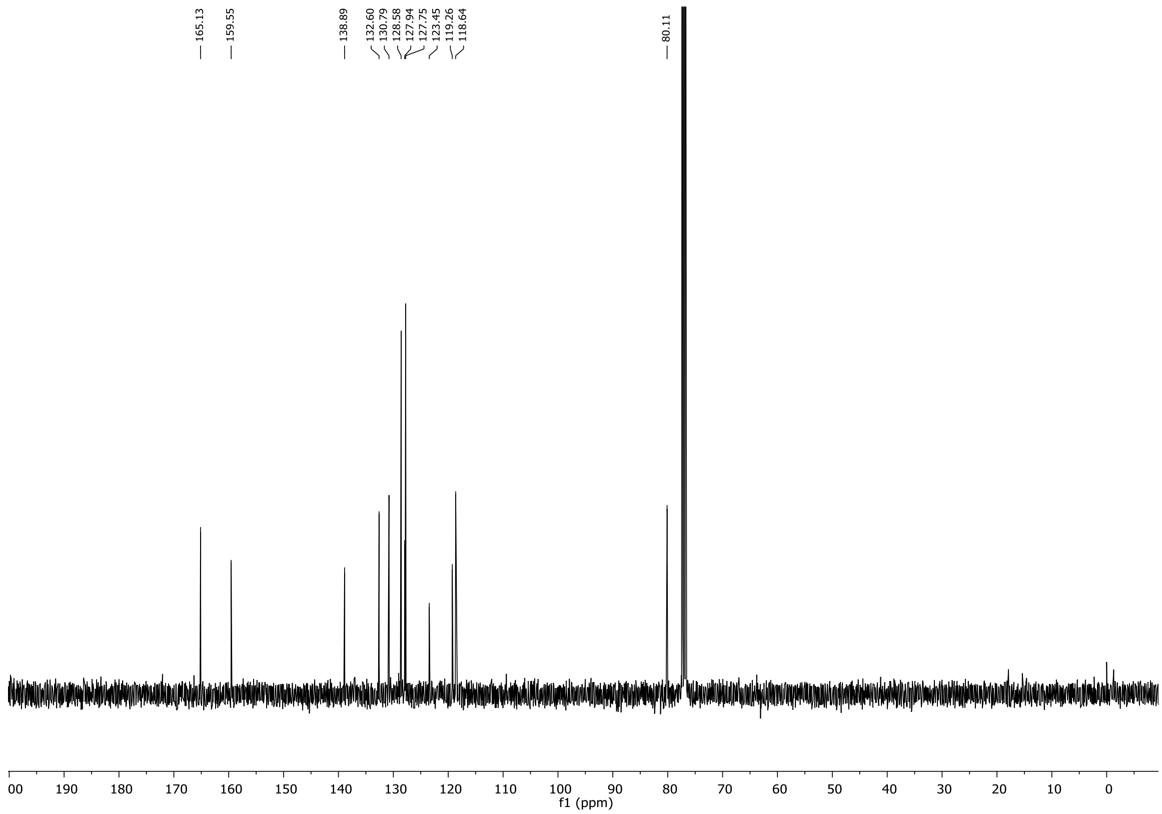

Figure S12: ^{13}C NMR spectrum (101 MHz, $CDCl_3$) of *(SS)*-*N*,*N′*-bis(5-chlorosalicylidene)-1,2-diphenyl-1,2-diaminoethane (**L1c**): δ 165.13 (2C), 159.55 (2C), 138.89 (1C), 132.60 (3C), 130.79 (2C), 128.58 (5C), 127.94 (2C), 127.75 (5C), 123.45 (1C), 119.26 (1C), 118.64 (2C), 80.11 (2C).

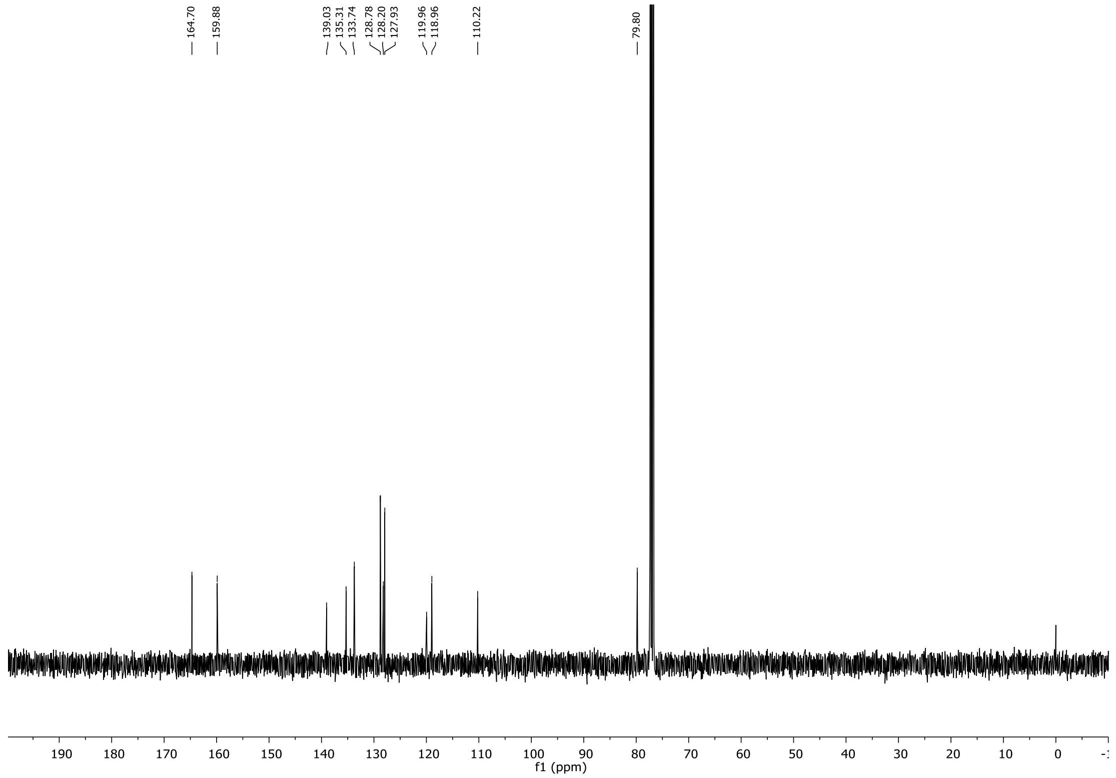

Figure S13: ^{13}C NMR spectrum (101 MHz, $CDCl_3$) of (*RS*)-*N*,*N′*-bis(5-bromosalicylidene)-1,2-diphenyl-1,2-diaminoethane (**L2a**): δ 164.70 (3C), 159.88 (1C), 139.03 (1C), 135.31 (2C), 133.74 (3C), 128.78 (5C), 128.21 (2C), 127.93 (4C), 119.97 (1C), 118.96 (3C), 110.22 (1C), 79.80 (2C).

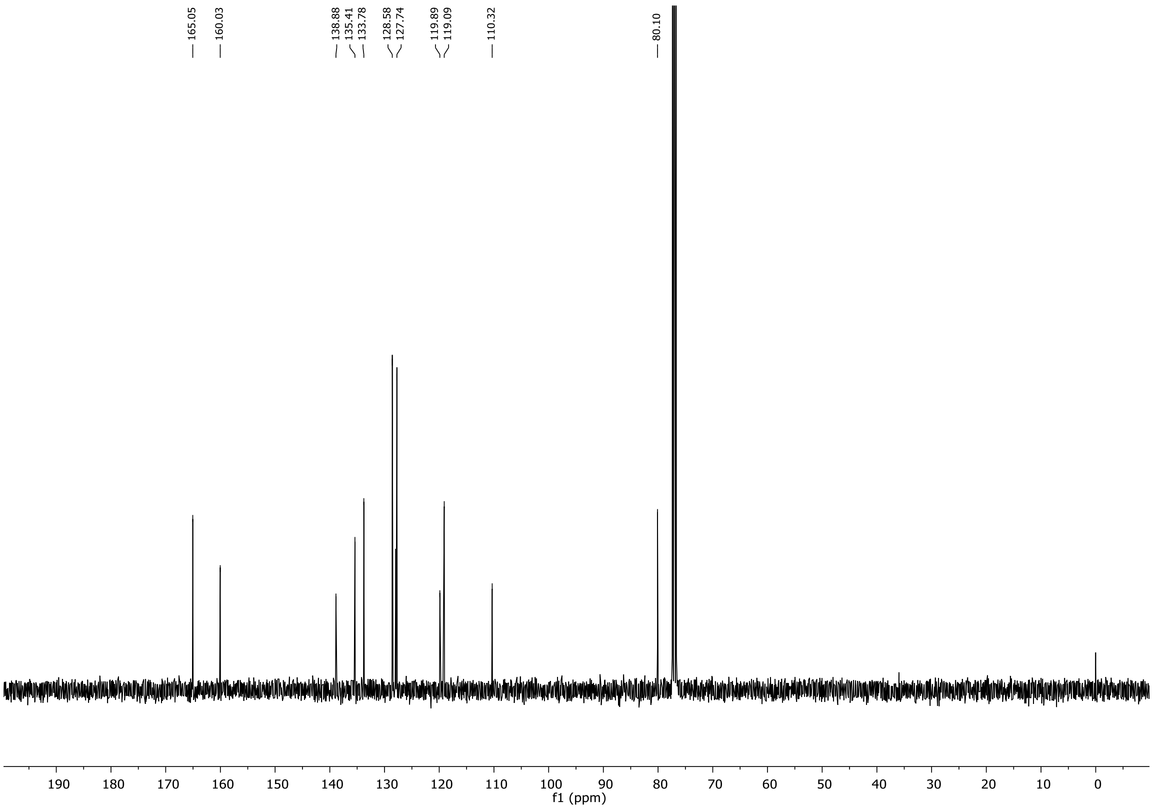

Figure S14: ^{13}C NMR spectrum (101 MHz, $CDCl_3$) of *(RR/SS)*-*N,N'*-bis(5-bromosalicylidene)-1,2-diphenyl-1,2-diaminoethane (**L2b**): δ 165.05 (3C), 160.03 (1C), 138.88 (1C), 135.41 (2C), 133.78 (2C), 128.58 (5C), 127.95 (2C), 127.74 (4C), 119.89 (2C), 119.08 (3C), 110.32 (1C), 80.10 (2C).

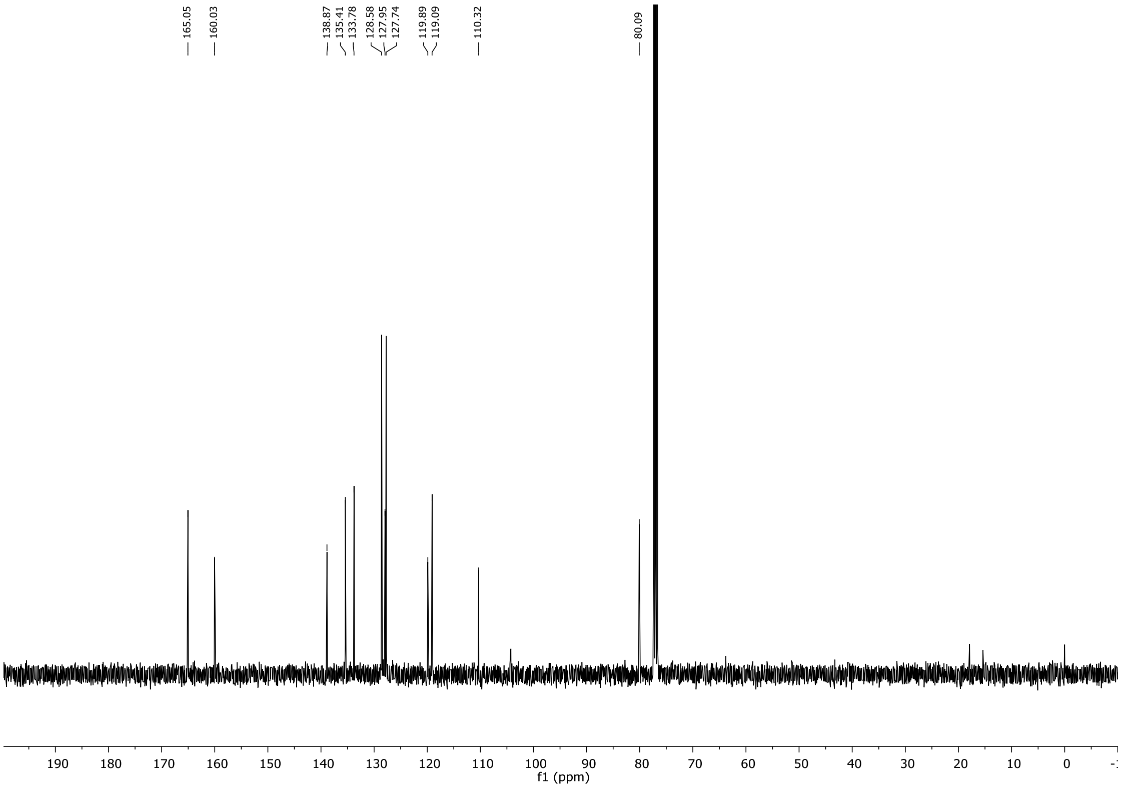

Figure S15: ^{13}C NMR spectrum (101 MHz, $CDCl_3$) of (*SS*)-*N,N'*-bis(5-bromosalicylidene)-1,2-diphenyl-1,2-diaminoethane (**L2c**): δ 165.05 (3C), 160.03 (1C), 138.87 (1C), 135.41 (2C), 133.78 (2C), 128.58 (5C), 127.95 (2C), 127.74 (4C), 119.89 (2C), 119.09 (3C), 110.32 (1C), 80.09 (2C).

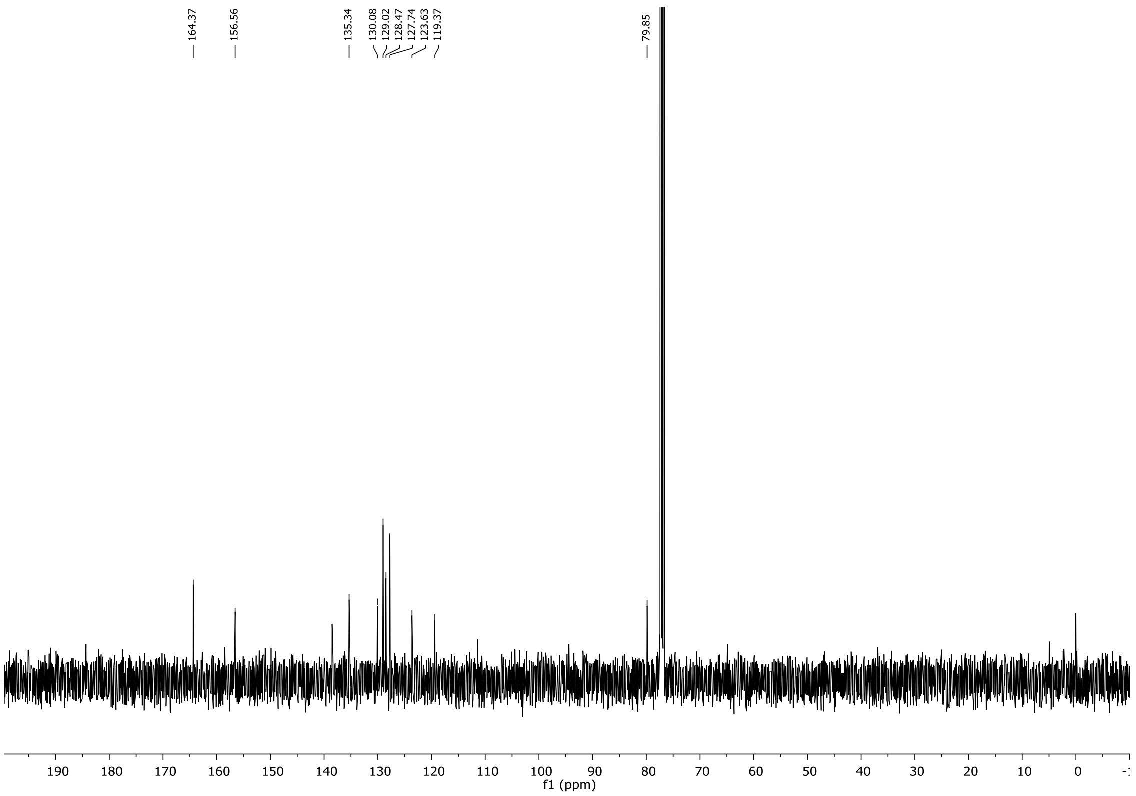

Figure S16: ^{13}C NMR spectrum (101 MHz, $CDCl_3$) of (*RS*)-*N*,*N'*-bis(3-bromo-5-chlorosalicylidene)-1,2-diphenyl-1,2-diaminoethane (**L3a**): δ 164.37 (3C), 156.56 (1C), 135.34 (3C), 130.08 (2C), 129.02 (6C), 128.47 (3C), 127.74 (4C), 123.63 (2C), 119.37 (2C), 79.85 (2C).

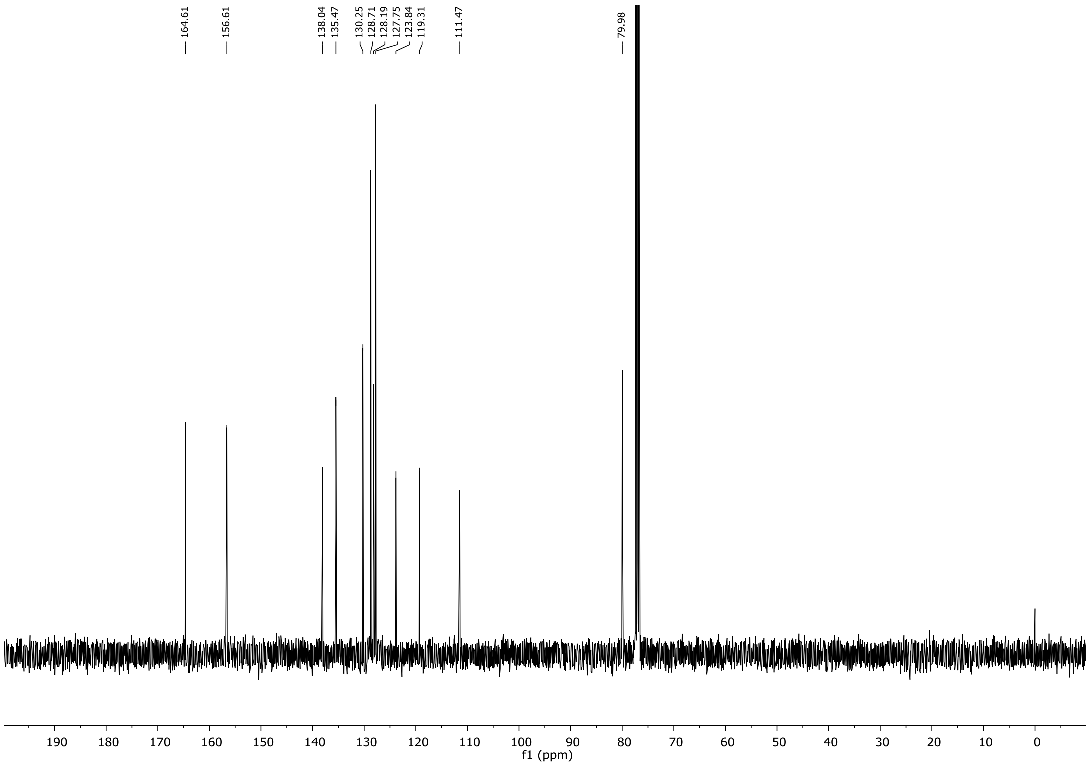

Figure S17: ^{13}C NMR spectrum (101 MHz, $CDCl_3$) of (*RR/SS*)-*N*,*N'*-bis(3-bromo-5-chlorosalicylidene)-1,2-diphenyl-1,2-diaminoethane (**L3b**): δ 164.61 (3C), 156.61 (1C), 138.04 (1C), 135.47 (2C), 130.25 (2C), 128.71 (5C), 128.19 (3C), 127.75 (5C), 123.84 (1C), 119.31 (1C), 111.47 (2C), 79.98 (2C).

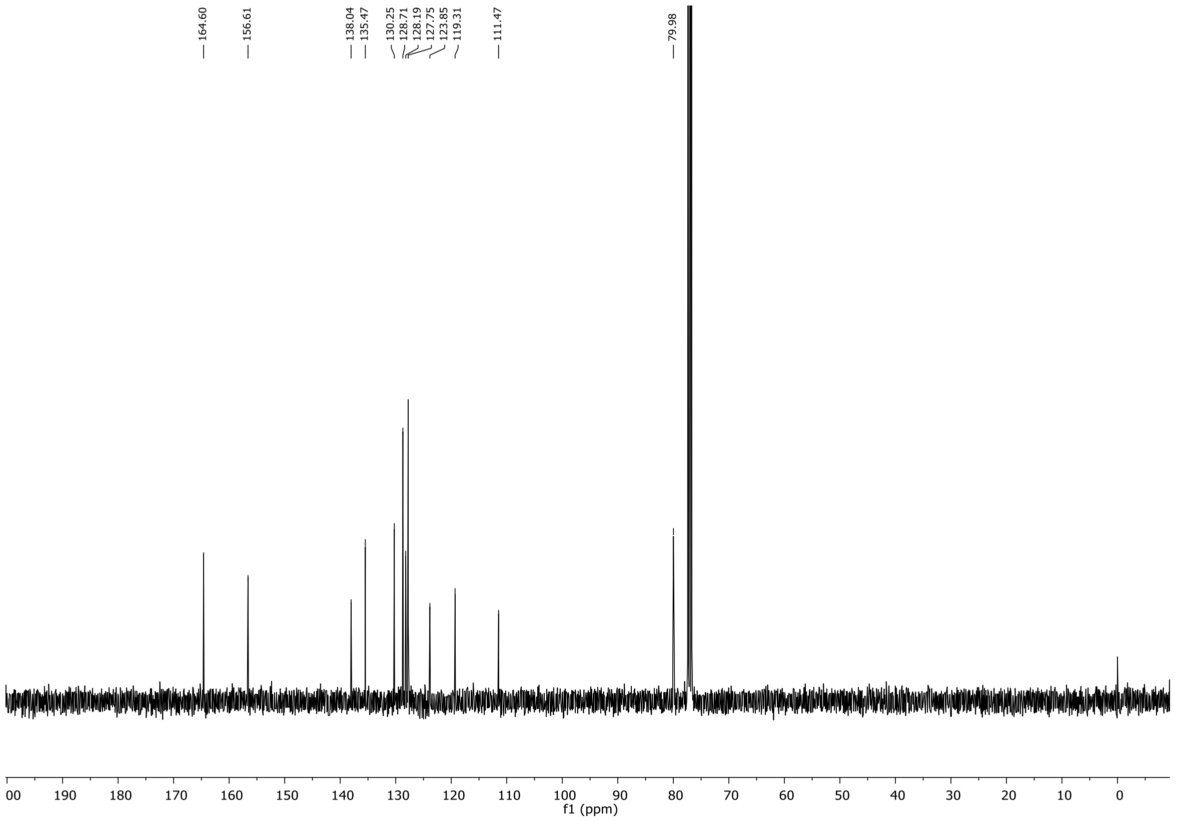

Figure S18: ^{13}C NMR spectrum (101 MHz, $CDCl_3$) of *(SS)-N,N′*-bis(3-bromo-5-chlorosalicylidene)-1,2-diphenyl-1,2-diaminoethane (**L3c**): δ 164.60 (3C), 156.61 (2C), 138.04 (1C), 135.47 (2C), 130.25 (2C), 128.71 (5C), 128.19 (3C), 127.75 (4C), 123.84 (1C), 119.31 (1C), 111.47 (2C), 79.98 (2C).

FT-IR spectra of the ligands

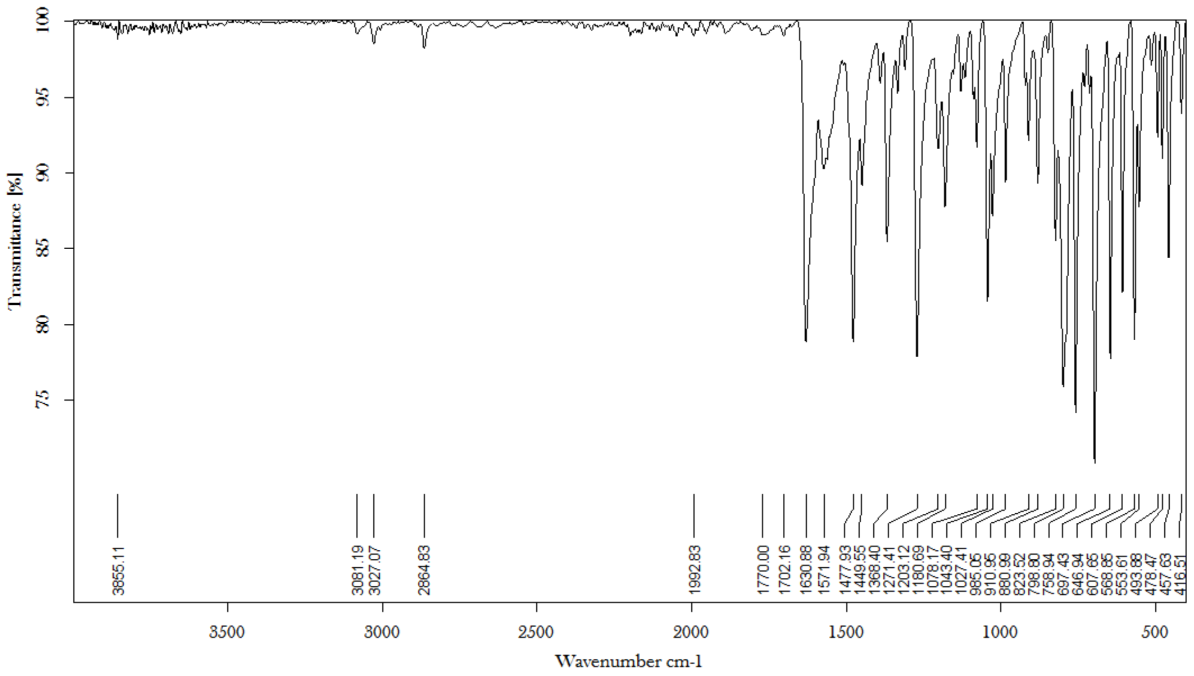

Figure S19: FT-IR spectrum of (*RS*)-*N*,*N′*-bis(5-chlorosalicylidene)-1,2-diphenyl-1,2-diaminoethane (**L1a**): $\bar{\nu}$ = 3081 w; (C=N) 1630 s; 1571 w; 1477 s; 1449 m; 1368 m; 1271 s; 1203 m; 1043 s; 1027 m; 880 w; 697 ss: 457 m.

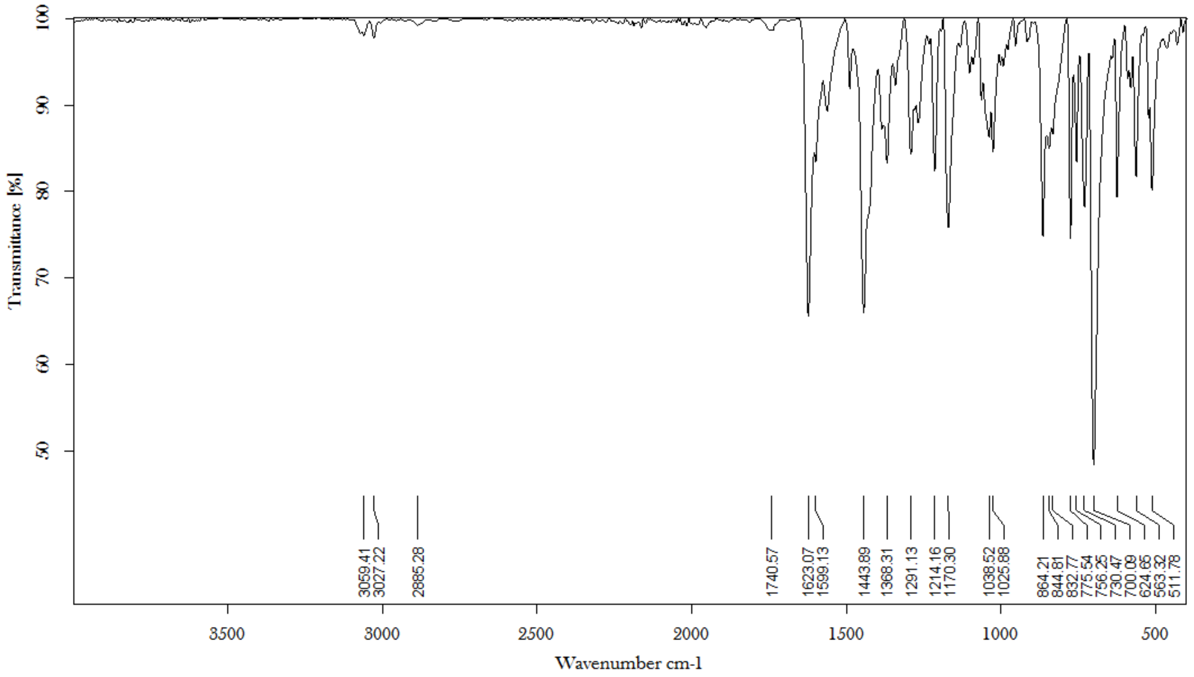

Figure S20: FT-IR spectrum of *(RR/SS)*-*N*,*N′*-bis(5-chlorosalicylidene)-1,2-diphenyl-1,2-diaminoethane (**L1b**): $\bar{\nu}$ = 3059 w; (C=N) 1623 s; 1599 w; 1443 s; 1291 m; 1214 m; 1170 m; 1038 w; 1025 m; 864 m; 700 ss; 511 m.

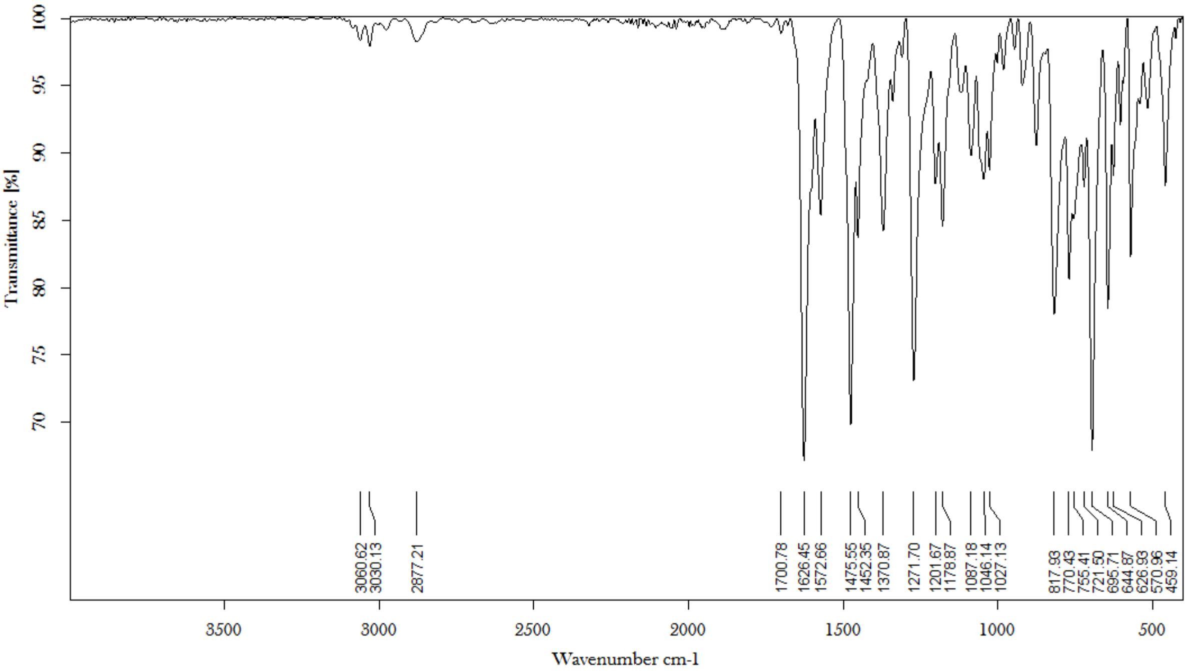

Figure S21: FT-IR spectrum of *(SS)*-*N*,*N′*-bis(5-chlorosalicylidene)-1,2-diphenyl-1,2-diaminoethane (**L1c**): ν̅ =3060 w; (C=N) 1626 s; 1475 s; 1452 m; 1370 m; 1271 s; 1201 w; 1178 m; 811 m; 770m; 695 s; 459 m.

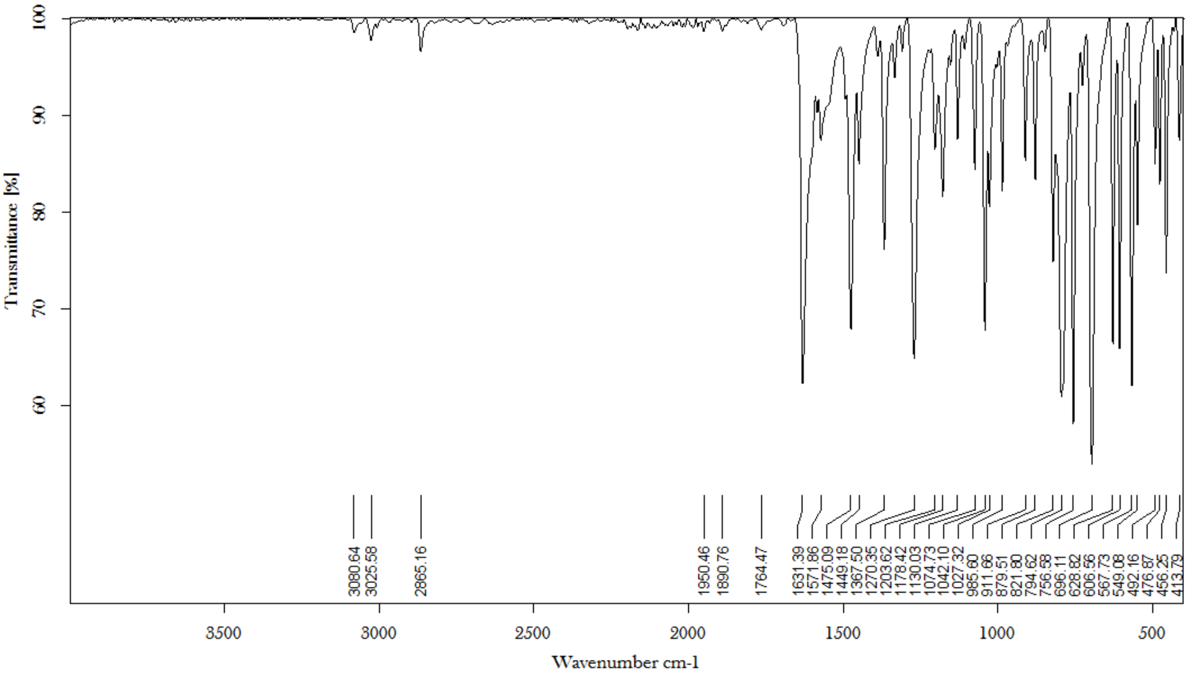

Figure S22: FT-IR spectrum of (*RS*)-*N*,*N′*-bis(5-bromosalicylidene)-1,2-diphenyl-1,2-diaminoethane (**L2a**): ν̅ =3080 w; (C=N) 1631 s; 1571 w; 1475 s; 1270 s; 1203 w; 1178 m; 1042 s; 1027 m; 821m; 756 s; 696 s; 492 w; 456 m.

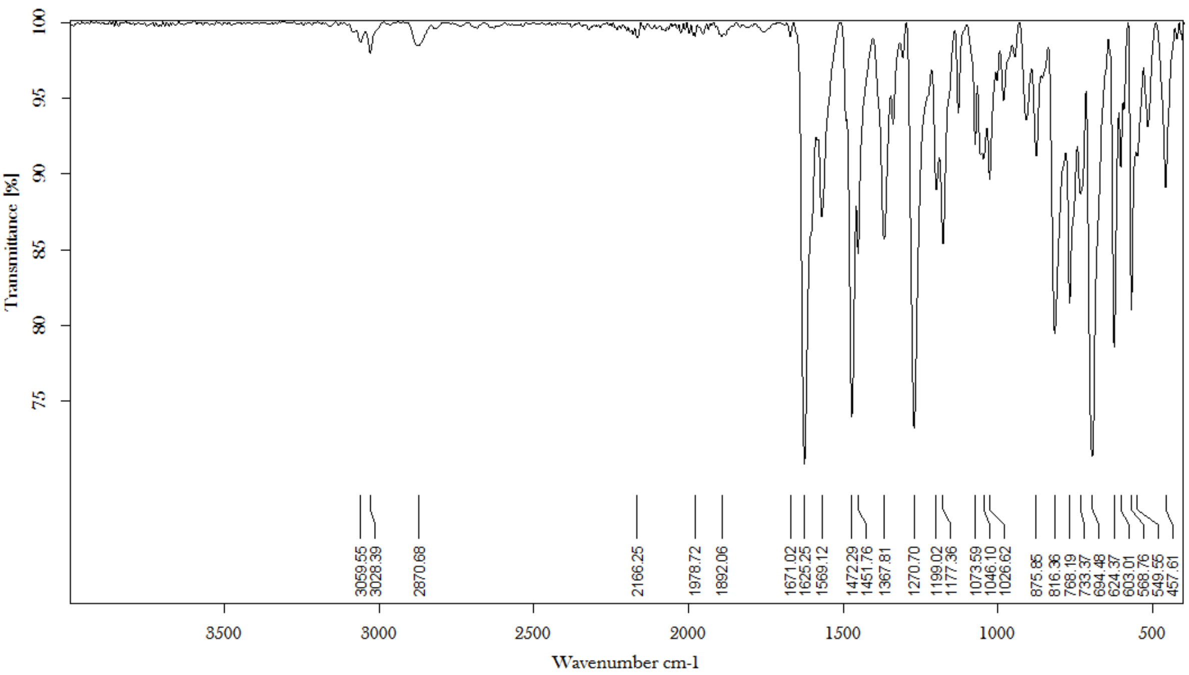

Figure S23: FT-IR spectrum of *(RR/SS)*-*N,N'*-bis(5-bromosalicylidene)-1,2-diphenyl-1,2-diaminoethane (**L2b**): $\bar{\nu}$ =3059 w; (C=N) 1625 s; 1569 w; 1472 s; 1451 m; 1270 s; 1199 w; 1177 m; 1073 w; 816 m; 768 m; 694 s; 457 m.

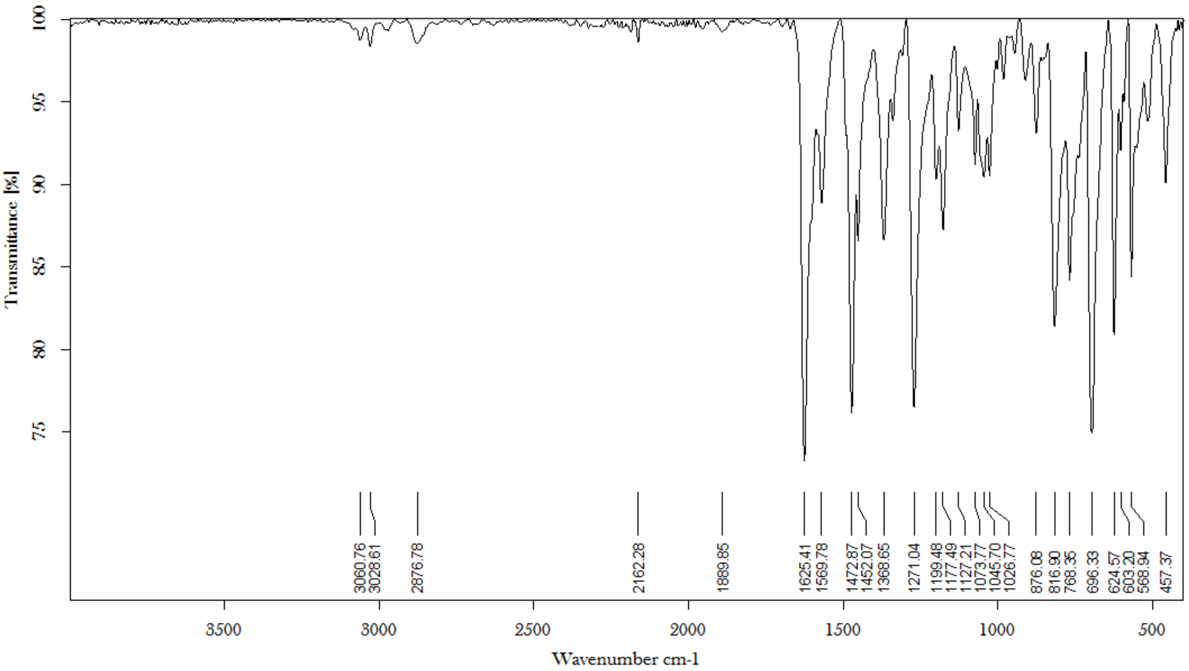

Figure S24: FT-IR spectrum of (*SS*)-*N,N'*-bis(5-bromosalicylidene)-1,2-diphenyl-1,2-diaminoethane (**L2c**): $\bar{\nu}$ =3060 w; (C=N) 1625 s; 1569 m; 1472 s; 1452 w; 1368 m; 1271 s; 1199 w; 1177 m; 1026 m; 816 s; 696 s; 457 m.

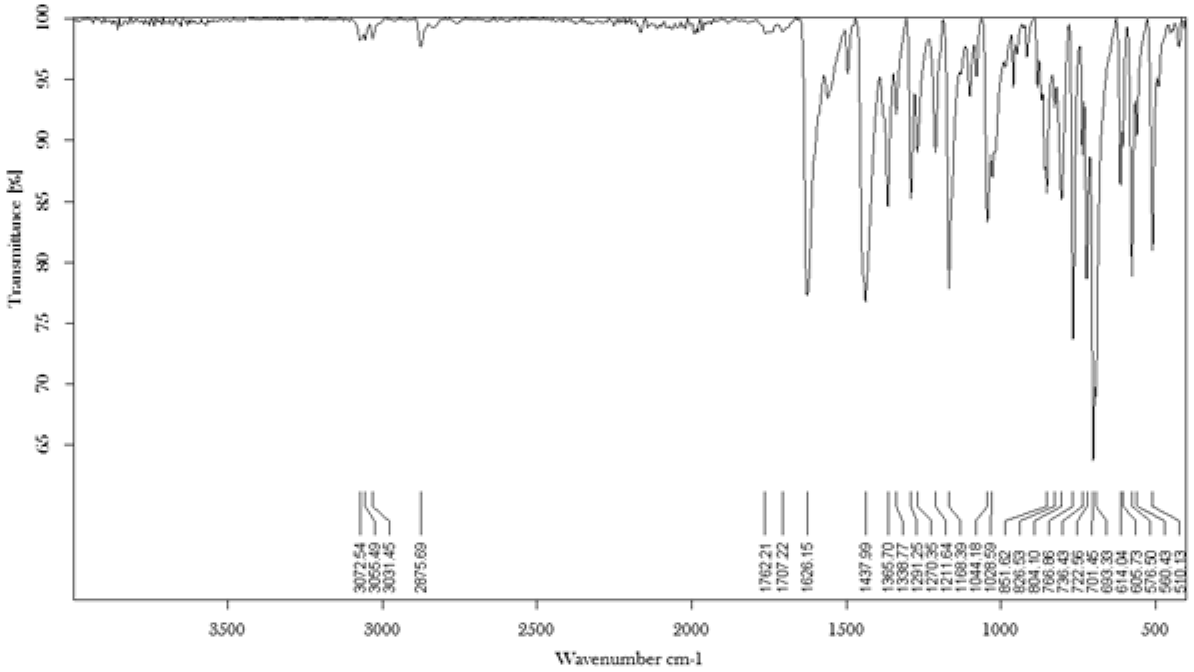

Figure S25: FT-IR spectrum of (*RS*)-*N*,*N*′-bis(3-bromo-5-chlorosalicylidene)-1,2-diphenyl-1,2-diaminoethane (**L3a**): $\bar{\nu}$ =3072 w; (C=N) 1626 s; 1437 s; 1291 m; 1270 m; 1168 s; 1044 m; 804 m; 766 s; 736 w; 701 ss; 576 m; 510 m.

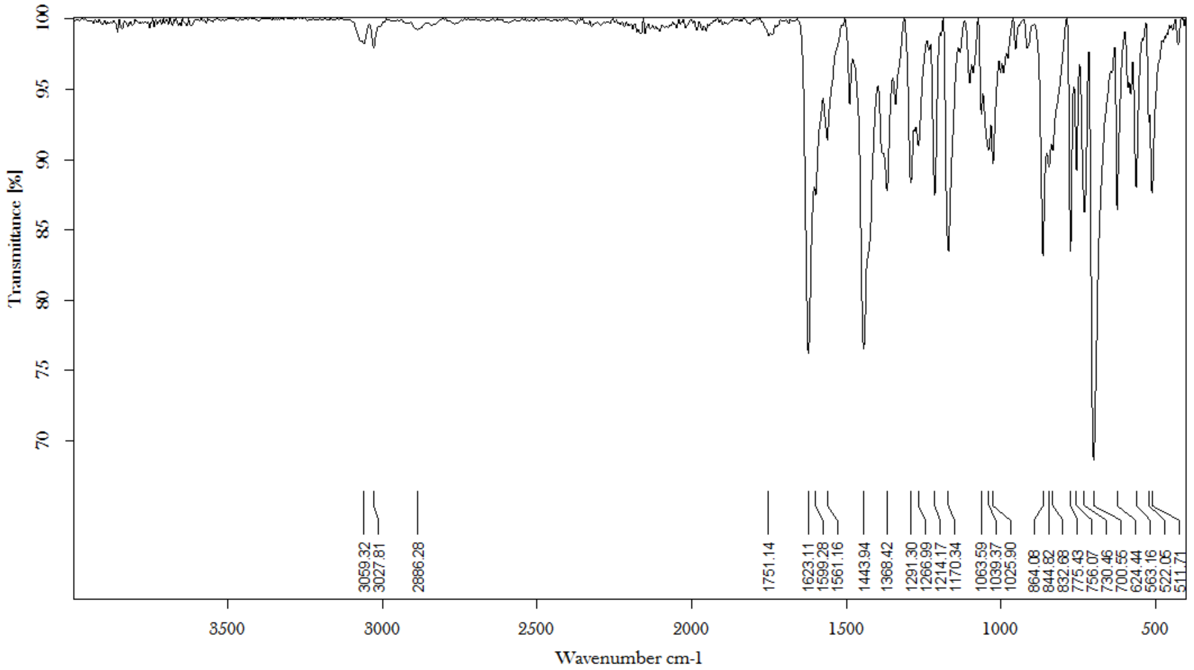

Figure S26: FT-IR spectrum of (*RR/SS*)-*N*,*N*′-bis(3-bromo-5-chlorosalicylidene)-1,2-diphenyl-1,2-diaminoethane (**L3b**): $\bar{\nu}$ =3059 w; (C=N) 1623 s; 1599 w; 1443 s; 1368 m; 1291 m; 1214 m; 1170 s; 1039 m; 864 s; 775 m; 700 ss; 511 m.

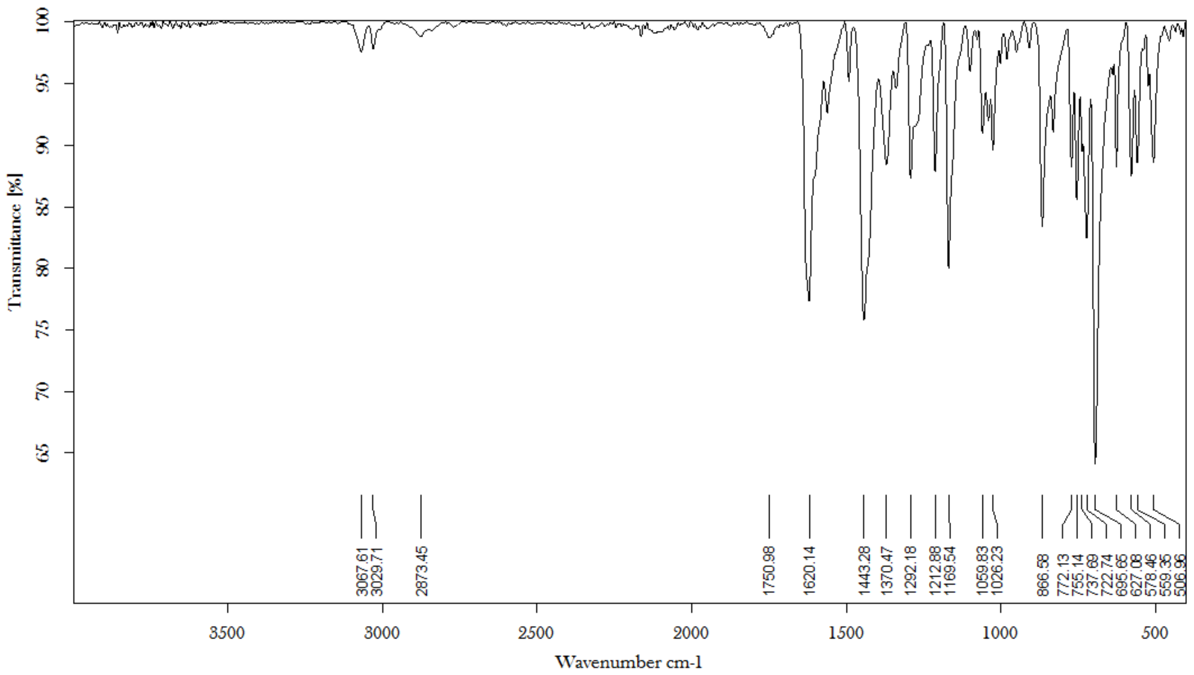

Figure S27: FT-IR spectrum of *(SS)-N,N′*-bis(3-bromo-5-chlorosalicylidene)-1,2-diphenyl-1,2-diaminoethane (**L3c**): $\bar{\nu}$ =3067 w; (C=N) 1620 s; 1443 s; 1370 m; 1292 m; 1169 s; 1059 m; 1026 m; 772 m; 695 ss; 722 m; 506 m.

FT-IR spectra of the complexes

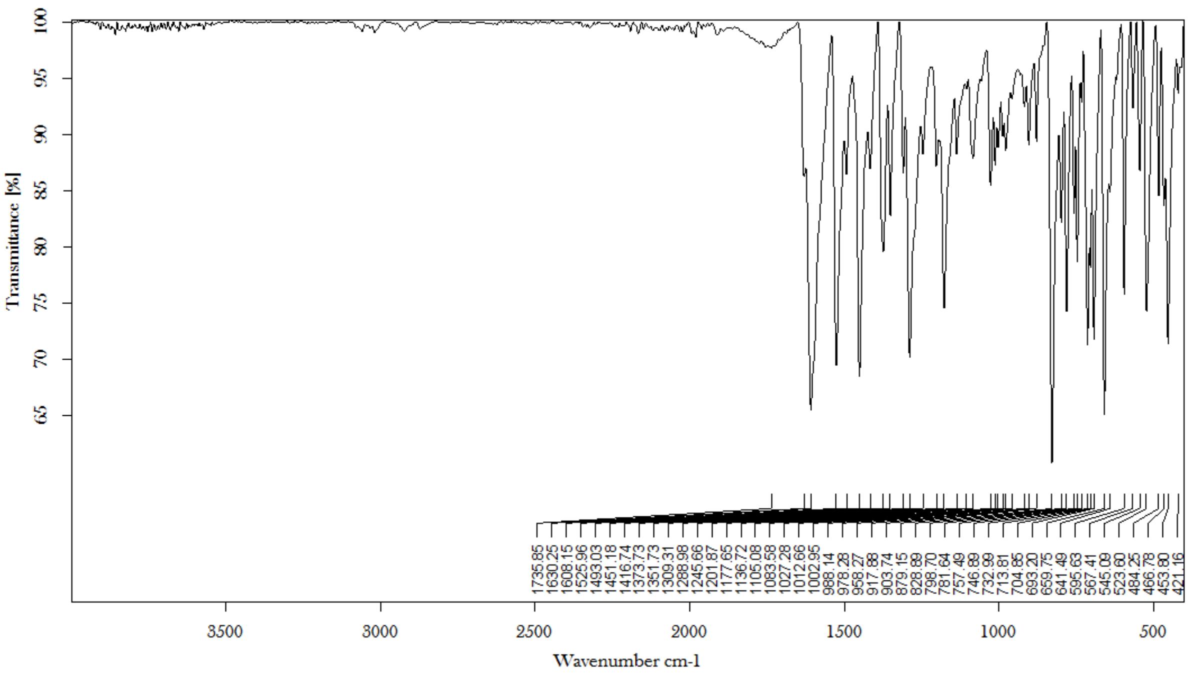

Figure S28: FT-IR spectrum of chlorido[*(RS)*-*N,N'*-bis(5-chlorosalicylidene)-1,2-diphenyl-1,2-diaminoethane]iron(III) (**1a**): $\bar{\nu}$ = 1735 w; 1630 s; 1608 s; (N=C) 1525 s; 1373 m; 1245 m; 1201 m; (C-O) 1177 m; 828 s; 798 s; 659 s; 545 m; 484 m.

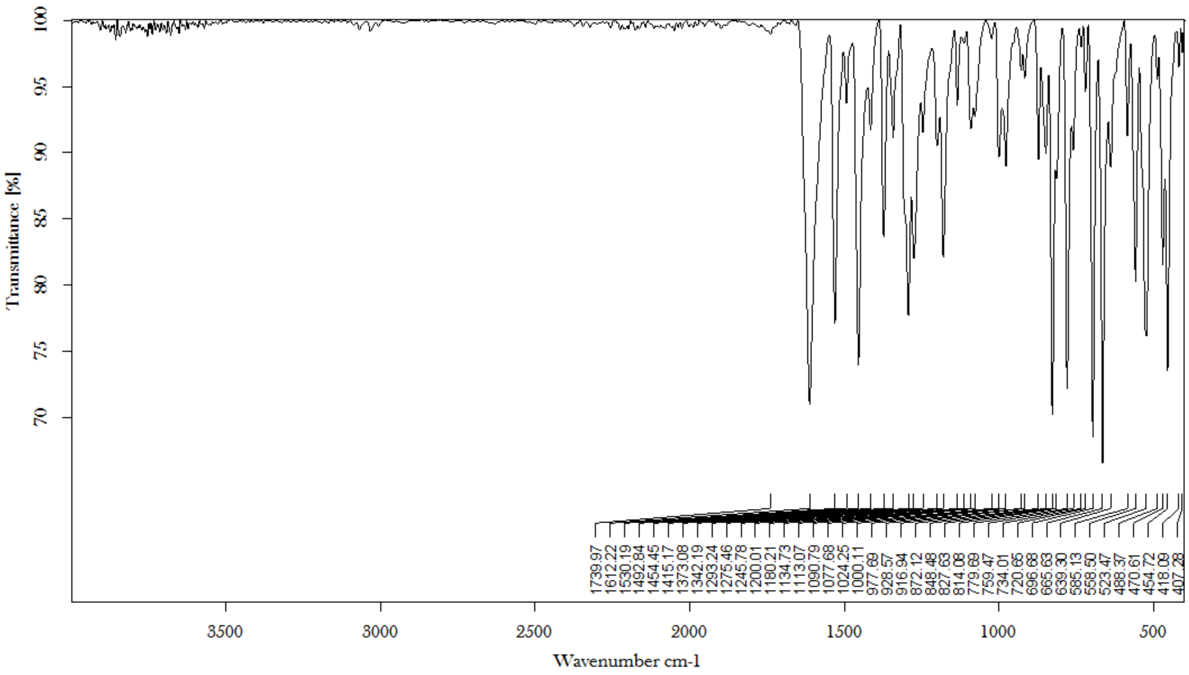

Figure S29: FT-IR spectrum of chlorido[*(RR/SS)*-*N,N'*-bis(5-chlorosalicylidene)-1,2-diphenyl-1,2-diaminoethane]iron(III) (**1b**): $\bar{\nu}$ = 1739 w; 1612 s; (N=C) 1530 m; 1373 m; 1293 m; 1200 w; (C-O) 1180 s; 827 s; 779 s; 665 ss; 523 s; 454 s.

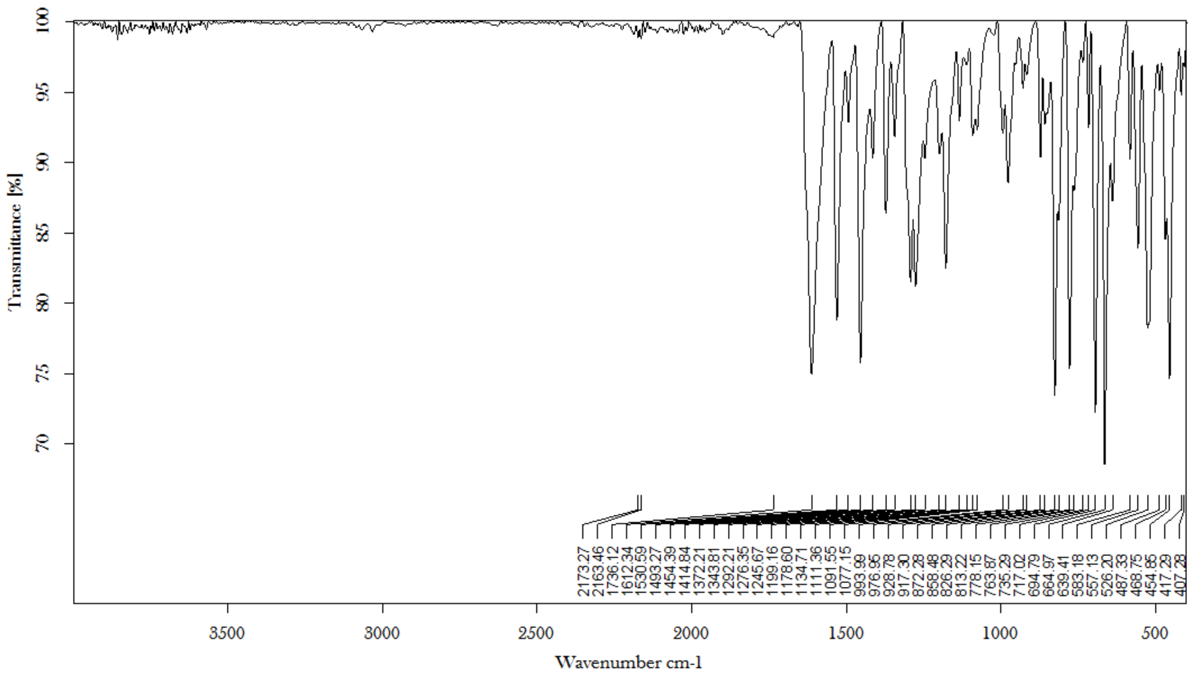

Figure S30: FT-IR spectrum of chlorido[*(SS)*-*N,N'*-bis(5-chlorosalicylidene)-1,2-diphenyl-1,2-diaminoethane]iron(III) (**1c**): $\bar{\nu}$ = 2173 w; 1612 s; (N=C) 1530 m; 1372 m; 1292 m; 1276 m; (C-O) 1178 s; 826 s; 778 s; 664 ss; 526 s; 454 s.

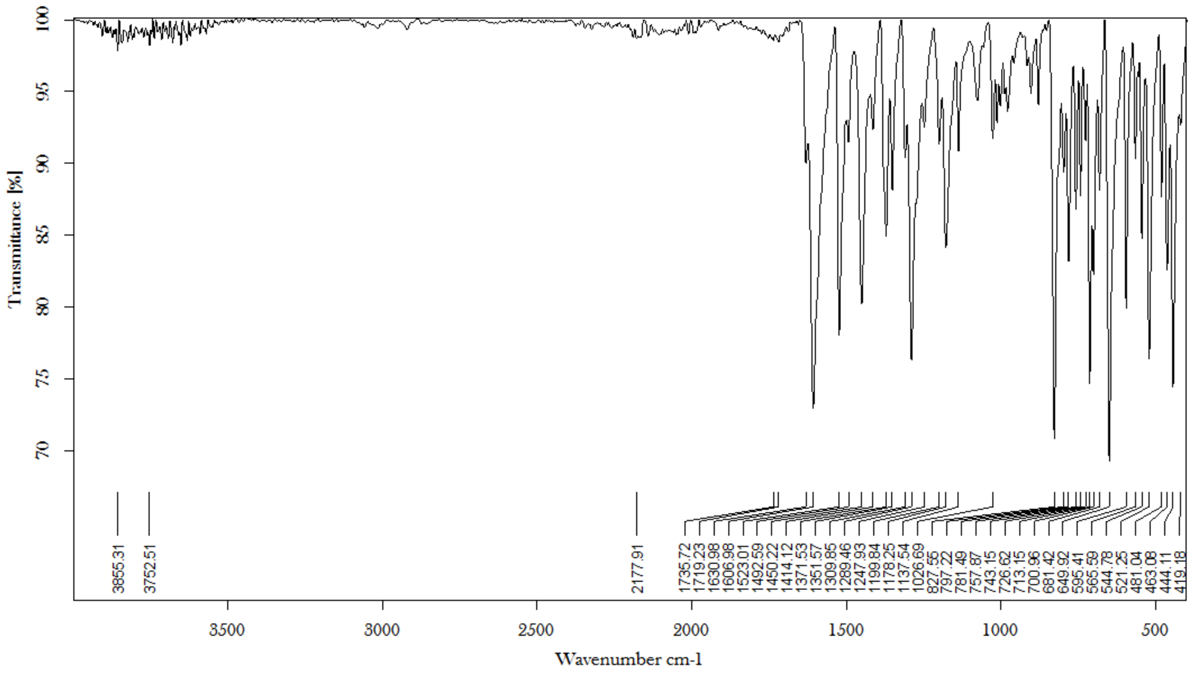

Figure S31: FT-IR spectrum of chlorido[*(RS)*-*N,N'*-bis(5-bromosalicylidene)-1,2-diphenyl-1,2-diaminoethane]iron(III) (**2a**): $\bar{\nu}$ = 3855 w; 1606 s; (N=C) 1523 m; 1371 m; 1351 w; 1289 s; 1199 w; (C-O) 1178 m; 827 s; 781m; 649 ss; 521 s; 444 s.

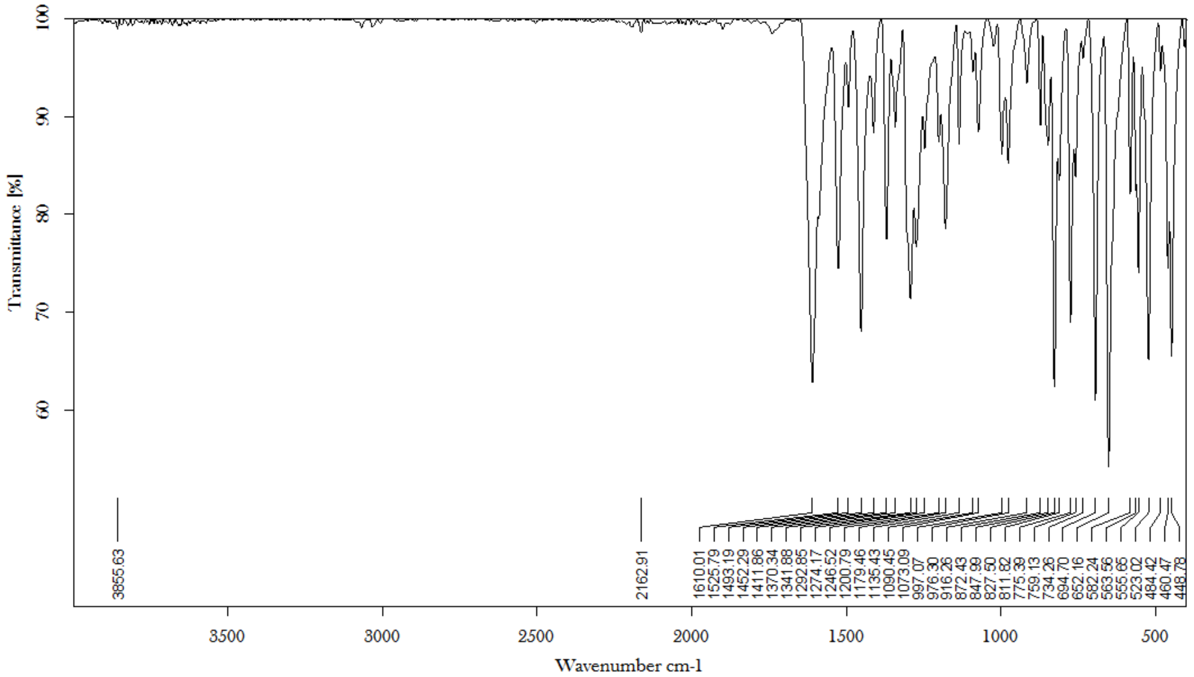

Figure S32: FT-IR spectrum of chlorido[*(RR/SS)-N,N'*-bis(5-bromosalicylidene)-1,2-diphenyl-1,2-diaminoethane]iron(III) (**2b**): $\bar{\nu}$ = 3855 w; 1610 s; (N=C) 1525 m; 1370 m; 1292 s; 1200 w; (C-O) 1179 m; 827 s; 775 m; 694 s; 652 ss; 523 s; 448 s.

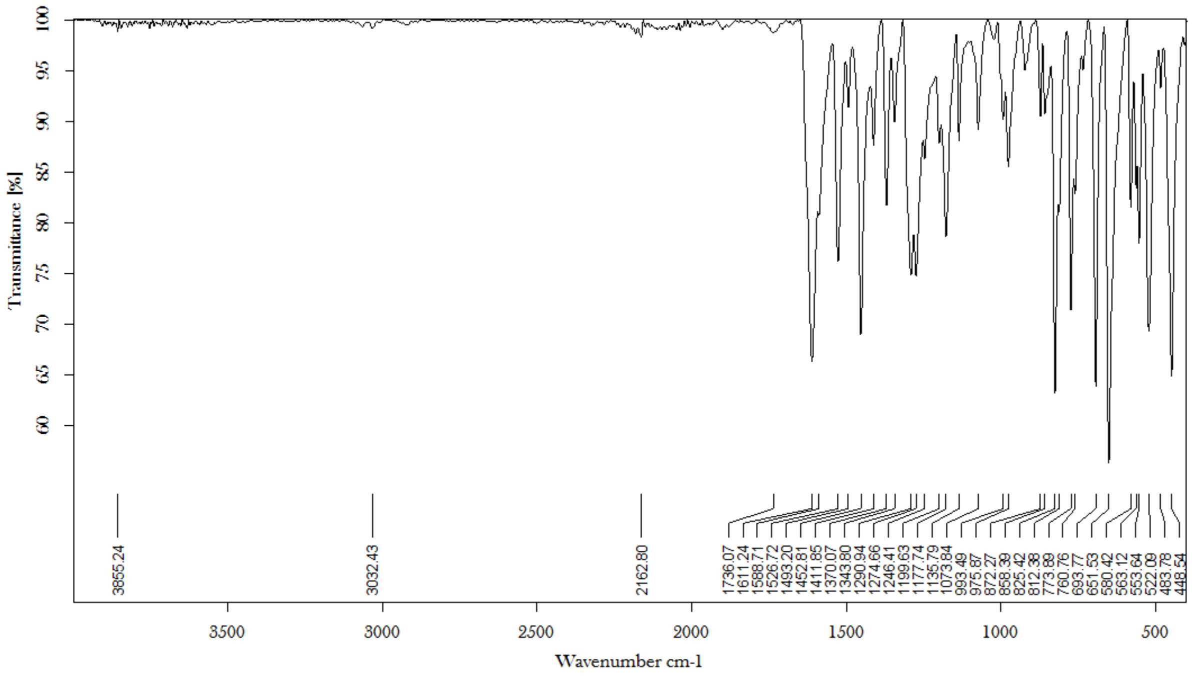

Figure S33: FT-IR spectrum of chlorido[*(SS)-N,N'*-bis(5-bromosalicylidene)-1,2-diphenyl-1,2-diaminoethane]iron(III) (**2c**): $\bar{\nu}$ = 3032 w; 1611 s; (N=C) 1526 m; 1370 m; 1290 m; 1199 w; (C-O) 1177 m; 825 s; 773 m; 651 ss; 522 s; 448 s.

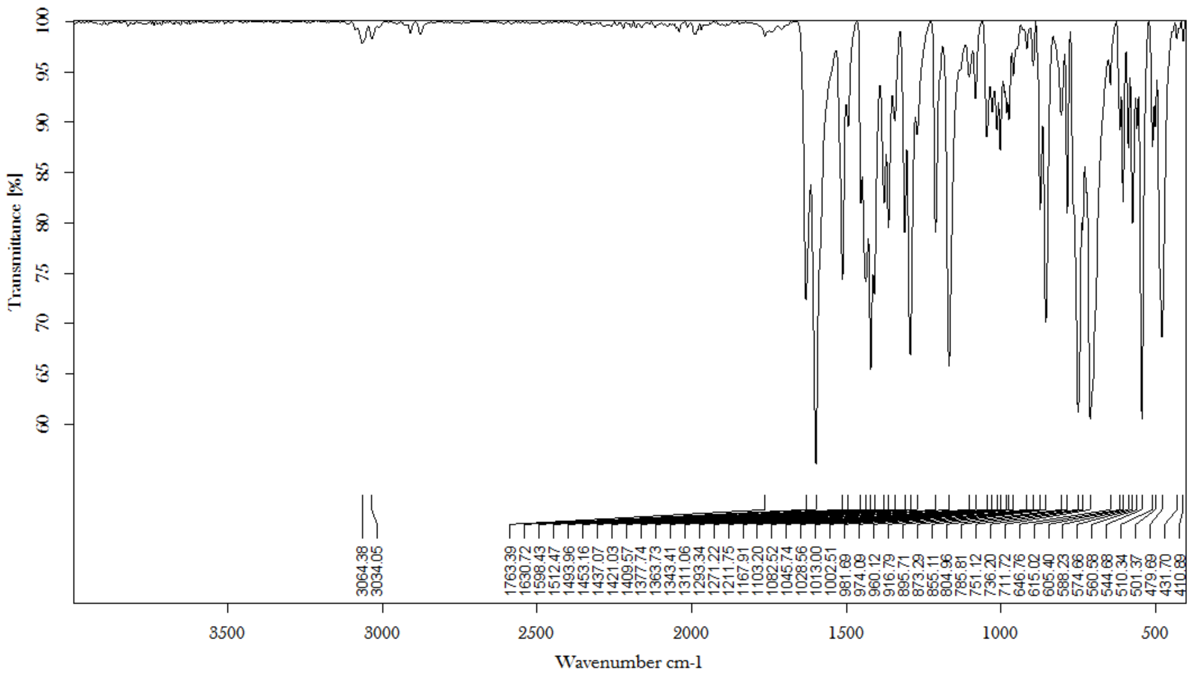

Figure S34: FT-IR spectrum of chlorido[*(RS)-N,N′*-bis(3-bromo-5-chlorosalicylidene)-1,2-diphenyl-1,2-diaminoethane]iron(III) (**3a**): $\bar{\nu}$ = 3064 w; 1598 s; (N=C) 1512 m; 1421 s; 1377 w; 1293 m; 1211 m; (C-O) 1167 s; 855 s; 751 s; 711 s; 479 s.

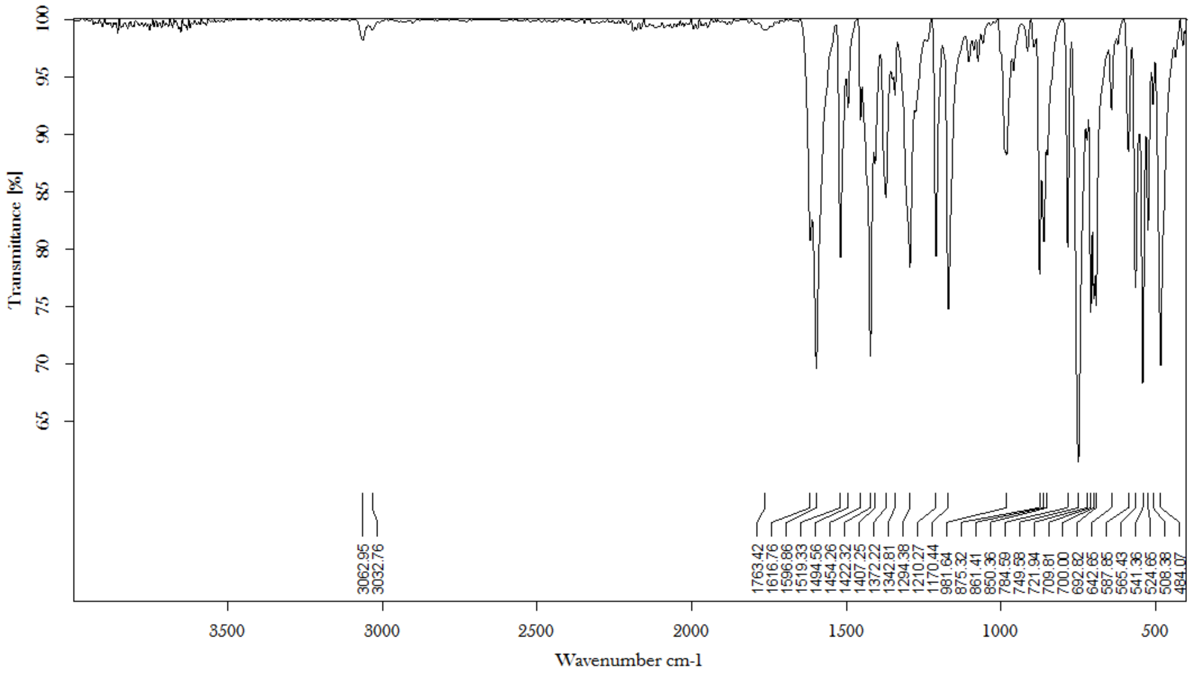

Figure S35: FT-IR spectrum of chlorido[*(RR/SS)-N,N′*-bis(3-bromo-5-chlorosalicylidene)-1,2-diphenyl-1,2-diaminoethane]iron(III) (**3b**): $\bar{\nu}$ =3062 w; 1596 s; (N=C) 1519 m; 1422 s; 1372 w; 1210 m; (C-O) 1170 s; 861 m; 749 ss; 692 m; 541 s; 484 s.

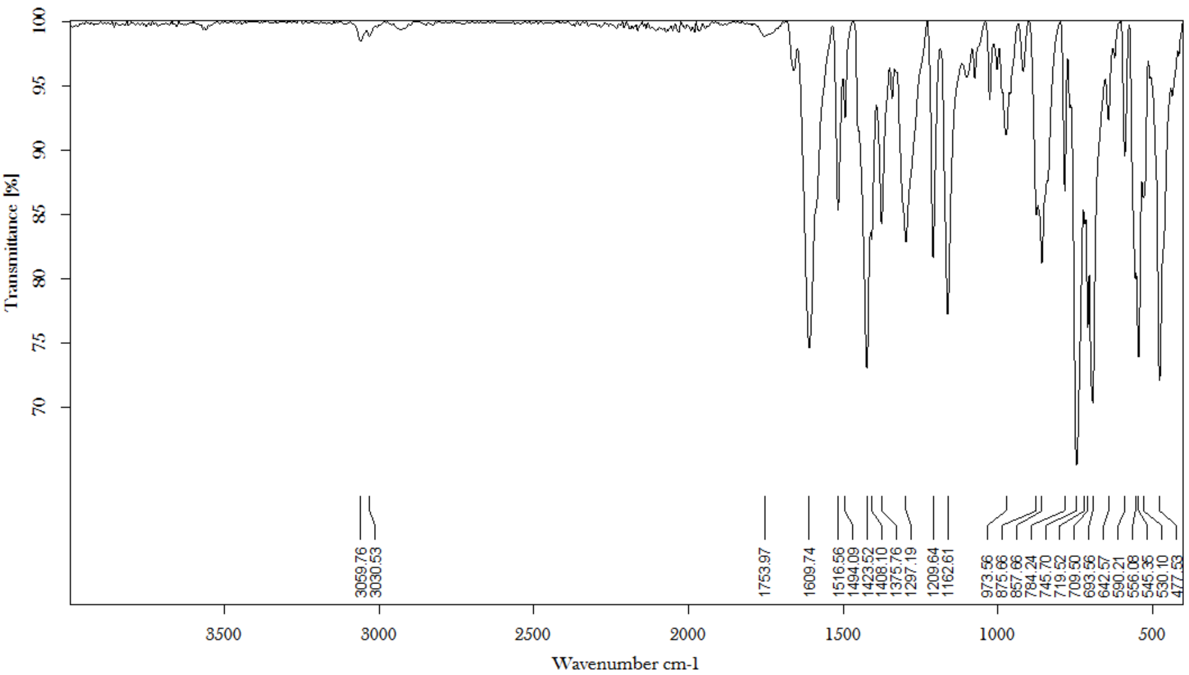

Figure S36: FT-IR spectrum of chlorido[*(SS)-N,N'*-bis(3-bromo-5-chlorosalicylidene)-1,2-diphenyl-1,2-diaminoethane]iron(III) (**3c**): $\bar{\nu}$ = 3059 w; 1609 s; (N=C) 1516 m; 1423 s; 1375 w; 1297 m; 1209 m; (C-O) 1162 s; 857 m; 745 ss; 693 s; 477 s.

ECD spectra and optical rotation

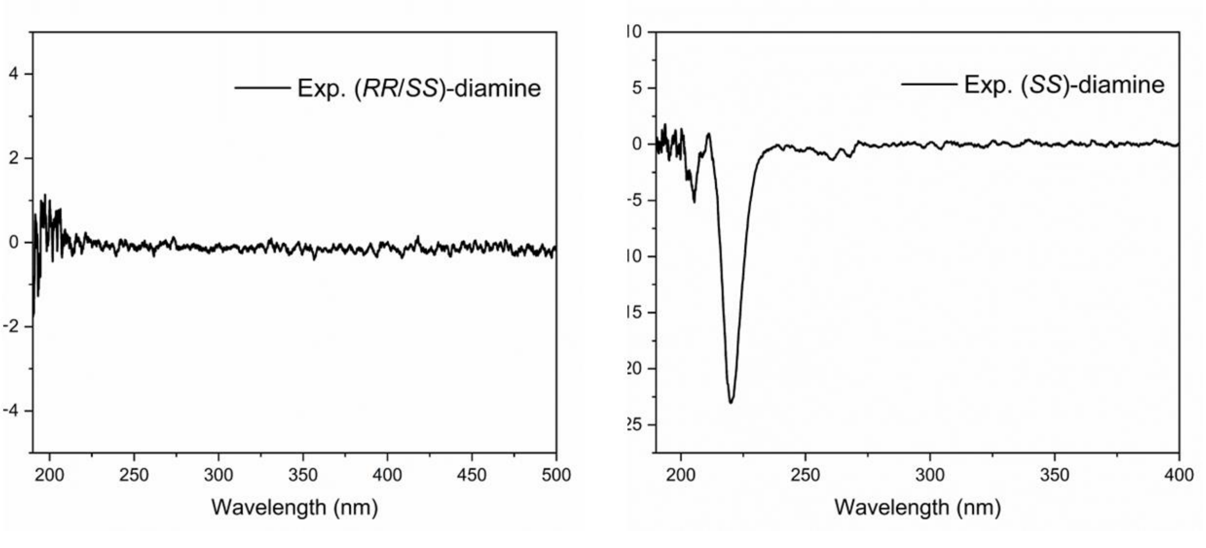

Optical rotation of (*SS*)-1,2-diphenyl-1,2-diaminoethane (SS-diamine)

$[\alpha]_D^{25}$ -447.4 (*c* 0.02 MeOH), ECD (c= 2.82 mM, MeOH): λ (mdeg) 206 (-4.47), 220 (-22.9)

Figure S37: ECD spectra of (*SS*)- and (*RR*/*SS*)-1,2-diphenyl-1,2-diaminoethane and optical rotation of (*SS*)-1,2-diphenyl-1,2-diaminoethane.

HPLC data of the complexes

Table S1: HPLC data of **1a-c** to **3a-c** dissolved in MeOH

Compound	Retention ti (Rt) [min]	Area under the curve (AUC) [%]
**blank**	2.75	-
**1a**	8.76	100
**1b**	8.79	96.8
**1c**	8.75	98.9
**2a**	10.77	100
**2b**	10.79	100
**2c**	10.75	99.2
**3a**	9.83	100
**3b**	24.93	100
**3c**	20.70	98.8

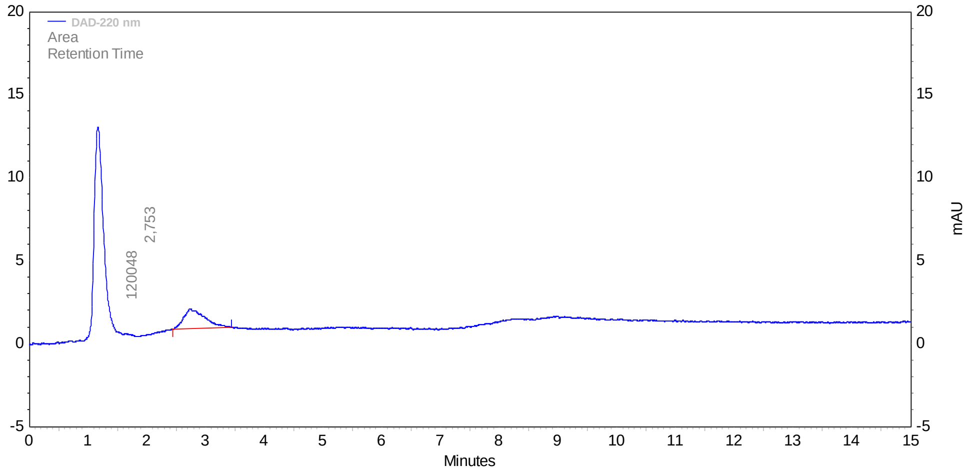

Figure S38: HPLC chromatogram of the solvent (MeOH).

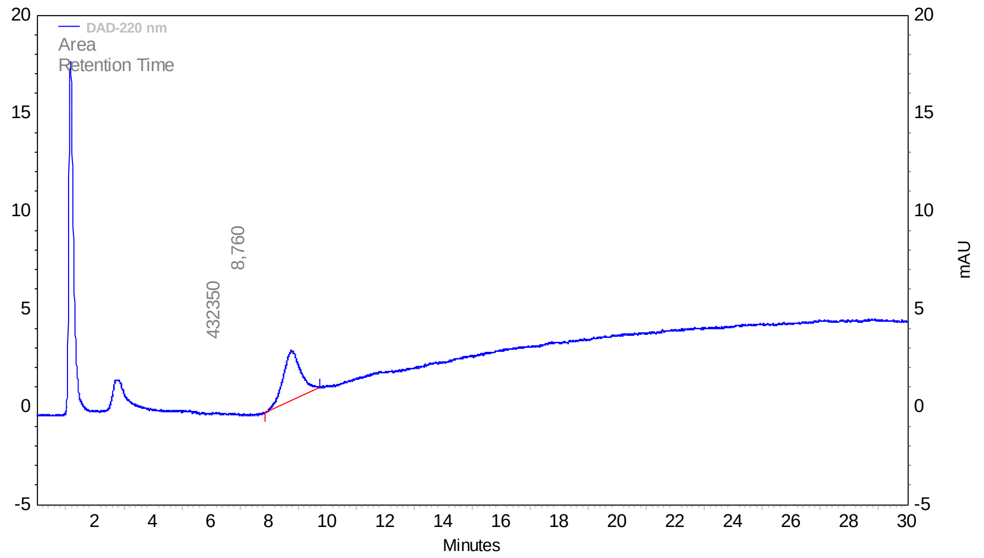

Figure S39: HPLC chromatogram of **1a** in MeOH.

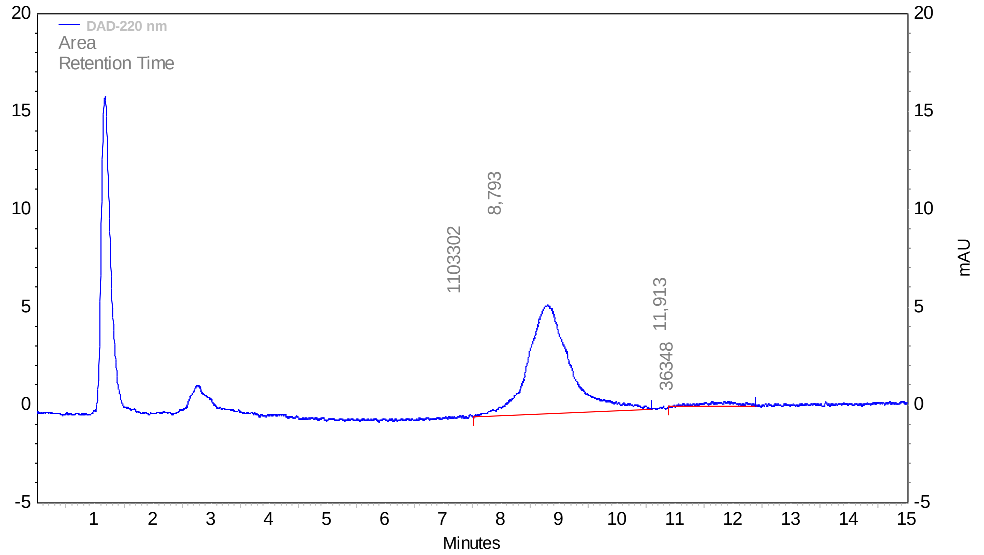

Figure S40: HPLC chromatogram of **1b** in MeOH.

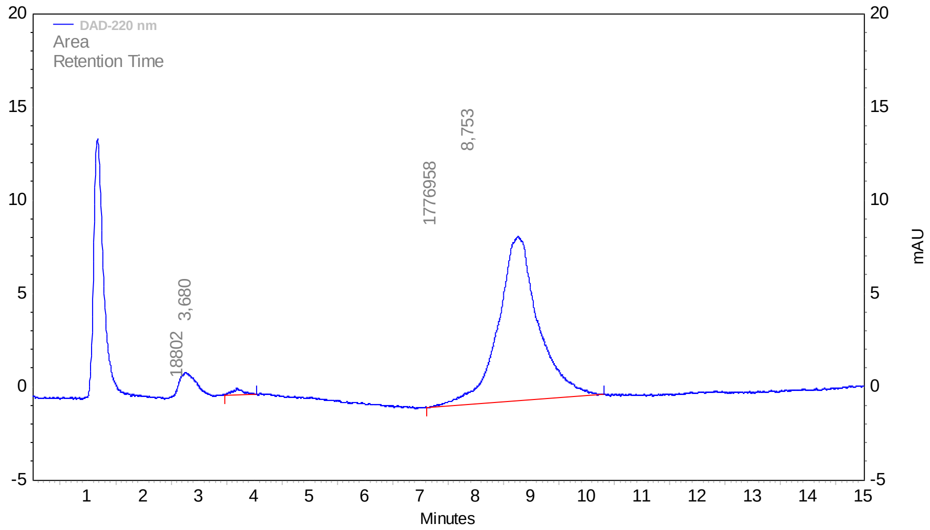

Figure S41: HPLC chromatogram of **1c** in MeOH.

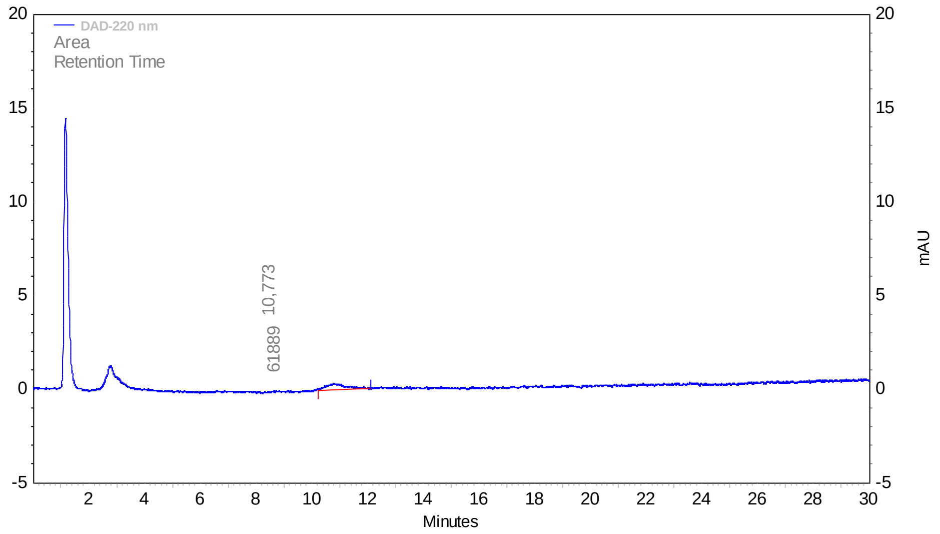

Figure S42: HPLC chromatogram of **2a** in MeOH.

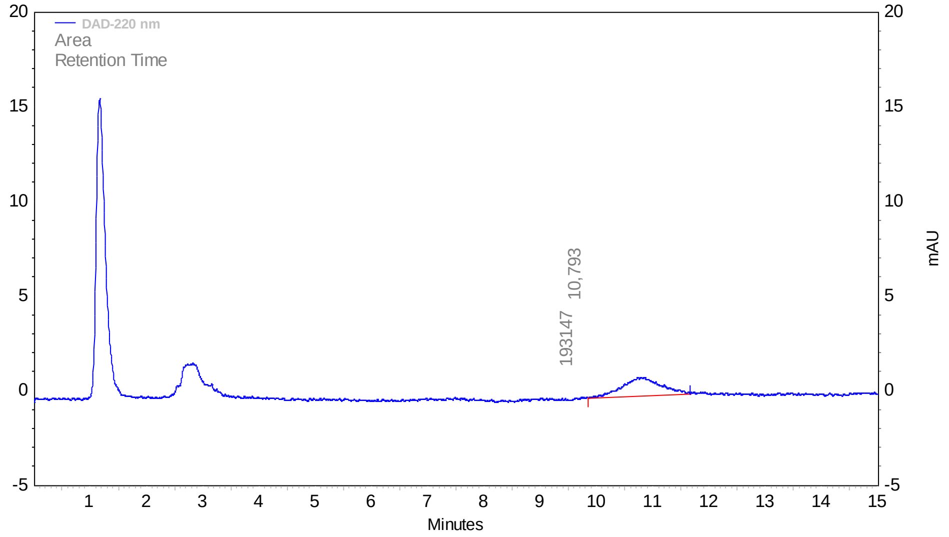

Figure S43: HPLC chromatogram of **2b** in MeOH.

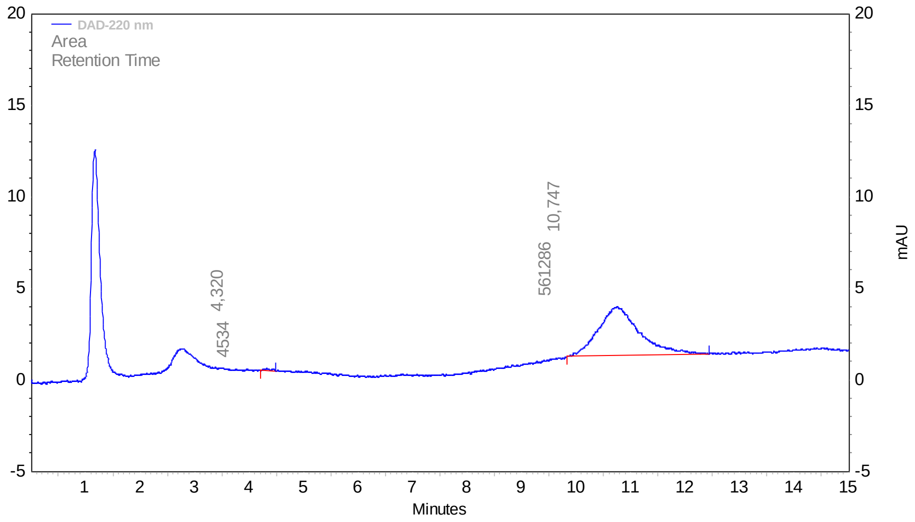

Figure S44: HPLC chromatogram of **2c** in MeOH.

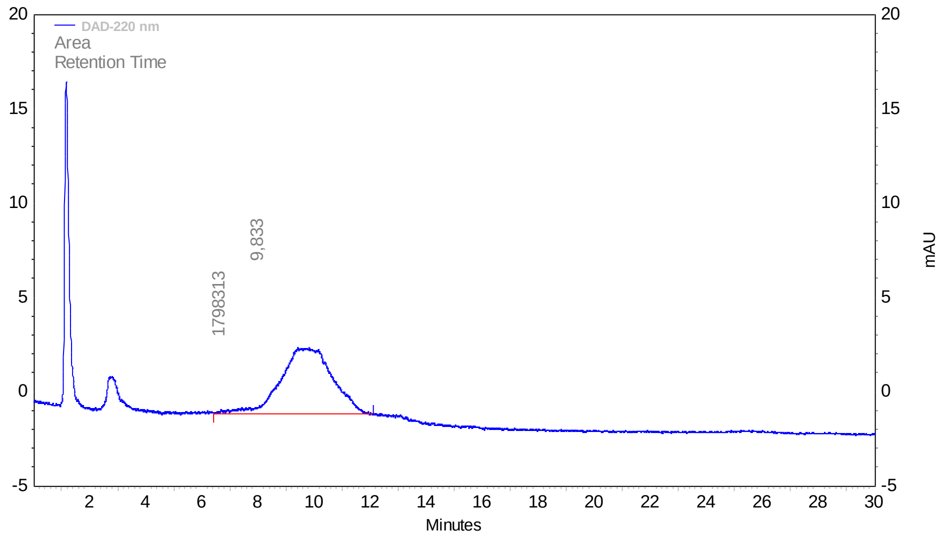

Figure S45: HPLC chromatogram of **3a** in MeOH.

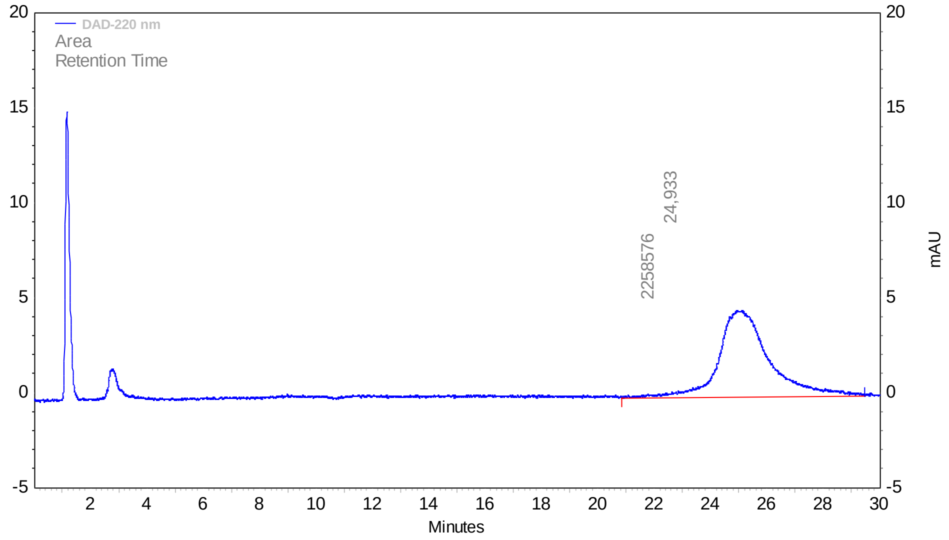

Figure S46: HPLC chromatogram of **3b** in MeOH.

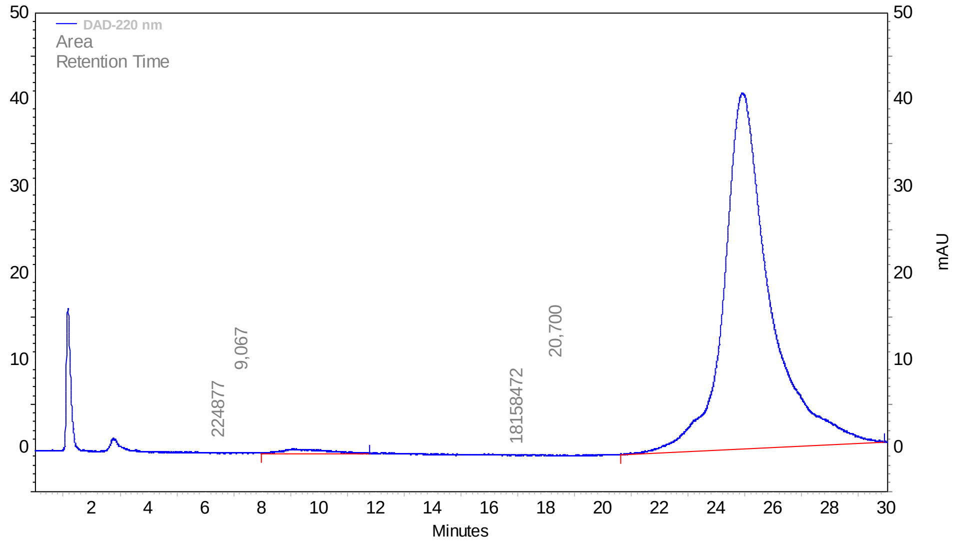

Figure S47: HPLC chromatogram of **3c** in MeOH.

EPR spectra of the complexes

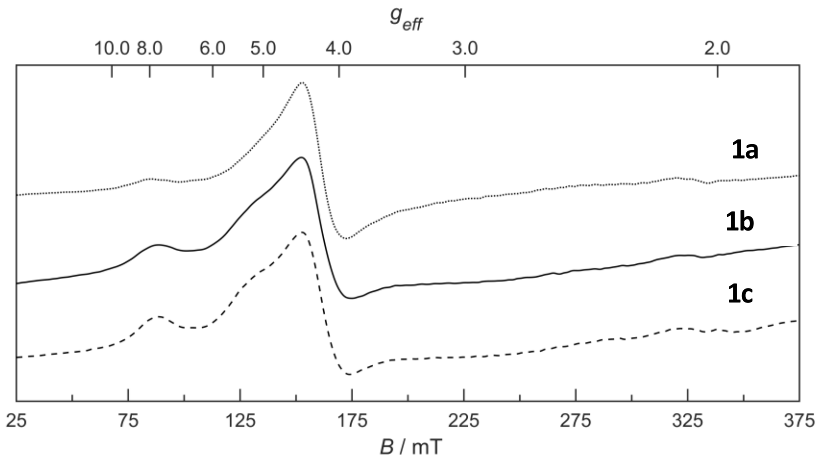

Figure S48: EPR spectra of **1a-c** in DMSO at 98 K.

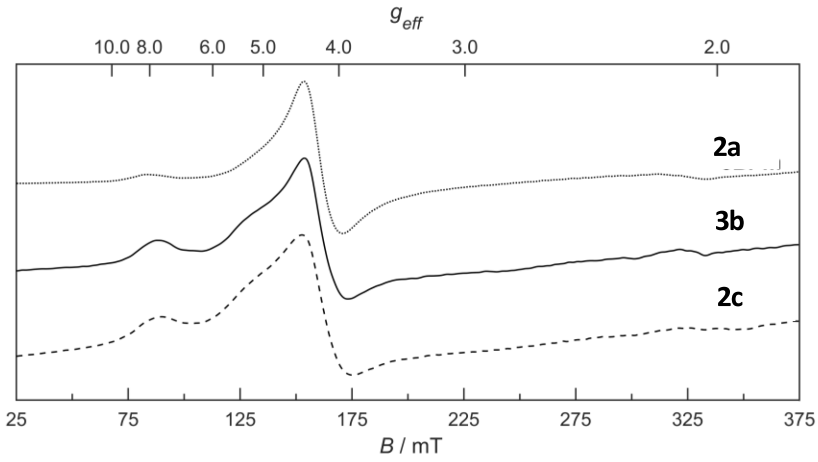

Figure S49: EPR spectra of **2a-c** in DMSO at 98 K.

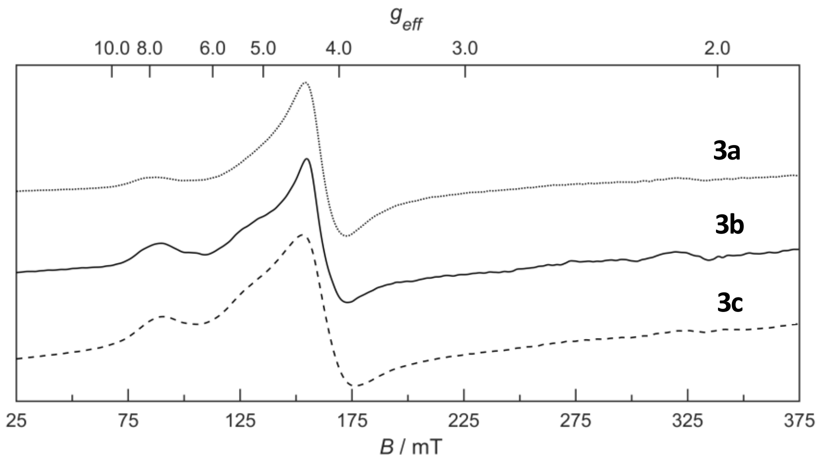

Figure S50: EPR spectra of **3a-c** in DMSO at 98 K.

Crystallographic details

Complex **1a_DCM:**

Dark brown block shaped crystals of the complex **1a_DCM** were obtained from a solution of **1a** in DCM by slow evaporation of the solvent at -30 °C. The complex **1a_DCM** crystalizes in the space group $P2_1/n$ with one molecule of **1a** and a DCM solvent molecule in the asymmetric unit.

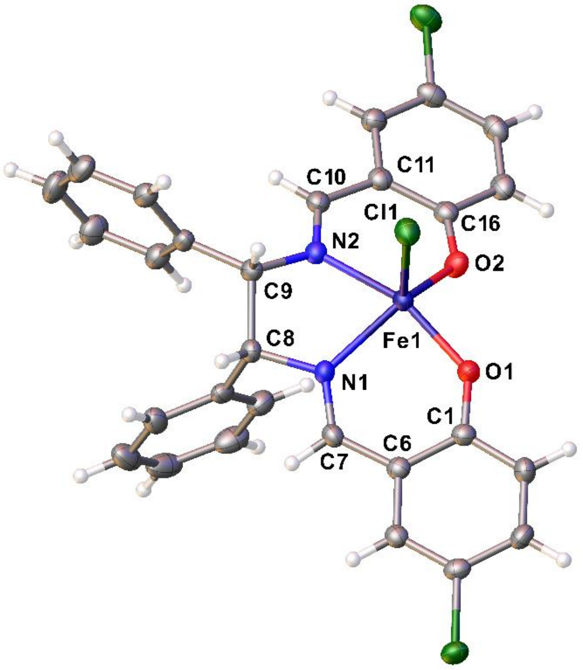

Figure S51: Molecular structure of complex **1a_DCM**. Thermal ellipsoids are set at 50 % probability level. The DCM solvent molecule is omitted for clarity.

Table S2: Selected bond lengths [Å] and bond angles [°] for complex **1a_DCM**.

Bond Length [Å]		Bond Angles [°]	
Fe1–O1	1.8915(18)	O1–Fe1–O2	92.93(8)
Fe1–O2	1.8900(18)	N1–Fe1–N2	77.61(8)
Fe1–Cl1	2.2470(7)	N1–Fe1–O1	88.16(8)
Fe1–N1	2.071(2)	N2–Fe1–O2	86.92(8)
Fe1–N2	2.102(2)	Cl1–Fe1–O1	103.98(6)
		Cl1–Fe1–O2	112.83(6)
		Cl1–Fe1–N1	105.78(6)
		Cl1–Fe1–N2	96.97(6)

Table S3: Crystal data and structure refinement for **1a_DCM**.

Compound	1a_DCM
Identification code	ABS26_06_m_Cl_DCM
CCDC number	2532650
Formula	$C_{29}H_{22}Cl_5FeN_2O_2$
$D_{calc.}$/ g cm^{-3}	1.557
μ/mm^{-1}	1.036
Formula Weight	663.58
Colour	dark brown
Shape	block-shaped
Size/mm	0.18×0.14×0.03
T/K	153.00
Crystal System	monoclinic
Space Group	$P2_1/n$
a/Å	13.7071(5)
b/Å	12.5326(4)
c/Å	17.4641(7)
α/°	90
β/°	109.3840(10)
γ/°	90
V/Å^3	2830.02(18)
Z	4
Z'	1
Wavelength/Å	0.71073
Radiation type	MoK[illegible]
Θ_{min}/°	2.042
Θ_{max}/°	26.732
Index range h	-17 ≥ h ≥1 7
Index range k	-15 ≥ k ≥ 15
Index range l	-21 ≥ l ≥ 22
Measured Refl's.	53420
Indep't Refl's	5997
Refl's I≥2*s*(I)	5293
R_{int}	0.0471
Parameters	352
Restraints	0
Largest Peak/eÅ^3	0.610
Deepest Hole/eÅ^3	-0.289
GooF	1.097
R_1 (I≥2*s*(I) / all)	0.0381 / 0.0441
wR_2 (I≥2*s*(I) / all)	0.1015 / 0.1052

Complex **1a_DMSO:**

Dark orange block shaped crystals of the complex **1a_DMSO** were obtained from a solution of **1a** in MeOH and DMSO by slow evaporation of the solvents. The complex **1a_DMSO** crystalizes in the space group *C*2/*c* with half a molecule of **1a** and one DMSO molecule in the asymmetric unit. The DMSO molecule is disordered over two positions (occupancy 79:21).

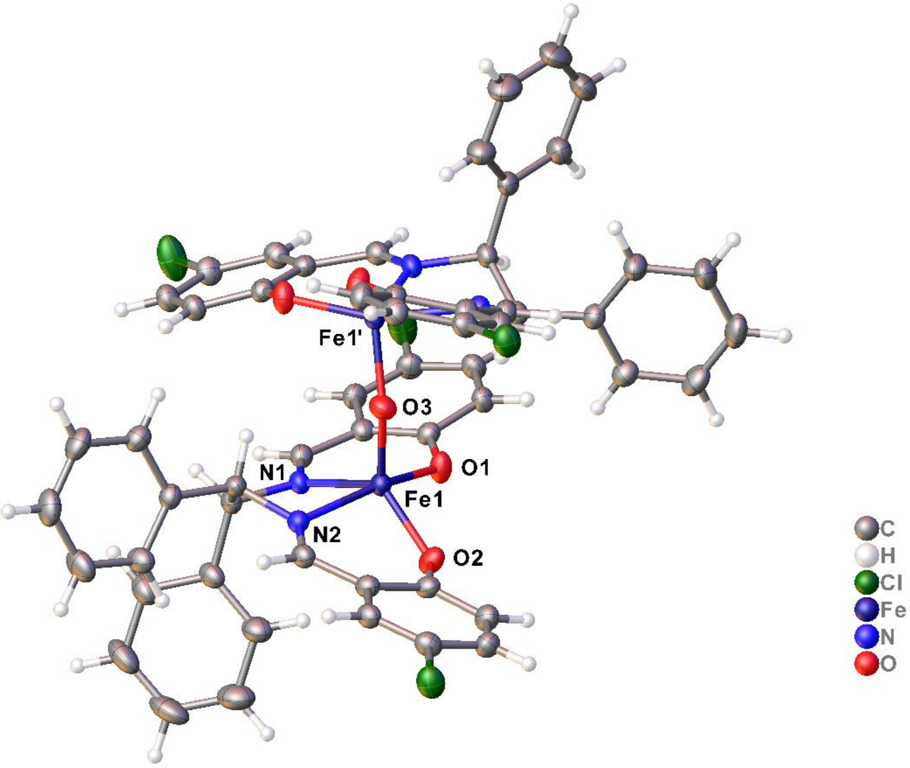

Figure S52: Molecular structure of complex **1a_DMSO**. Thermal ellipsoids are set at 50 % probability level. The DMSO solvent molecule is omitted for clarity.

Table S4: Selected bond lengths [Å] and bond angles [°] for complex **1a_DMSO**.

**Bond Length [Å]**		**Bond Angles [°]**	
Fe1–O1	1.9279(14)	Fe1–O3–Fe1’	153.95(15)
Fe1–O2	1.9024(15)	O1–Fe1–O2	95.09(6)
Fe1–O3	1.7708(6)	N1–Fe1–N2	77.43(6)
Fe1–N1	2.1054(17)	N1–Fe1–O1	87.00(6)
Fe1–N2	2.1065(17)	N2–Fe1–O2	86.62(6)

Symmetry code: (‘) = 1-x, +y, 3/2-z

Table S5: Crystal data and structure refinement for **1a_DMSO.**

**Compound**	**1a_DMSO**
Identification code	ABS26_01_m_Cl_DMSO
CCDC number	2532651
Formula	$C_{60}H_{52}N_4O_7S_2Cl_4Fe_2$
$D_{calc.}$/ g cm^{-3}	1.482
μ/mm^{-1}	0.836
Formula Weight	1258.67
Colour	clear dark orange
Shape	block-shaped
Size/mm	0.19×0.06×0.05
T/K	153.00
Crystal System	monoclinic
Space Group	*C*2/*c*
a/Å	15.9994(5)
b/Å	20.1485(6)
c/Å	17.5881(4)
α/°	90
β/°	95.7410(10)
γ/°	90
V/Å^3	5641.3(3)
Z	4
Z'	0.5
Wavelength/Å	0.71073
Radiation type	MoK_{α}
Θ_{min}/°	2.559
Θ_{max}/°	25.353
Index range h	-19 ≥ h ≥ 19
Index range k	-24 ≥ k ≥ 24
Index range l	-21 ≥ l ≥ 21
Measured Refl's.	42941
Indep't Refl's	5155
Refl's I≥2*s*(I)	4523
R_{int}	0.0634
Parameters	398
Restraints	80
Largest Peak/eÅ^3	0.707
Deepest Hole/eÅ^3	-0.517
GooF	1.071
R_1 (I≥2*s*(I) / all)	0.0342 / 0.0402
wR_2 (I≥2*s*(I) / all)	0.0855 / 0.0902

Complex **1b_DCM:**

Dark orange plate shaped crystals of the complex **1b_DCM** were obtained from a solution of **1b** in DCM by slow evaporation of the solvent at -30 °C. The complex **1b_DCM** crystalizes in the space group $P\overline{1}$ with one molecule of **1b** in the asymmetric unit. The CH–CH unit in the backbone is disordered over two positions (occupancy 81:19).

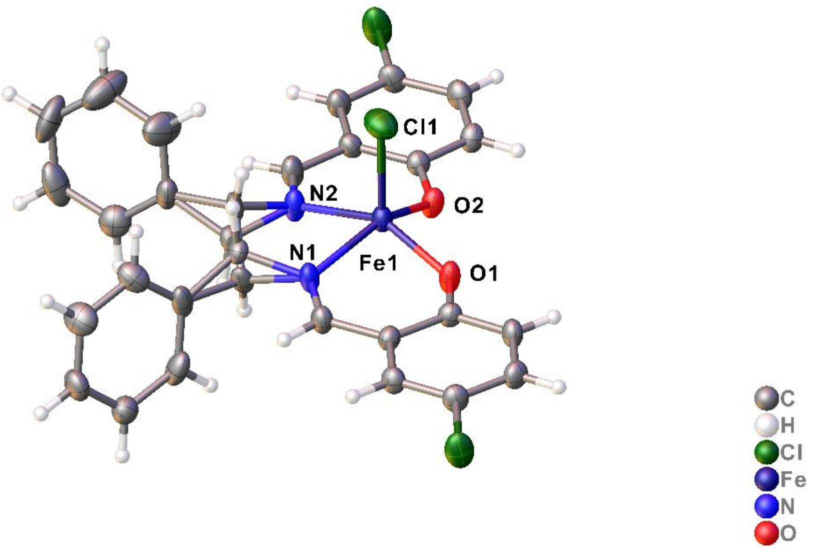

Figure S53: Molecular structure of complex **1b_DCM**. Thermal ellipsoids are set at 50 % probability level.

Table S6: Selected bond lengths [Å] and bond angles [°] for complex **1b_DCM**.

Bond Length [Å]		Bond Angles [°]	
Fe1–O1	1.8930(18)	O1–Fe1–O2	92.95(8)
Fe1–O2	1.8967(17)	N1–Fe1–N2	77.27(8)
Fe1–Cl1	2.2130(9)	N2–Fe1–O2	86.76(8)
Fe1–N1	2.090(2)	N1–Fe1–O1	87.72(8)
Fe1–N2	2.087(2)	N1–Fe1–Cl1	101.31(7)
		O1–Fe1–Cl1	107.96(7)

Table S7: Crystal data and structure refinement for **1b_DCM.**

**Compound**	**1b_DCM**
Identification code	ABS26_05_RR_SS_Cl_DCM
CCDC number	2532652
Formula	$C_{28}H_{20}Cl_3FeN_2O_2$
$D_{calc.}$/ g cm^{-3}	1.510
μ/mm^{-1}	0.937
Formula Weight	578.66
Colour	clear dark orange
Shape	plate-shaped
Size/mm	0.07×0.04×0.02
T/K	153.00
Crystal System	triclinic
Space Group	$P\bar{1}$
a/Å	9.6132(6)
b/Å	10.4784(6)
c/Å	13.8574(8)
α/°	107.146(2)
β/°	100.523(2)
γ/°	99.902(2)
V/Å^3	1272.96(13)
Z	2
Z'	1
Wavelength/Å	0.71073
Radiation type	MoK_α
Θ_{min}/°	2.161
Θ_{max}/°	25.374
Index range h	-11 ≥ h ≥ 11
Index range k	-12 ≥ k ≥ 12
Index range l	-16 ≥ l ≥ 16
Measured Refl's.	9322
Indep't Refl's	4673
Refl's I≥2s(I)	3807
R_{int}	0.0314
Parameters	344
Restraints	13
Largest Peak/eÅ^3	0.681
Deepest Hole/eÅ^3	-0.355
GooF	1.020
R_1 (I≥2s(I) / all)	0.0381 / 0.0511
wR_2 (I≥2s(I) / all)	0.0872 / 0.0930

Complex **1c_DCM:**

Dark orange plate shaped crystals of the complex **1c_DCM** were obtained from a solution of **1c** in DCM by slow evaporation of the solvent at -30 °C. The complex **1c_DCM** crystalizes in the space group *P*1 with two molecules of **1c** in the asymmetric unit.

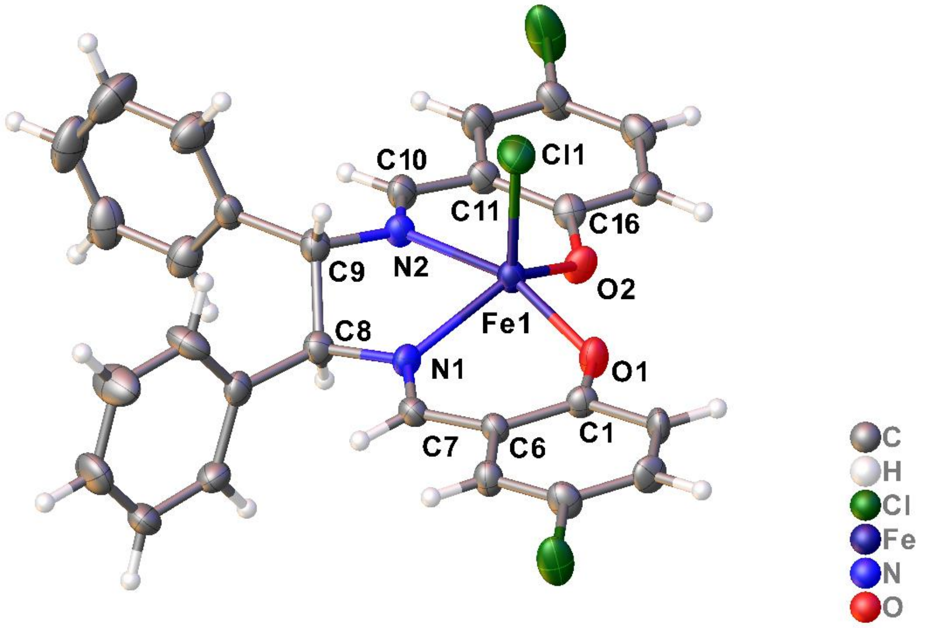

Figure S54: Molecular structure of complex **1c_DCM**. Thermal ellipsoids are set at 50 % probability level. Second molecule in the asymmetric unit was omitted for clarity.

Table S8: Selected bond lengths [Å] and bond angles [°] for complex **1c_DCM**.

Bond Length [Å]		Bond Angles [°]	
Fe1–O1	1.896(4)	O1–Fe1–O2	94.5(2)
Fe1–O2	1.875(5)	N1–Fe1–N2	77.3(2)
Fe1–Cl1	2.2213(19)	N2–Fe1–O2	87.3(2)
Fe1–N1	2.093(6)	N1–Fe1–O1	87.7(2)
Fe1–N2	2.102(5)	N1–Fe1–Cl1	108.2(2)
		O1–Fe1–Cl1	103.5(2)

Table S9: Crystal data and structure refinement for **1c_DCM.**

**Compound**	**1c_DCM**
Identification code	ABS_SS_Cl_DCM
CCDC number	2532653
Formula	$C_{28}H_{20}N_2O_2Cl_3Fe$
$D_{calc.}$/ g cm^{-3}	1.499
μ/mm^{-1}	0.930
Formula Weight	578.66
Colour	dark orange
Shape	plate-shaped
Size/mm	0.11×0.08×0.01
T/K	153.00
Crystal System	triclinic
Flack Parameter	0.006(15)
Hooft Parameter	0.003(13)
Space Group	*P*1
a/Å	9.6852(6)
b/Å	10.5793(6)
c/Å	13.8025(8)
α/°	107.760(2)
β/°	99.040(2)
γ/°	101.650(2)
V/Å^3	1282.31(13)
Z	2
Z'	2
Wavelength/Å	0.71073
Radiation type	MoK$_\alpha$
Θ_{min}/°	2.147
Θ_{max}/°	25.022
Index range h	-11 ≥ h ≥ 11
Index range k	-12 ≥ k ≥ 12
Index range l	-16 ≥ l ≥ 16
Measured Refl's.	30270
Indep't Refl's	9043
Refl's I≥2*s*(I)	7630
R_{int}	0.0658
Parameters	649
Restraints	3
Largest Peak/eÅ^3	0.431
Deepest Hole/eÅ^3	-0.319
GooF	1.038
R_1 (I≥2*s*(I) / all)	0.0387 / 0.0521
wR_2 (I≥2*s*(I) / all)	0.0841 / 0.0897

Complex **2a_DCM:**

Dark orange block shaped crystals of the complex **2a_DCM** were obtained from a solution of **2a** in DCM by slow evaporation of the solvent at -30 °C. The complex **2a_DCM** crystalizes in the space group $P2_1/c$ with one molecule of **2a** in the asymmetric unit. The Br1 atom is disordered over two positions (occupancy 0.29:0.71).

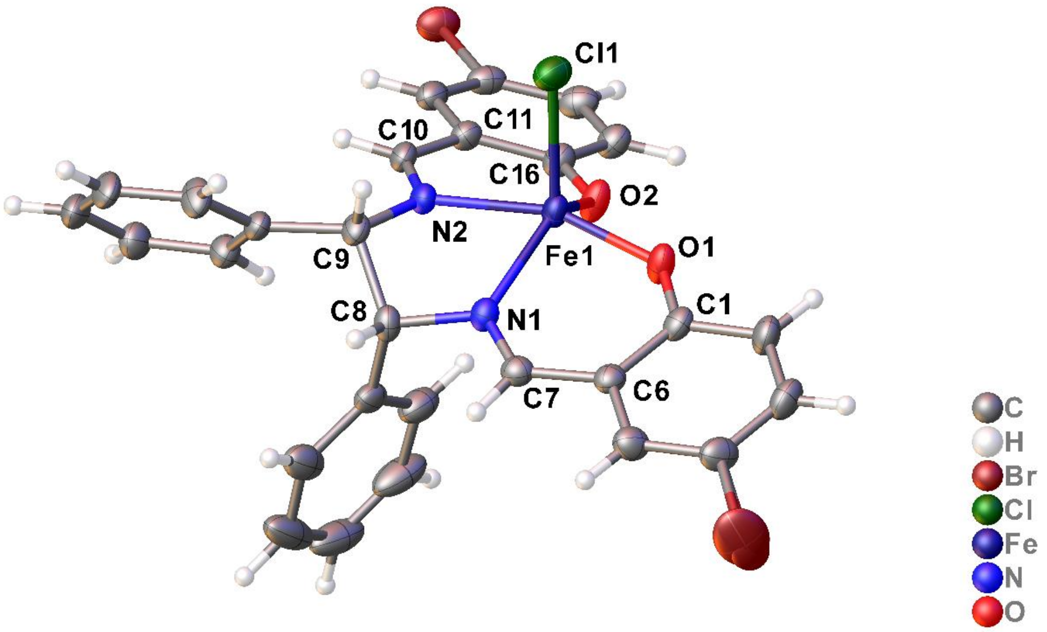

Figure S55: Molecular structure of complex **2a_DCM**. Thermal ellipsoids are set at 50 % probability level.

Table S10: Selected bond lengths [Å] and bond angles [°] for complex **2a_DCM**.

Bond Length [Å]		Bond Angles [°]	
Fe1–O1	1.897(3)	O1–Fe1–O2	97.40(12)
Fe1–O2	1.877(3)	N1–Fe1–N2	76.28(12)
Fe1–Cl1	2.2415(11)	N2–Fe1–O2	86.95(12)
Fe1–N1	2.103(3)	N1–Fe1–O1	86.41(11)
Fe1–N2	2.111(3)	N1–Fe1–Cl1	117.91(9)
		O1–Fe1–Cl1	103.57(10)

Table S11: Crystal data and structure refinement for **2a_DCM.**

Compound	2a_DCM
Identification code	ABS26_04_m_Br_DCM
CCDC number	2532654
Formula	$C_{28}H_{20}Br_2ClFeN_2O_2$
$D_{calc.}$/ g cm^{-3}	1.736
μ/mm^{-1}	3.854
Formula Weight	667.58
Colour	dark orange
Shape	block-shaped
Size/mm	0.07×0.05×0.04
T/K	153.00
Crystal System	monoclinic
Space Group	$P2_1/c$
a/Å	10.6256(5)
b/Å	11.0324(5)
c/Å	21.7892(8)
α/°	90
β/°	91.274(2)
γ/°	90
V/Å^3	2553.63(19)
Z	4
Z'	1
Wavelength/Å	0.71073
Radiation type	MoK$_\alpha$
Θ_{min}/°	2.628
Θ_{max}/°	25.027
Index range h	-12 ≥ h ≥ 12
Index range k	-13 ≥ k ≥ 13
Index range l	-25 ≥ l ≥ 25
Measured Refl's.	56155
Indep't Refl's	4507
Refl's I≥2s(I)	3966
R_{int}	0.0731
Parameters	335
Restraints	0
Largest Peak/eÅ^3	1.093
Deepest Hole/eÅ^3	-0.436
GooF	1.068
R_1 (I≥2s(I) / all)	0.0372 / 0.0439
wR_2 (I≥2s(I) / all)	0.0942 / 0.0977

Complex **3c_DMSO:**

Dark yellow needle shaped crystals of the complex **3c_DMSO** were obtained from a solution of **3c** in a mixture of MeOH and DMSO by slow evaporation of the solvents. The complex **3c_DMSO** crystalizes in the space group *C*2 with two times half a molecule of **3c** in the asymmetric unit.

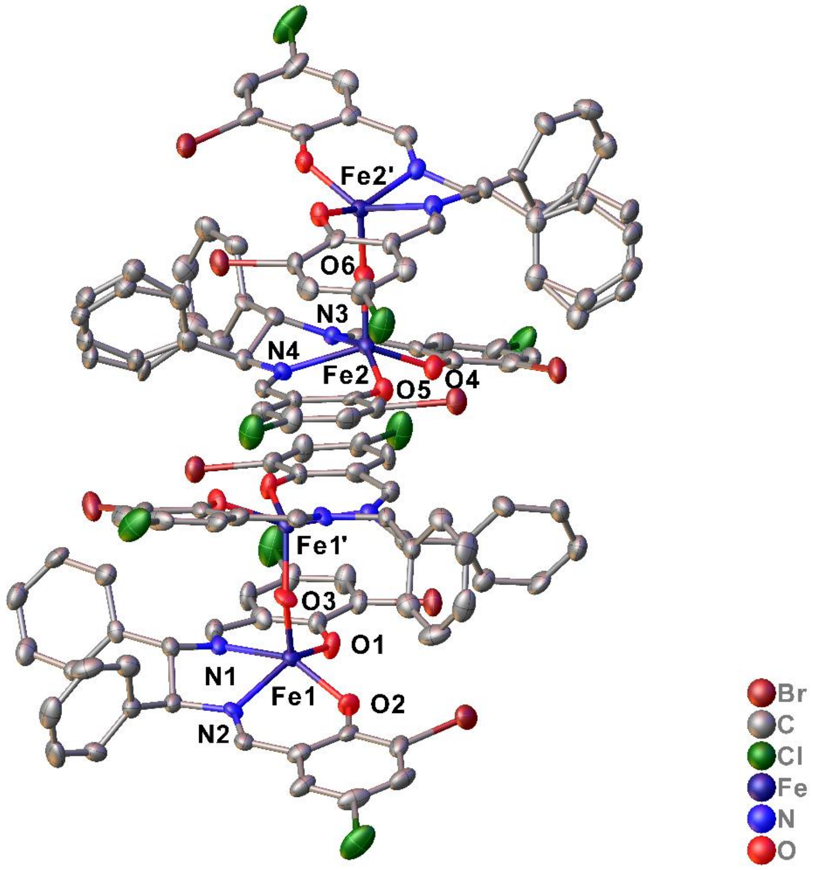

Figure S56: Molecular structure of complex **3c_DMSO**. Thermal ellipsoids are set at 50 % probability level. Both molecules in the asymmetric unit and their symmetry generated parts are shown.

Table S12: Selected bond lengths [Å] and bond angles [°] for complex **3c_DMSO**.

Bond Length [Å]		Bond Angles [°]	
Fe1–O1	1.911(9)	Fe1–O1–Fe1′	150.0(8)
Fe1–O2	1.929(9)	O1–Fe1–O2	93.3(4)
Fe1–O3	1.767(4)	N1–Fe1–N2	76.9(4)
Fe1–N1	2.078(9)	N1–Fe1–O1	87.1(4)
Fe1–N2	2.114(9)	N2–Fe1–O2	87.1(4)
Fe2–O4	1.903(9)	Fe2–O6–Fe2′	170.9(8)
Fe2–O5	1.920(9)	O4–Fe2–O5	92.1(4)
Fe2–O6	1.764(2)	N3–Fe2–N4	77.4(4)
Fe2–N3	2.101(10)	N3–Fe2–O4	86.7(4)
Fe2–N4	2.115(9)	N4–Fe2–O5	87.0(4)

Symmetry code: (′) = 1-x, +y, 1-z

Table S 13: Crystal data and structure refinement for **3c_DMSO.**

**Compound**	**3c_DMSO**
Identification code	ABS26_02_SS_Br_Cl_DMSO
CCDC number	2532655
Formula	$C_{56}H_{36}Br_4Cl_4Fe_2N_4O_5$
$D_{calc.}$/ g cm^{-3}	1.734
μ/mm^{-1}	3.727
Formula Weight	1418.03
Colour	clear dark yellow
Shape	needle-shaped
Size/mm	0.21×0.03×0.03
T/K	153.00
Crystal System	monoclinic
Flack Parameter	-0.020(14)
Hooft Parameter	-0.027(13)
Space Group	*C*2
a/Å	25.0795(16)
b/Å	13.3359(9)
c/Å	17.1747(13)
α/°	90
β/°	108.999(2)
γ/°	90
V/Å^3	5431.3(7)
Z	4
Z'	1
Wavelength/Å	0.71073
Radiation type	MoK_α
Θ_{min}/°	1.985
Θ_{max}/°	25.065
Index range h	-29 ≥ h ≥ 29
Index range k	-15 ≥ k ≥ 15
Index range l	-20 ≥ l ≥ 20
Measured Refl's.	42772
Indep't Refl's	9600
Refl's I≥2*s*(I)	7125
R_{int}	0.0933
Parameters	714
Restraints	164
Largest Peak/eÅ^3	0.540
Deepest Hole/eÅ^3	-0.567
GooF	1.020
R_1 (I≥2*s*(I) / all)	0.0427 / 0.0681
wR_2 (I≥2*s*(I) / all)	0.0837 / 0.0928

Determination of stability

Stability in PBS

HPLC analysis of the complexes 1a-c in PBS.

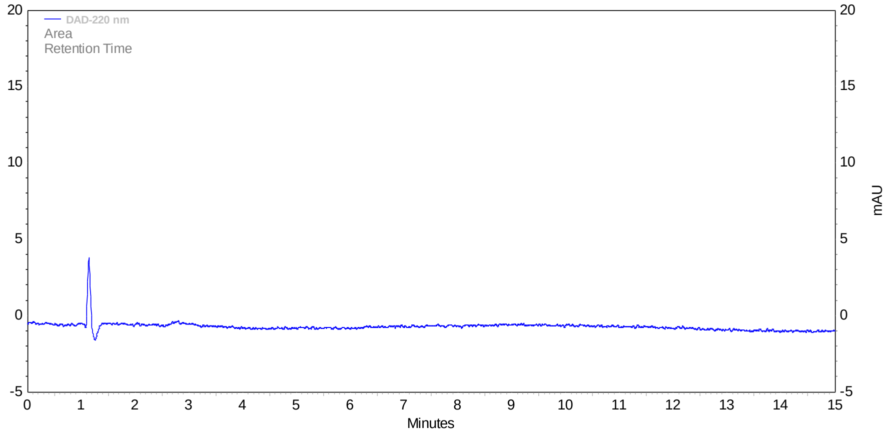

Figure S57: HPLC chromatogram of PBS incubated for 2 h at 37 °C.

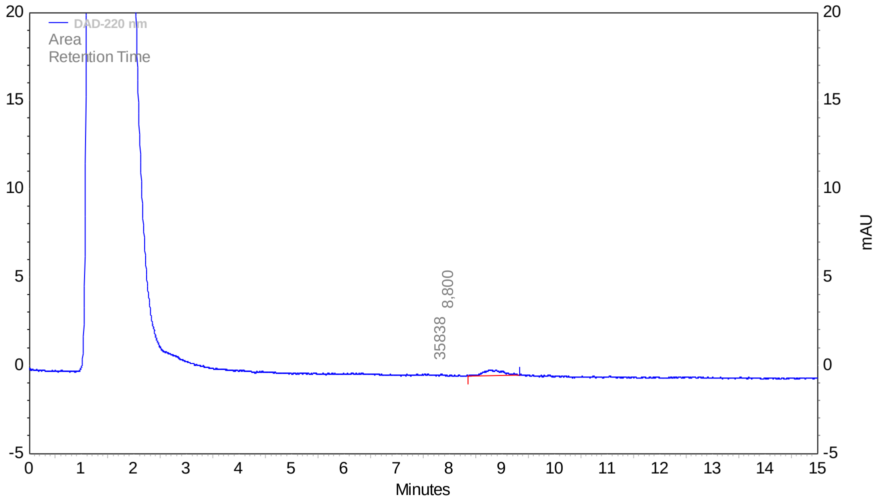

Figure S58: HPLC chromatogram of 100 µM **1a** in PBS incubated for 2 h at 37 °C.

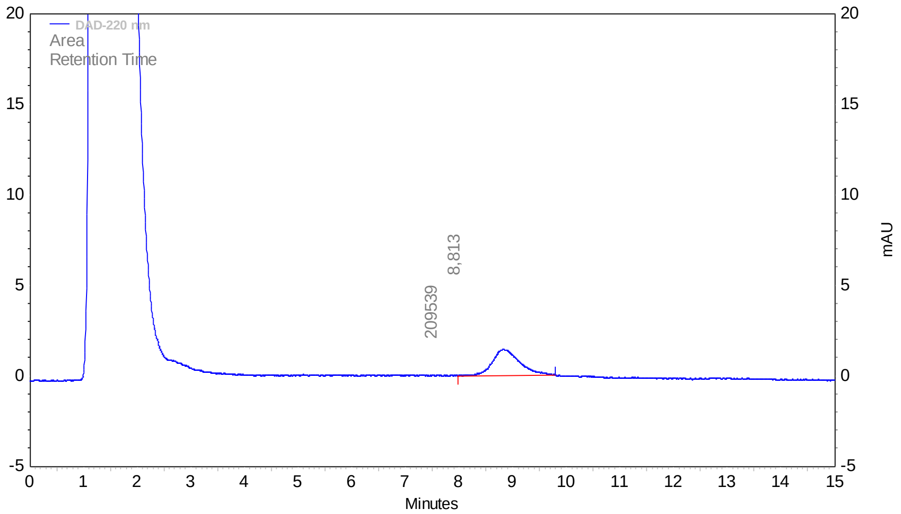

Figure S59: HPLC chromatogram of 100 µM **1b** in PBS incubated for 2 h at 37 °C.

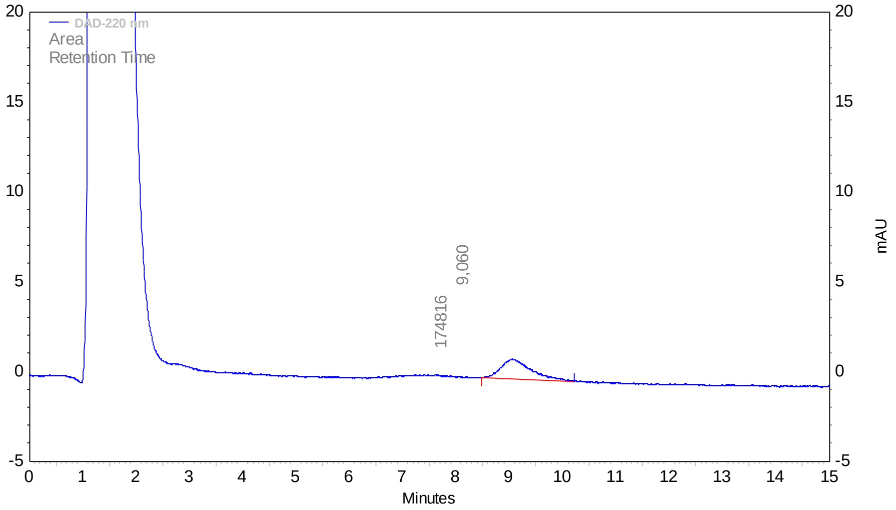

Figure S60: HPLC chromatogram of 100 µM **1c** in PBS incubated for 2 h at 37 °C.

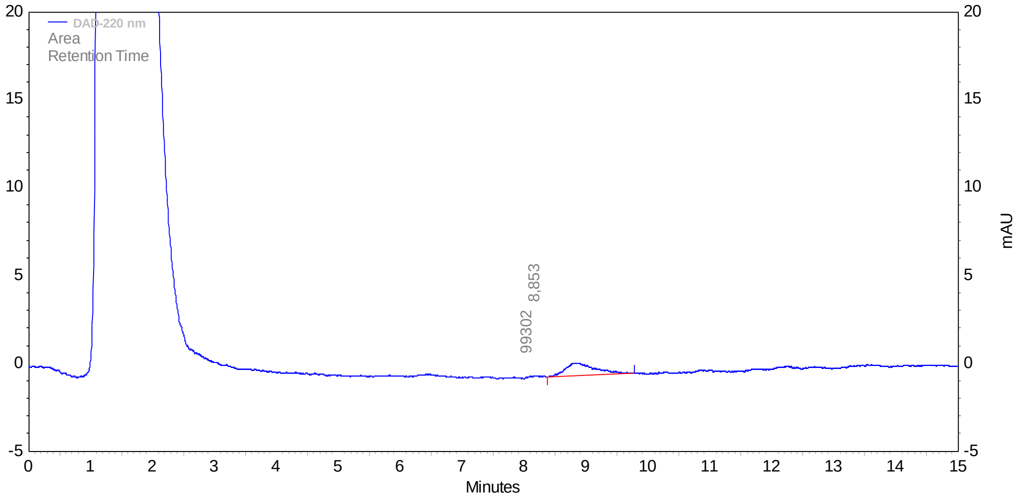

Figure S61: HPLC chromatogram of 100 µM **1a** in PBS incubated for 24 h at 37 °C.

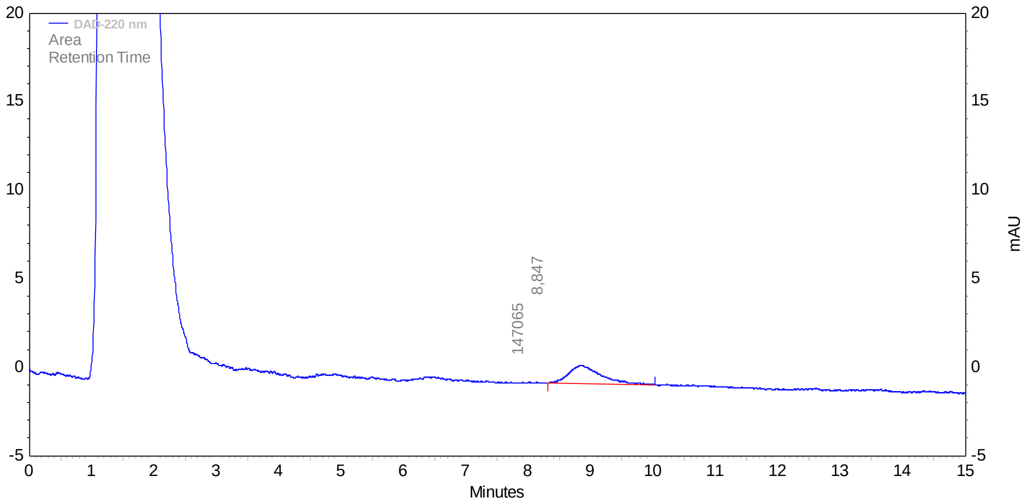

Figure S62: HPLC chromatogram of 100 µM **1b** in PBS incubated for 24 h at 37 °C.

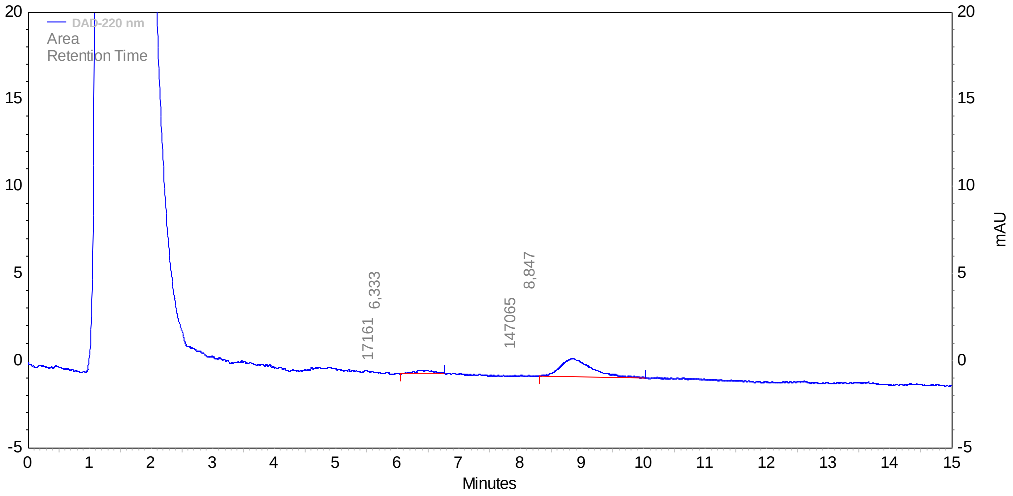

Figure S63: HPLC chromatogram of 100 µM **1c** in PBS incubated for 24 h at 37 °C.

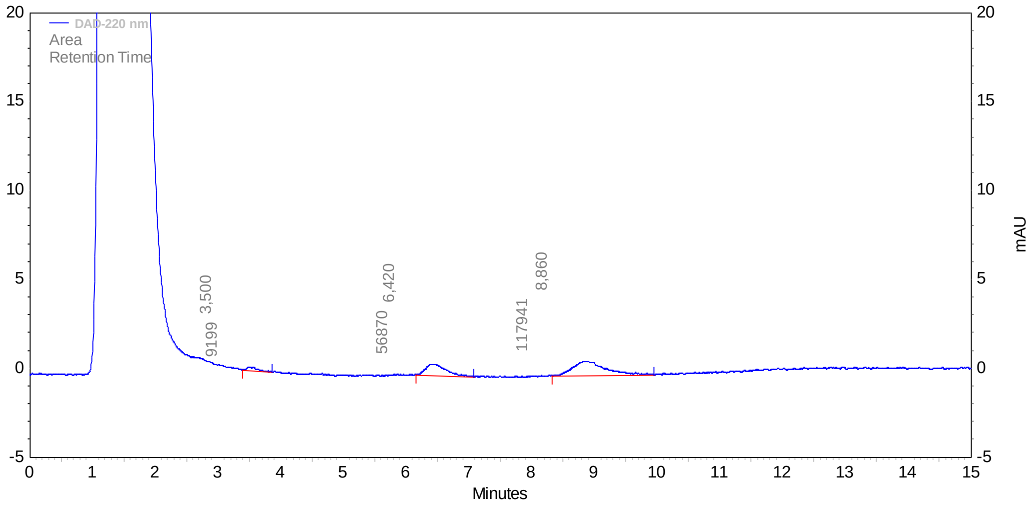

Figure S64: HPLC chromatogram of 100 µM **1a** in PBS incubated for 72 h at 37 °C.

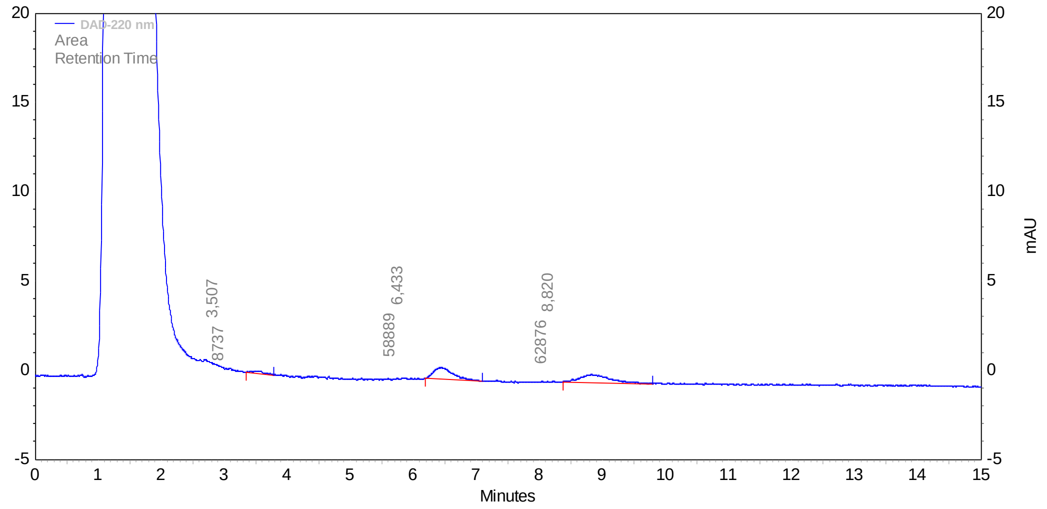

Figure S65: HPLC chromatogram of 100 µM **1b** in PBS incubated for 72 h at 37 °C.

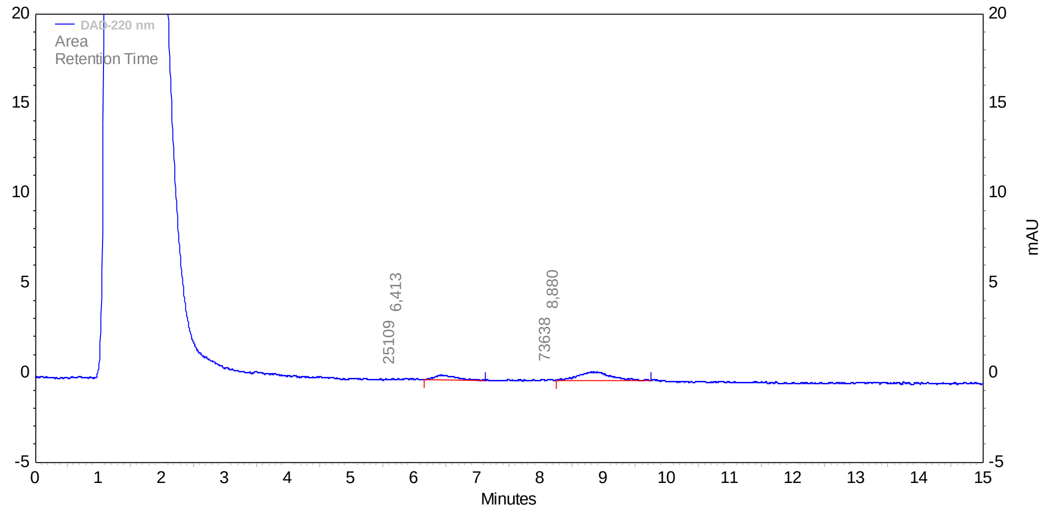

Figure S66: HPLC chromatogram of 100 µM **1c** in PBS incubated for 72 h at 37 °C.

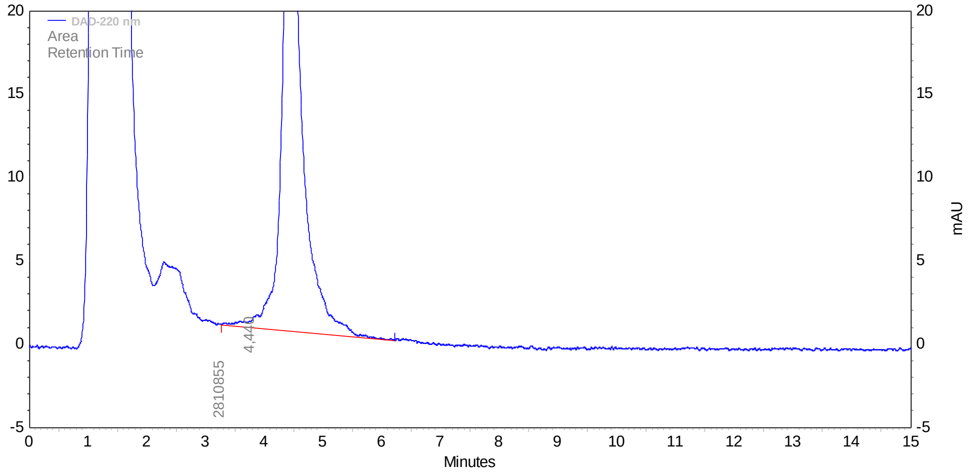

Figure S67: HPLC chromatogram of cell-culture medium incubated for 2 h at 37 °C.

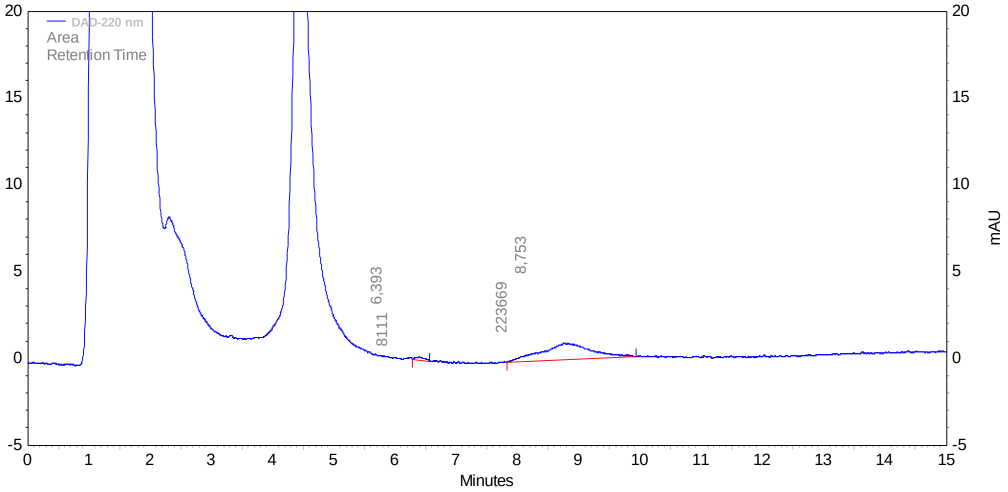

Figure S68: HPLC chromatogram of 100 µM **1a** in cell-culture medium incubated for 2 h at 37 °C.

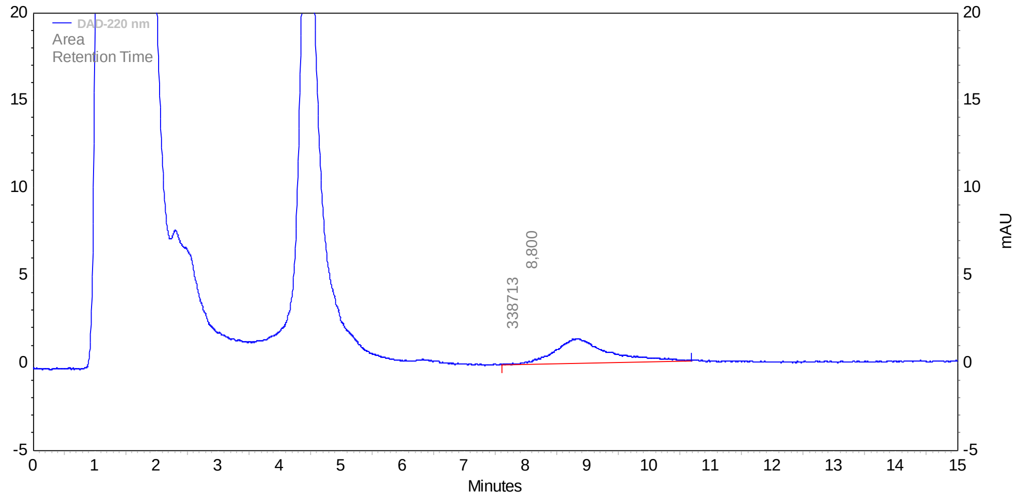

Figure S69: HPLC chromatogram of 100 µM **1b** in cell-culture medium incubated for 2 h at 37 °C.

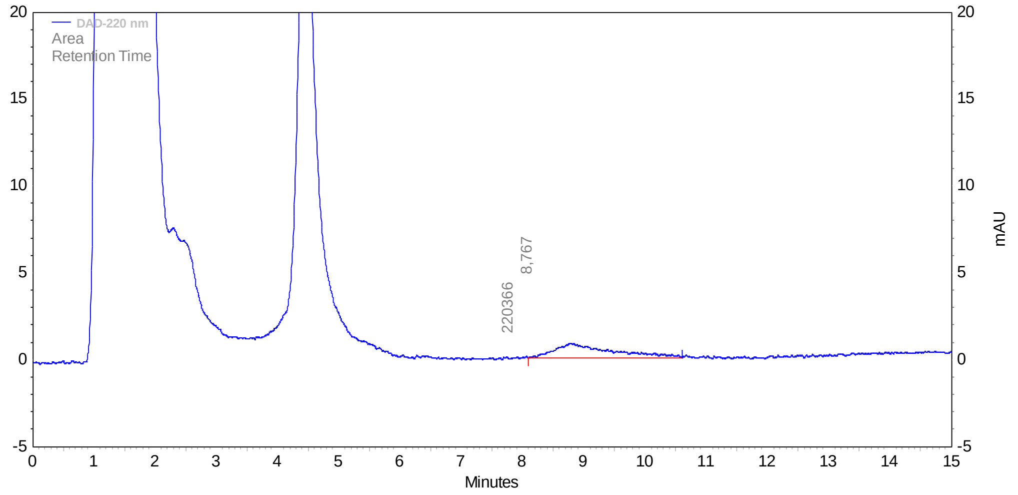

Figure S70: HPLC chromatogram of 100 µM **1c** in cell-culture medium incubated for 2 h at 37 °C.

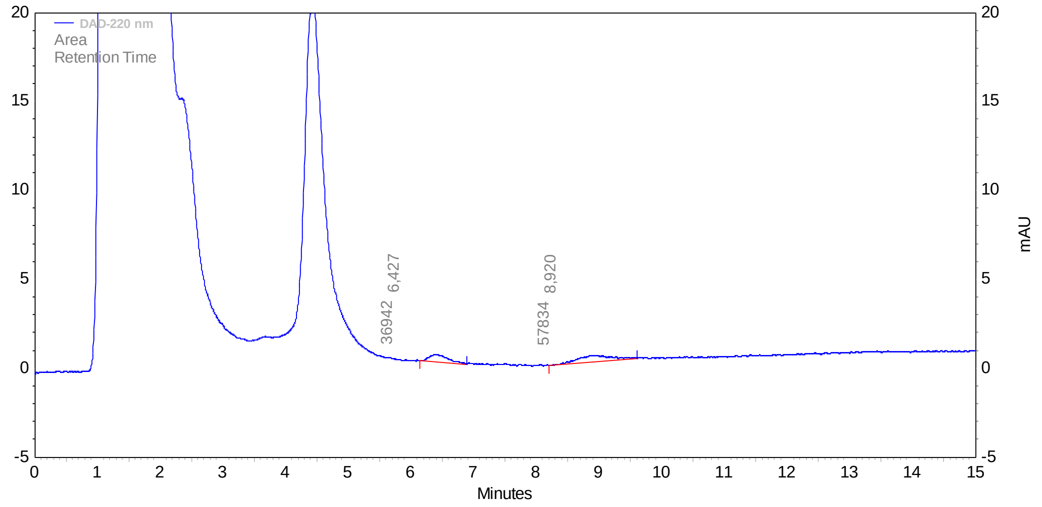

Figure S71: HPLC chromatogram of 100 µM **1a** in cell-culture medium incubated for 24 h at 37 °C.

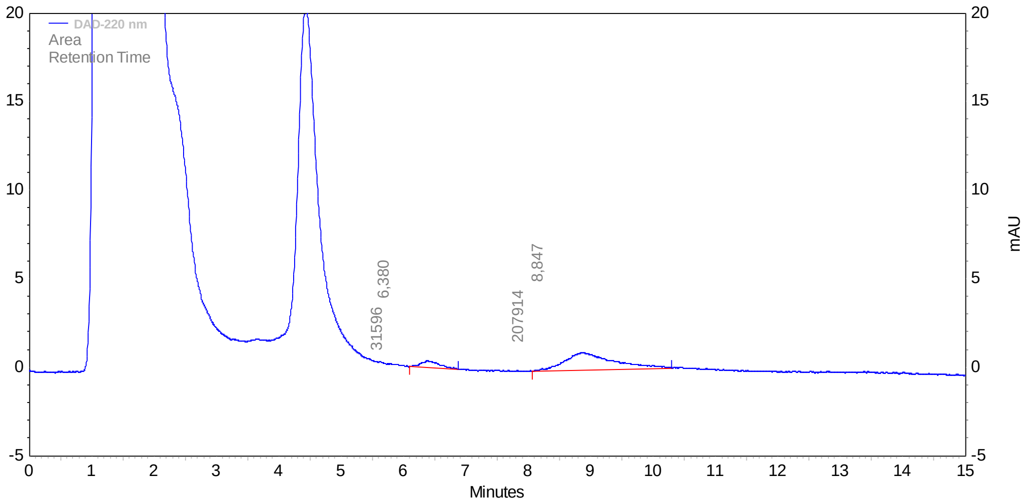

Figure S72: HPLC chromatogram of 100 µM **1b** in cell-culture medium incubated for 24 h at 37 °C.

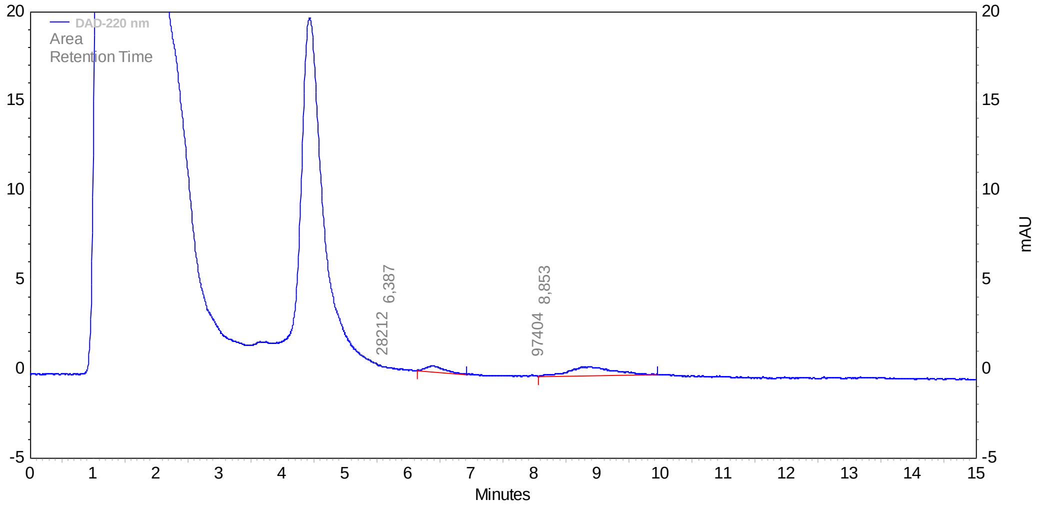

Figure S73: HPLC chromatogram of 100 µM **1c** in cell-culture medium incubated for 24 h at 37 °C.

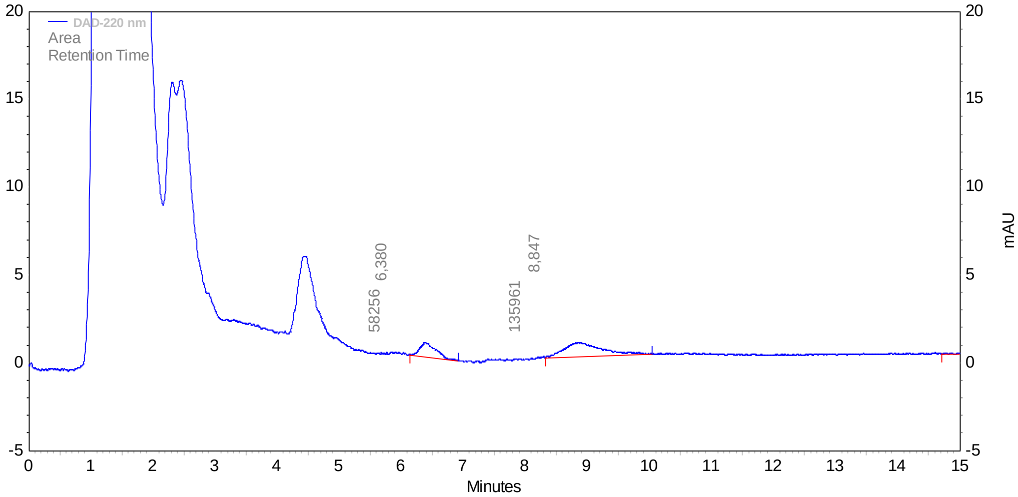

Figure S74: HPLC chromatogram of 100 µM **1a** in cell-culture medium incubated for 72 h at 37 °C.

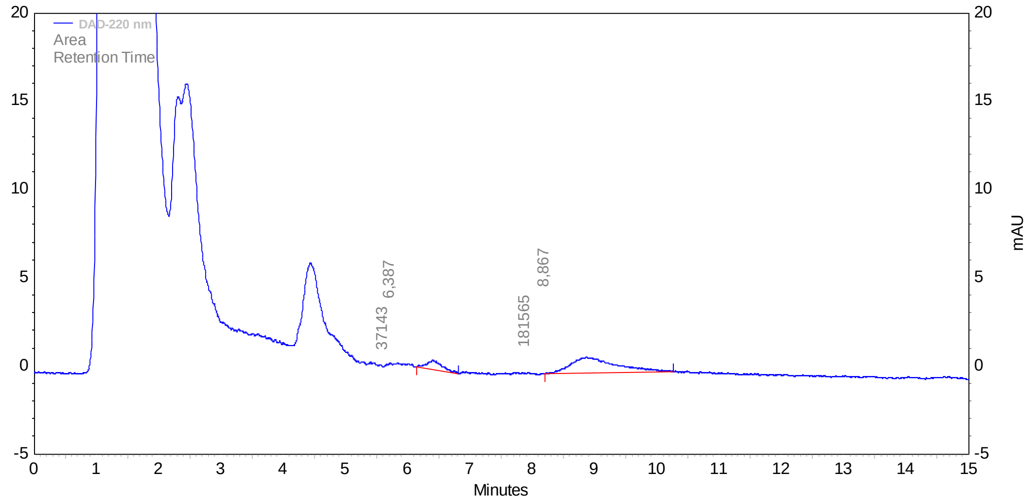

Figure S75: HPLC chromatogram of 100 µM **1b** in cell-culture medium incubated for 72 h at 37 °C.

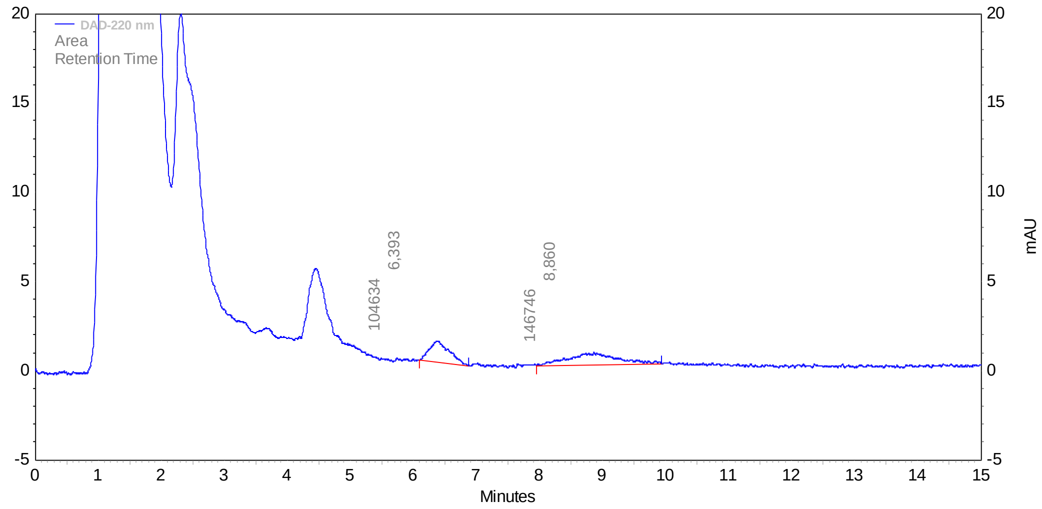

Figure S76: HPLC chromatogram of 100 µM **1c** in cell-culture medium incubated for 72 h at 37 °C.

Biology

Proliferation

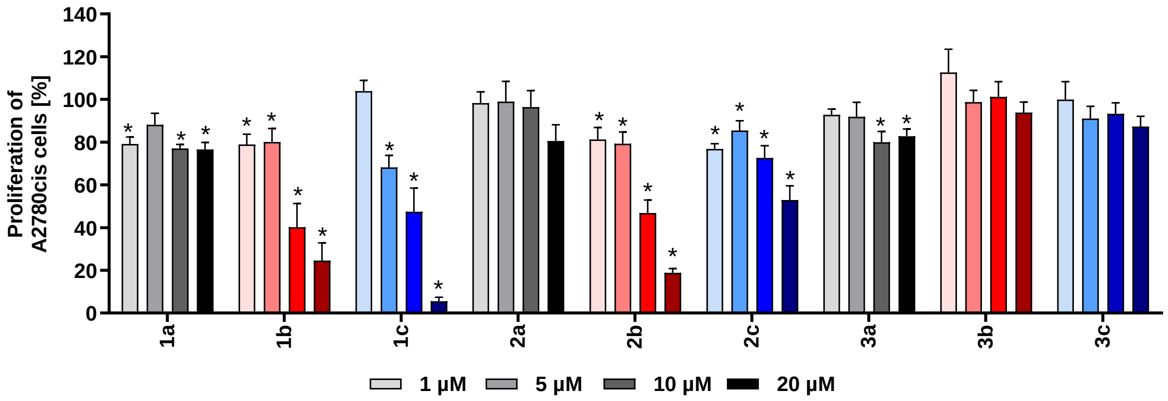

Figure S77: Proliferation of A2780cis cells after treatment with **1a-c** to **3a-c**, determined by [^{3}H]-thymidine incorporation test. The cells were incubated for 72 h with the complexes at concentrations ranging from 1 µM to 20 µM. Data are presented as the mean of 4 independent experiments + SE. Proliferation in the presence of DMSO was 81.2 + 7.1 % (data not shown). The asterisks indicate statistical significance (* $p<0.05$ against cells without complex incubation).

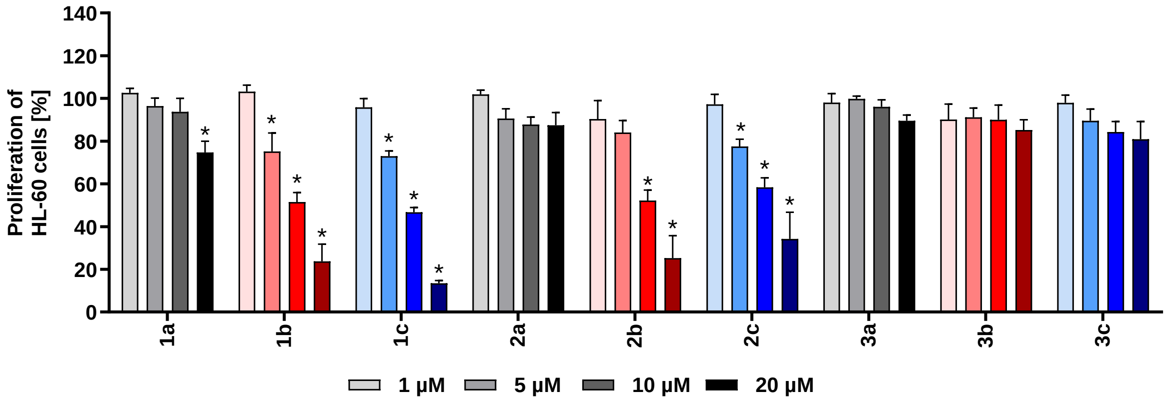

Figure S78: Proliferation of HL-60 cells after treatment with **1a-c** to **3a-c**, determined by [^{3}H]-thymidine incorporation test. The cells were incubated for 72 h with the complexes at concentrations ranging from 1 µM to 20 µM. Data are presented as the mean of 4 independent experiments + SE. Proliferation in the presence of DMSO was 95.1 + 3.6 % (data not shown). The asterisks indicate statistical significance (* $p<0.05$ against cells without complex incubation).

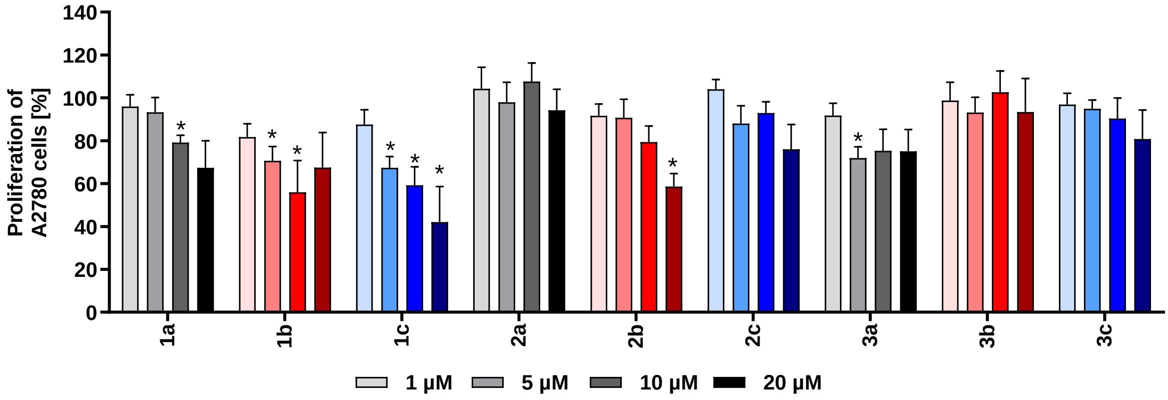

Figure S79: Proliferation of A2780 cells after treatment with **1a-c** to **3a-c**, determined by [^{3}H]-thymidine incorporation test. The cells were incubated for 72 h with the complexes at concentrations ranging from 1 µM to 20 µM. Data are presented as the mean of 5 independent experiments + SE. Proliferation in the presence of DMSO was 88.1 + 8.3 % (data not shown). The asterisks indicate statistical significance (* p<0.05 against cells without complex incubation).

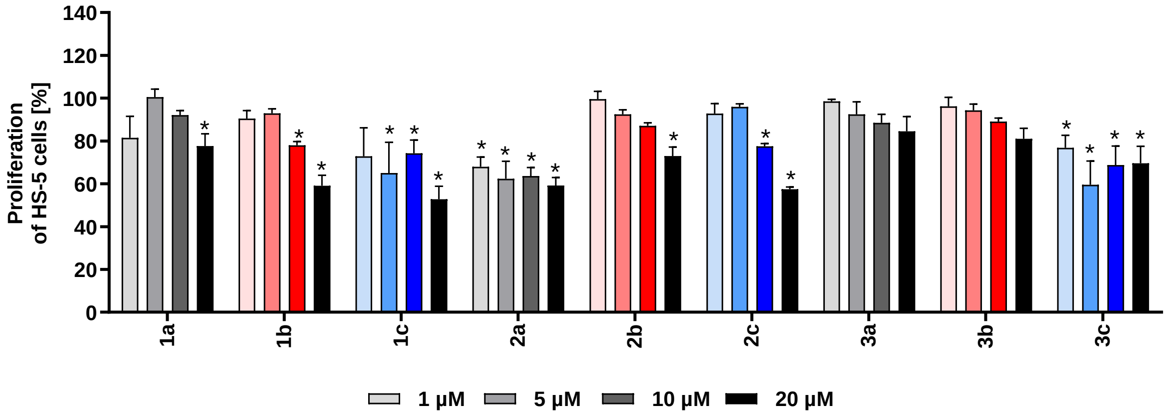

Figure S80: Proliferation of HS-5 cells after treatment with **1a-c** to **3a-c**, determined by [^{3}H]-thymidine incorporation test. The cells were incubated for 72 h with the complexes at concentrations ranging from 1 µM to 20 µM. Data are presented as the mean of 4 independent experiments + SE. Proliferation in the presence of DMSO was 93.8 + 2.6 % (data not shown). The asterisks indicate statistical significance (* p<0.05 against cells without complex incubation).

Metabolic activity

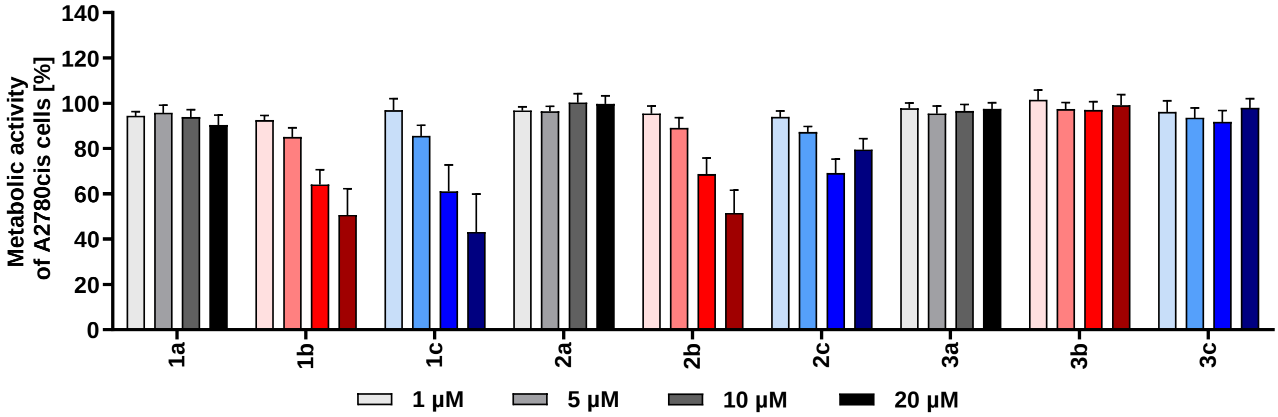

Figure S81: Metabolic activity of A2780cis cells after treatment with **1a-c** to **3a-c**, determined by the modified MTT assay. The cells were incubated for 72 h with the complexes at concentrations ranging from 1 µM to 20 µM. Data are presented as the mean of 7 independent experiments + SE. Metabolic activity in the presence of DMSO was 99.1 ± 2.3% (data not shown). The asterisks indicate statistical significance (* $p<0.05$ against cells without complex incubation).

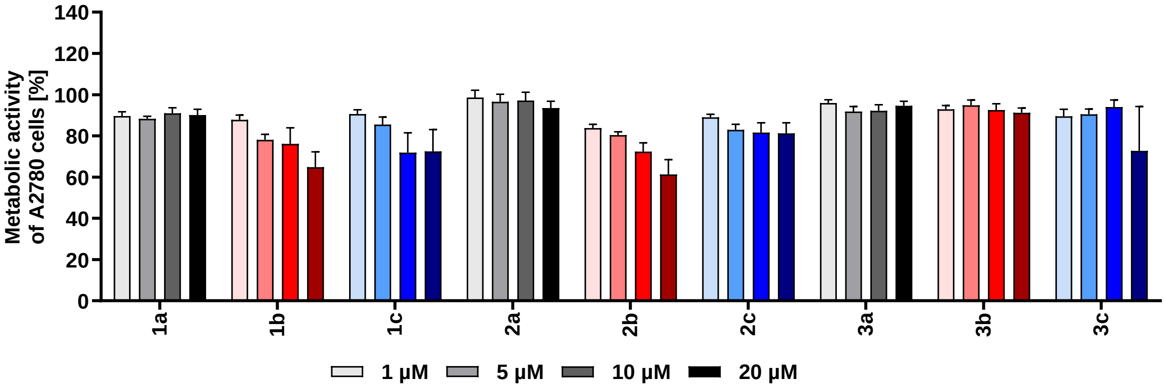

Figure S82: Metabolic activity of A2780 cells after treatment with **1a-c** to **3a-c**, determined by the modified MTT assay. The cells were incubated for 72 h with the complexes at concentrations ranging from 1 µM to 20 µM. Data are presented as the mean of 6 independent experiments + SE. Metabolic activity in the presence of DMSO was 94.3 + 1.4% (data not shown). The asterisks indicate statistical significance (* $p<0.05$ against cells without complex incubation).

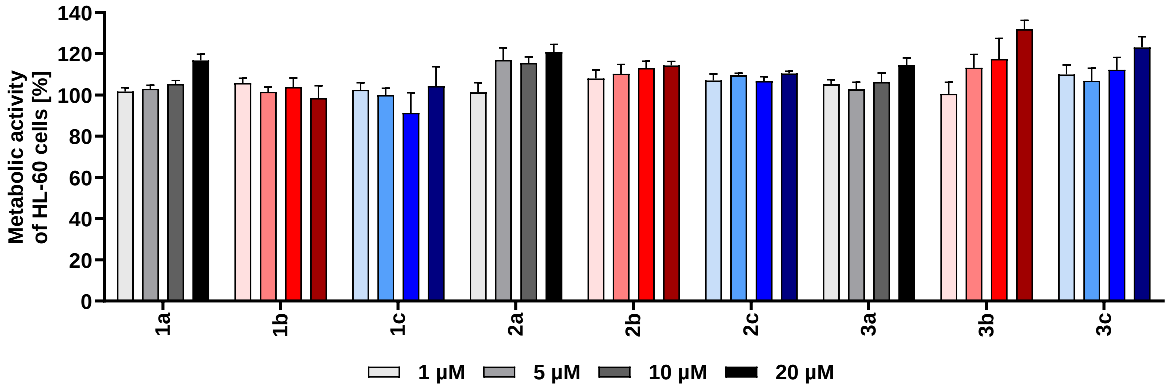

Figure S83: Metabolic activity of HL-60 cells after treatment with **1a-c** to **3a-c**, determined by the modified MTT assay. The cells were incubated for 72 h with the complexes at concentrations ranging from 1 µM to 20 µM. Data are presented as the mean of 4 independent experiments + SE. Metabolic activity in the presence of DMSO was 99.3 + 2.0% (data not shown). The asterisks present statistical significance (* $p<0.05$ against cells without complex incubation).

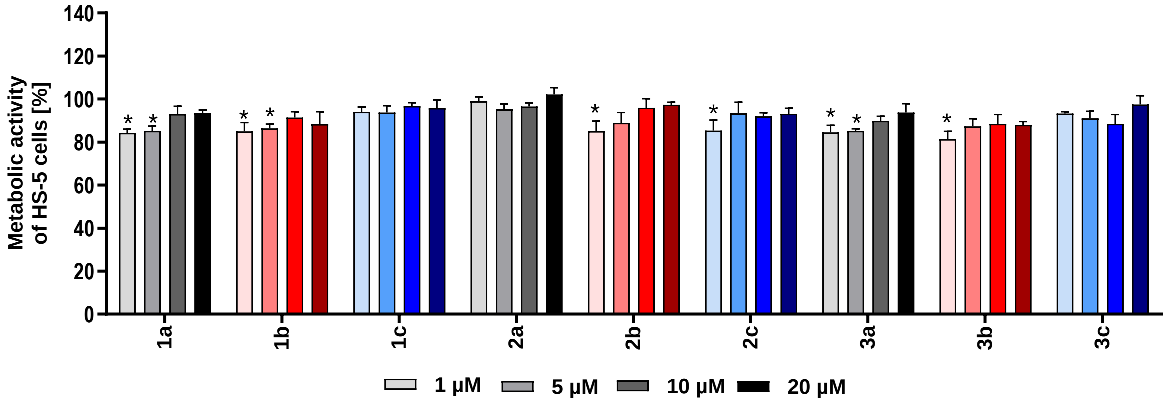

Figure S84: Metabolic activity of HS-5 cells after treatment with **1a-c** to **3a-c**, determined by the modified MTT assay. The cells were incubated for 72 h with the complexes at concentrations ranging from 1 µM to 20 µM. Data are presented as the mean of 4 independent experiments + SE. Metabolic activity in the presence of DMSO was 93.8 + 2.6% (data not shown). The asterisks present statistical significance (* $p<0.05$ against cells without complex incubation).

Scratch-Assay

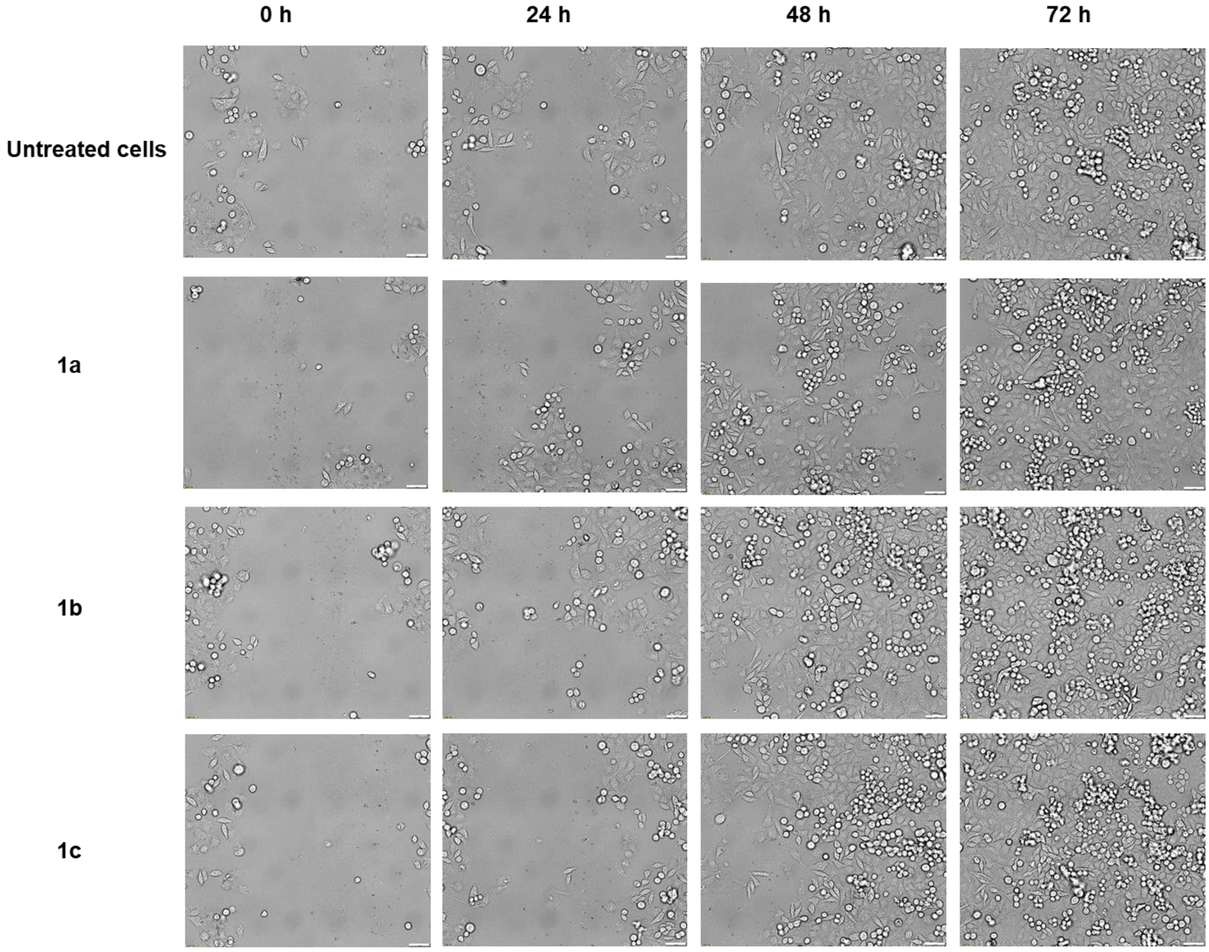

Figure S85: Cell migration investigated in a scratch assay. MDA-MB 231 were untreated (1st row) or incubated for 72 h with **1a** (2nd row), **1b** (3rd row) and **1c** (4th row), at a concentration of 1 µM, respectively. Pictures after 0 h, 24 h, 48 h and 72 h (columns) are shown. Scale bar = 50 µM.

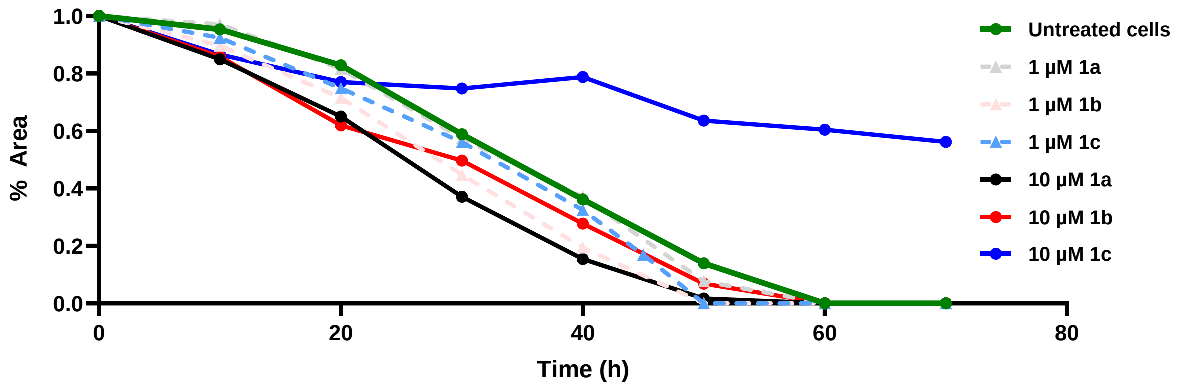

Figure S86: Numerical analysis of scratch closure in the wound healing assay depicted in Figure S85 by Olympus cellSENS Dimension Desktop 4.2.1. software. The area of the scratch of each well 5 minutes after addition of the complexes was set at 1.0 %.

Cell viability and cell death study

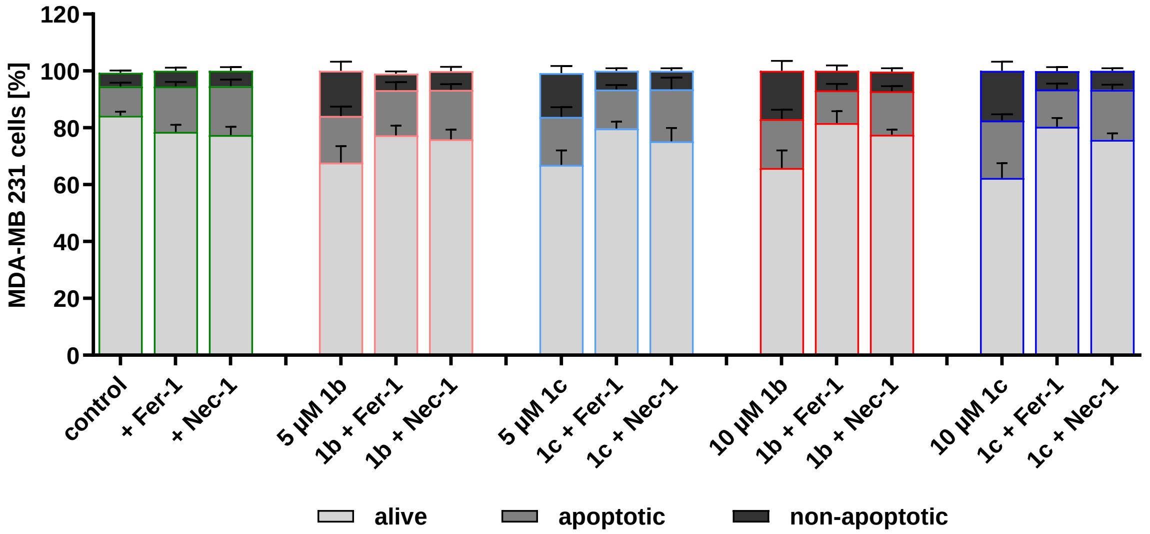

Figure S87: Cell viability and cell death after **1b** and **1c** treatment. MDA-MB 231 cells were incubated for 24 h with the complexes **1b** or **1c** (at concentrations of 5 µM and 10 µM, respectively) alone or in combination with the inhibitors Fer-1 (1 µM) or Nec-1 (20 µM). Subsequent annexin V (AnV) and propidium iodide (PI) staining as well as flow cytometry analysis were performed to select alive (AnV-/PI-), apoptotic (AnV+/PI-) and non-apoptotic dead (AnV+/PI+, PI+) cells. Data are presented as mean of 4 experiments + SE.
